# Supplementary material for: Bacterial outer membrane proteins assemble via asymmetric interactions with the BamA β-barrel
Source: Nat Commun. 2019 Jul 26;10:3358. doi: 10.1038/s41467-019-11230-9 (PMC6659671; doi:10.1038/s41467-019-11230-9)
Supplement: Supplementary file 1 — Supplementary Information [file 41467_2019_11230_MOESM1_ESM.pdf]

Supplementary Information

Bacterial outer membrane proteins assemble via asymmetric interactions with the BamA β-barrel

Matthew T. Doyle and Harris D. Bernstein

Correspondence and requests for materials should be addressed to H.D.B.
(email: harris_bernstein@nih.gov)

CONTENTS

Supplementary Fig. 1: Design of a stable OMP assembly intermediate and optimisation of expression. **(pg. 2)**

Supplementary Fig. 2: The native disulphide-bond in BamA loop 6 is required for complete folding. **(pg. 4)**

Supplementary Fig. 3: Controls for disulphide-bond formation assays described in Fig. 2. **(pg. 5)**

Supplementary Fig. 4: $^{MBP\text{-}76}$EspP-BamA hybrid intermediates remain assembly competent after disulphide-bond formation between BamA(β1) and the β-signal. **(pg. 6)**

Supplementary Fig. 5: Screen for potential interactions between the $^{MBP\text{-}76}$EspP β-signal and ^{His}BamA(β15/β16). **(pg. 8)**

Supplementary Fig. 6: Additional screens for potential interactions between cysteines in $^{MBP\text{-}76}$EspP(β1) and luminal cysteines in ^{His}BamA(β16). **(pg. 9)**

Supplementary Fig. 7: Controls for disulphide bond formation assays described in Fig. 4. **(pg. 11)**

Supplementary Fig. 8: Disulphide-bond formation assay reduction controls for Fig. 5b. **(pg. 13)**

Supplementary Fig. 9: Raw data and control experiments for Fig. 6. **(pg. 14)**

Supplementary Table 1: ANOVA and multiple comparisons tests for disulphide-bond formation assays shown in Fig. 2e. **(pg. 15)**

Supplementary Table 2: ANOVA and multiple comparisons tests for disulphide-bond formation assays shown in Fig. 3c. **(pg. 16)**

Supplementary Table 3: ANOVA and multiple comparisons tests for disulphide-bond formation assays shown in Fig. 4c. **(pg. 16)**

Supplementary Table 4: ANOVA and multiple comparisons tests for disulphide-bond formation assays shown in Fig. 4e. **(pg. 17)**

Supplementary Table 5: ANOVA and multiple comparisons tests for disulphide-bond formation assays shown in Fig. 5. **(pg. 17)**

Supplementary Table 6: Plasmids used in this study. **(pg. 18)**

Supplementary Table 7: Oligonucleotides and double-stranded DNA fragments used in this study. **(pg. 20)**

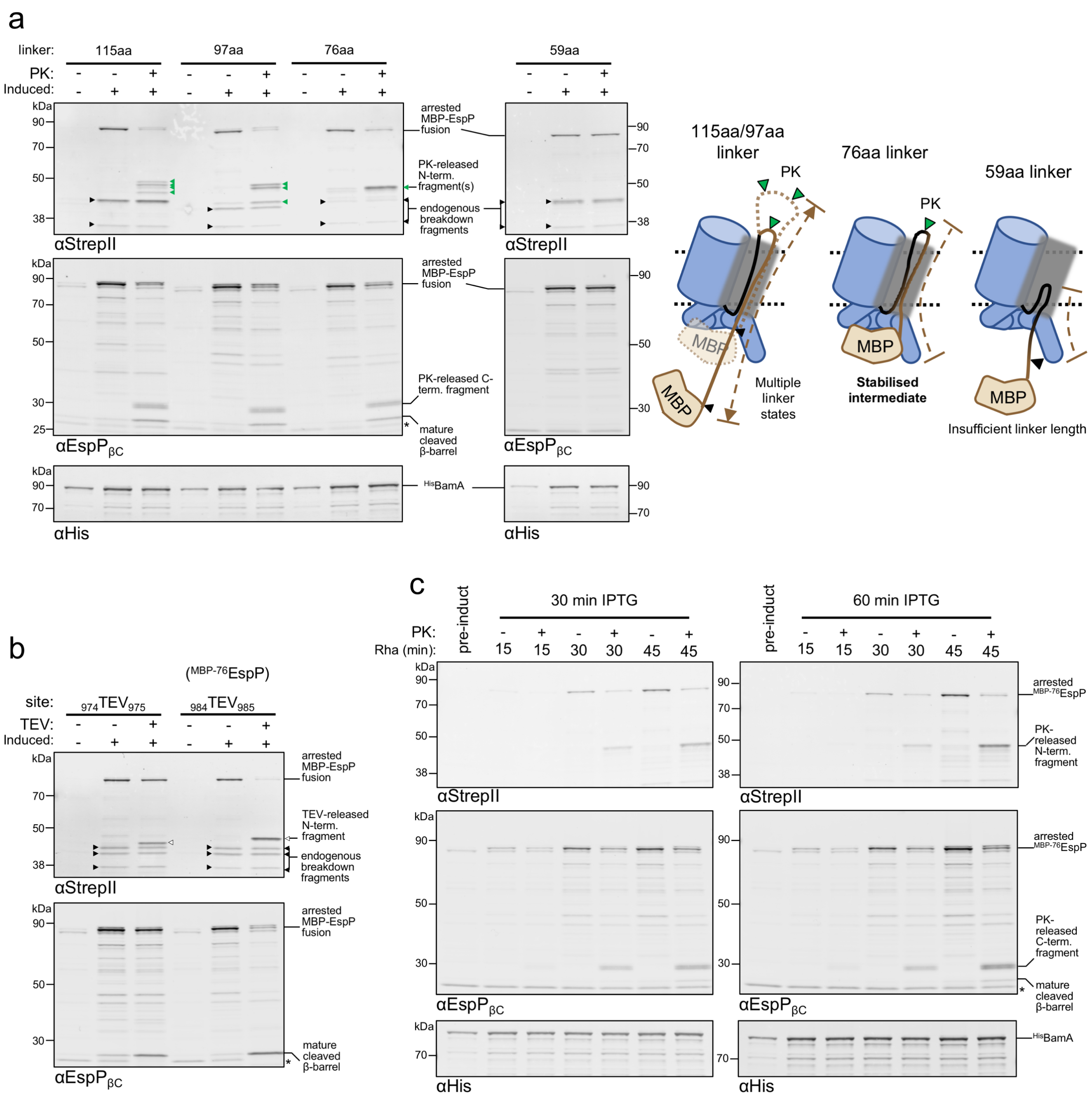

Supplementary Fig. 1 (legend next page)

Supplementary Fig. 1: Design of a stable OMP assembly intermediate and optimisation of expression.

a, *E. coli* BL21(DE3) were transformed with plasmids that encode ^{His}BamABCDE (pMTD372) and an MBP fusion to truncated versions of EspP containing a 115, 97, 76 or 59aa-linker (pMTD321, pMTD501, pMTD502, or pMTD610, respectively). ^{His}BamABCDE expression was induced for 30 min with 0.4 mM IPTG, and then fusion protein expression was induced for 45 min with 0.2% rhamnose. Cells were proteinase K (PK)-treated or mock-treated on ice for 20 min. Immunoblots were then conducted using the indicated antibodies/antisera. A cross-reactive protein is denoted (*). The fusion proteins containing 115, 97 and 59aa-linkers were susceptible to endogenous periplasmic proteases that generated N-terminal MBP-containing breakdown products (black arrowheads), while the degradation of the 76aa-linker variant was dramatically reduced. All of the fusion proteins except the 59aa-linker variant were susceptible to cleavage by PK. PK treatment generated multiple N-terminal MBP-containing fragments from the 115 and 97aa-linker variants (green arrowheads) but only one N-terminal fragment from the 76aa-linker variant. The results suggest that 1) the linkers of the two largest variants have multiple conformations that cause instability and degradation, 2) the 76aa-linker variant has a single stable conformation, and 3) the 59aa-linker variant is unstable because the linker is too short to enter the translocation channel (see cartoon on right). **b**, BL21(DE3) were transformed with pMTD372 and a plasmid that encodes a derivative of the 76aa-linker variant that contains a TEV site between EspP residues 974/975 or 984/985 (pMTD521 or pMTD607, respectively). The expression of ^{His}BamABCDE and the fusion protein was induced as in **a**. Cells were TEV-treated or mock-treated for 2 h at 25 °C and samples immunoblotted. Because the TEV site of the $_{984}$TEV$_{985}$ variant was significantly more surface exposed, the fusion protein containing this modification was used throughout this work and named $^{MBP-76}$EspP. **c**, Optimization of $^{MBP-76}$EspP expression and BAM engagement. BL21(DE3) were transformed with pMTD372 and pMTD607. ^{His}BamABCDE expression was induced with 0.4 mM IPTG for either 30 min or 60 min before $^{MBP-76}$EspP expression was induced by adding 0.2% rhamnose (Rha) for 15, 30 or 45 min. Cells were PK-treated and immunoblotted as in **a**. An induction protocol of 60 min IPTG followed by 45 min Rha was chosen for all subsequent experiments in this work.

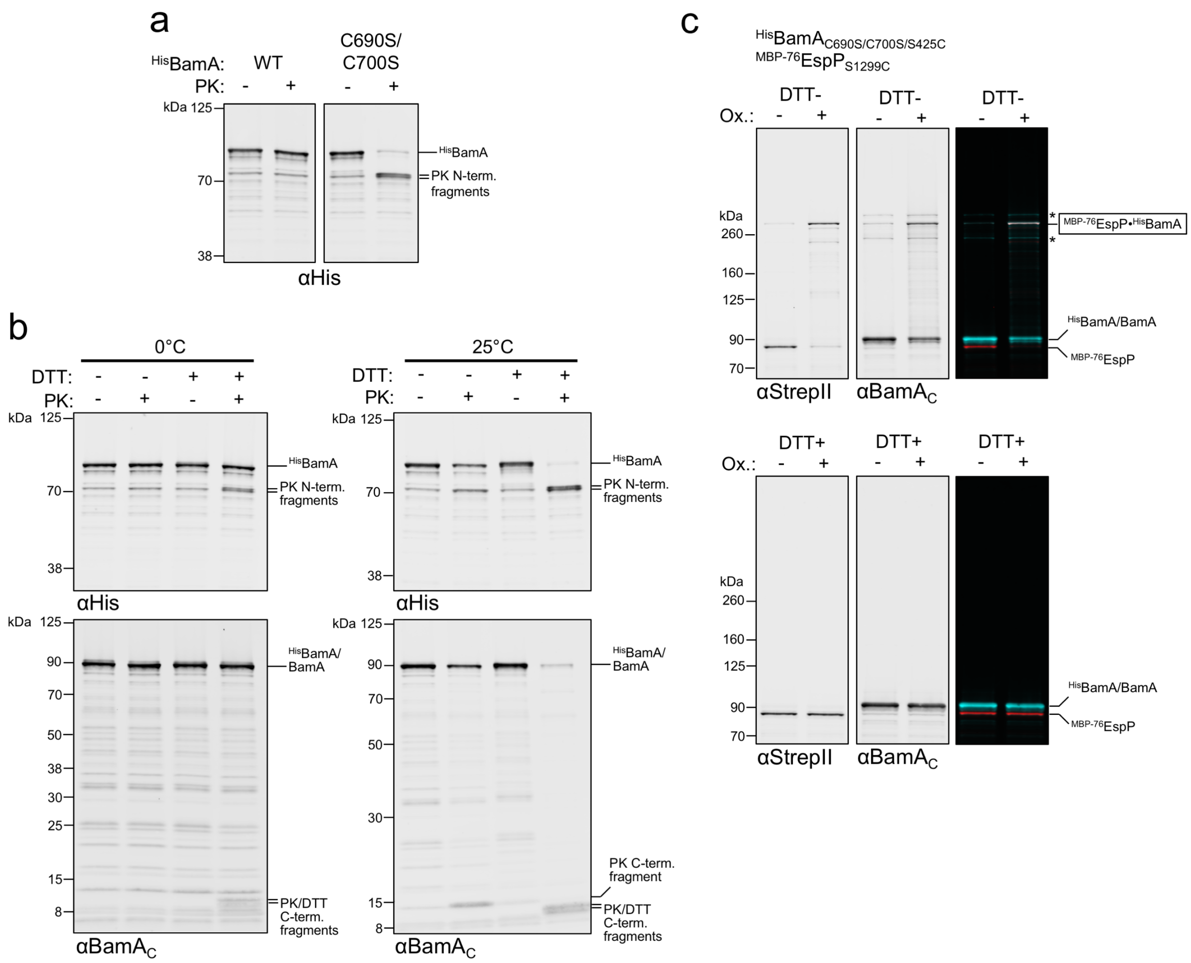

Supplementary Fig. 2: The native disulphide-bond in BamA loop 6 is required for complete folding.

a, BL21(DE3) expressed ^{His}BamABCDE with either wild type or ^{His}BamA or the ^{His}BamA$_{C690S/C700S}$ double mutant. Cells were treated with PK or mock-treated on ice for 20 min. Immunoblots were then conducted using an anti-His antibody. N-terminal PK fragments were only observed for the mutant. **b**, Cells expressing wild type ^{His}BamABCDE complex were digested (or mock treated) with PK for 30 min on ice (0 °C) or at 25 °C in the presence or absence of 150 mM DTT. PK sensitivity was mostly dependent on the presence of the reducing agent unless digestion was performed at the higher temperature. The molecular weights of N- and C-terminal BamA PK fragments are consistent with the digestion of loop 6. **c**, BL21(DE3) that expressed $^{MBP\text{-}76}$EspP$_{S1299C}$ and ^{His}BamA$_{C690S/C700S/S425C}$BCDE were mock-treated (-) or treated with 4-DPS (+). Duplex-immunoblots were then conducted using antibodies/antisera against the N-terminus of $^{MBP\text{-}76}$EspP (αStrepII, red) and the C-terminus of ^{His}BamA/BamA (αBamA$_C$, cyan) to monitor disulphide-bond formation *in vivo*. Cross-reactive proteins are denoted (*). The level of disulphide-bond formation was similar to that observed in our analysis of the $^{MBP\text{-}76}$EspP$_{S1299C}$/^{His}BamA$_{S425C}$ pair (Fig. 2b). The presence of cysteines in BamA loop 6 therefore does not interfere with the interpretation of our results. Because C690 and C700 enable BamA to fold into its native state, we opted to use the wild-type protein throughout our study. Data are representative of at least two independent experiments.

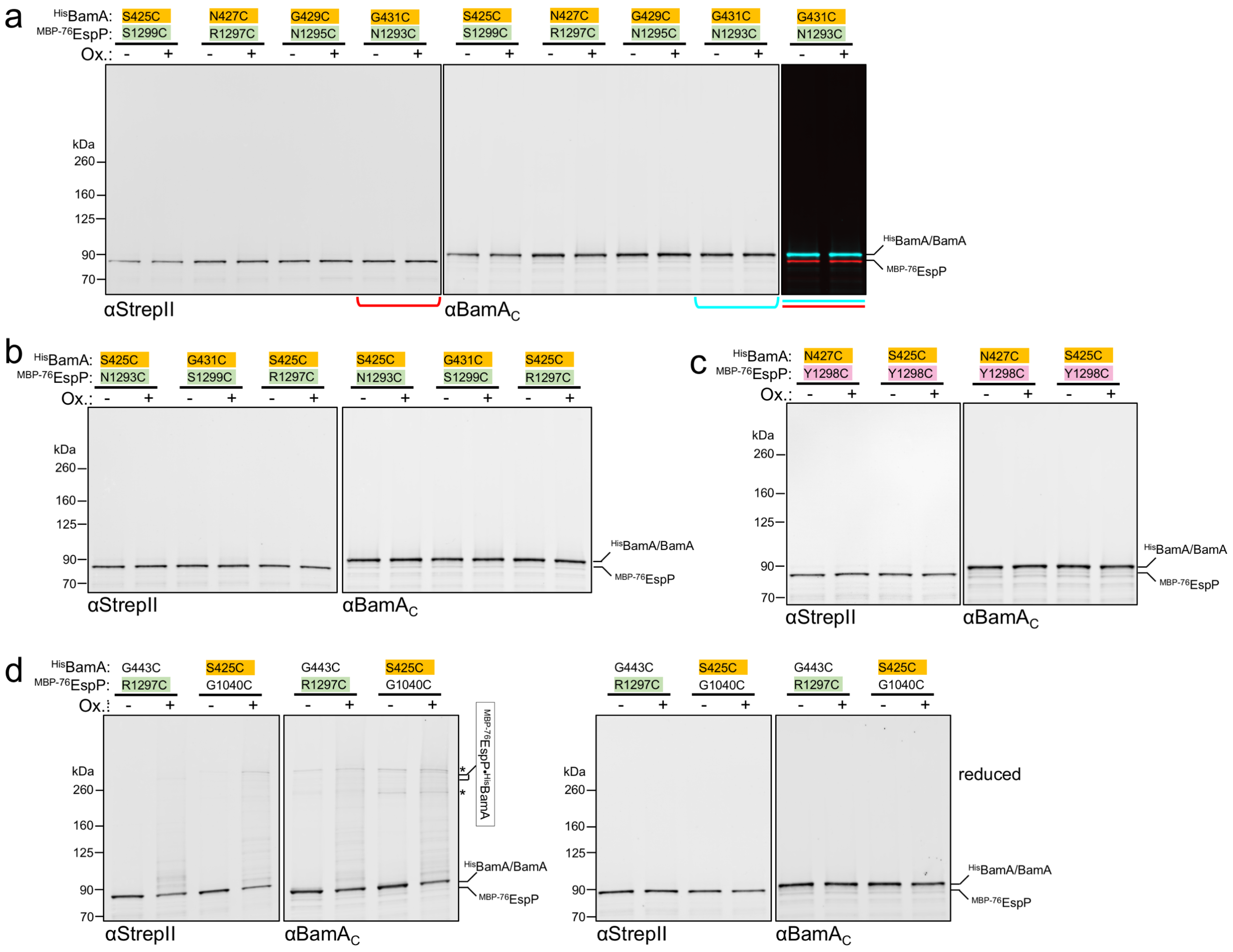

Supplementary Fig. 3: Controls for disulphide-bond formation assays described in Fig. 2. **a,b,c**, Duplex-immunoblots were performed on reduced samples from the assays in Fig. 2b (**a**), Fig. 2c (**b**), and Fig. 2d (**c**) using antibodies/antisera against the N-terminus of $^{MBP\text{-}76}$EspP (αStrepII, red) and the C-terminus of ^{His}BamA/BamA (αBamA$_C$, cyan). The results show that high molecular weight species were due to the formation of specific intermolecular disulphide-bonds. **d**, BL21(DE3) that expressed $^{MBP\text{-}76}$EspP$_{R1297C}$/^{His}BamA$_{G443C}$ [to test the proximity of a luminal residue in EspP (β12) and a register-paired luminal residue in ^{His}BamA(β2] or $^{MBP\text{-}76}$EspP$_{G1040C}$/^{His}BamA$_{S425C}$ [to test the proximity of BamA(β1) and $^{MBP\text{-}76}$EspP(β1)] were mock-treated (-) or treated with 4-DPS (+). Samples were analysed by duplex immunoblotting. The samples on the right were reduced prior to SDS-PAGE. Cross-reactive proteins are denoted (*). Data are representative of at least two independent experiments.

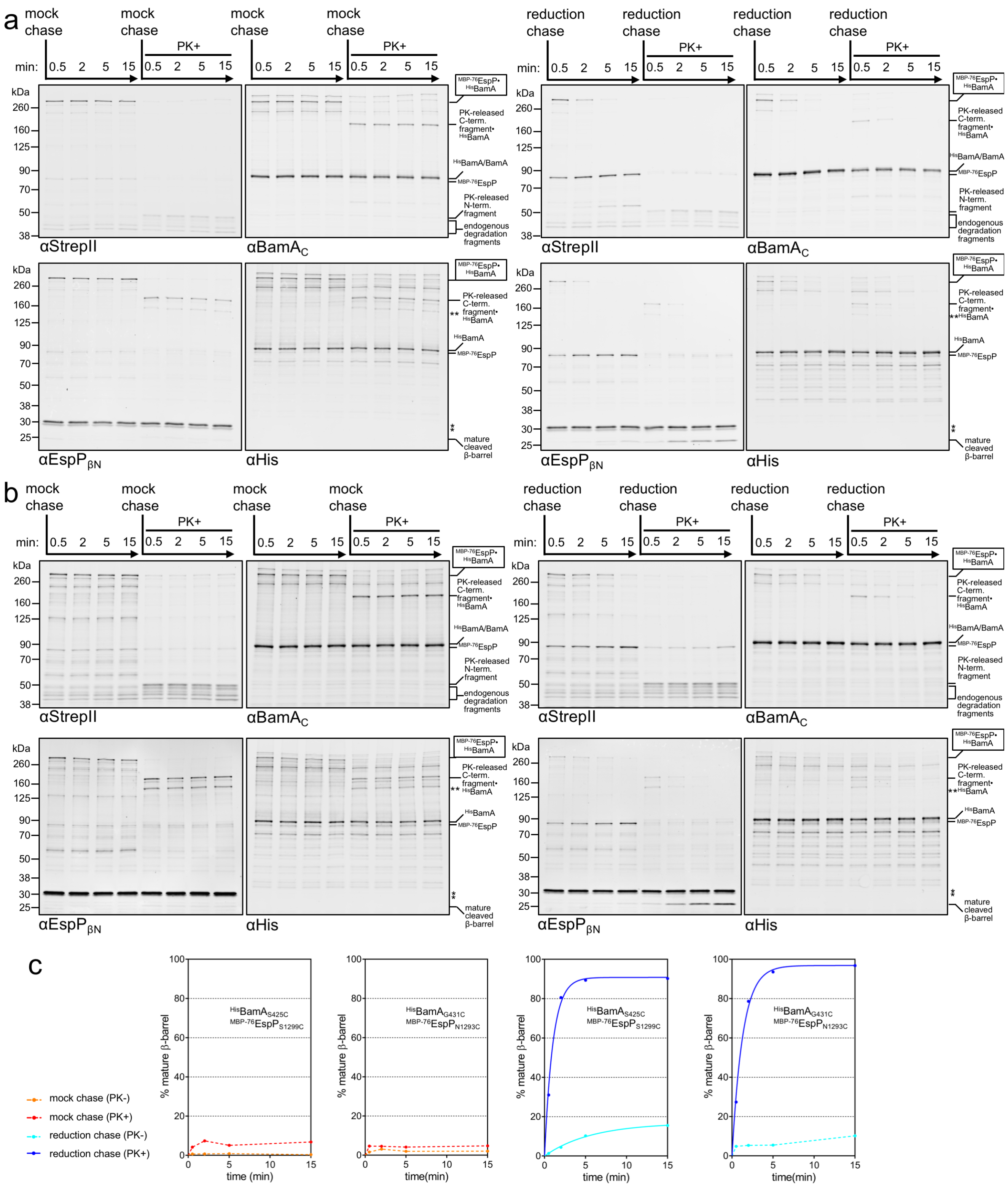

Supplementary Fig. 4 (legend next page)

Supplementary Fig. 4: $^{MBP\text{-}76}$EspP-BamA hybrid intermediates remain assembly competent after disulphide-bond formation between BamA(β1) and the β-signal.

a, b, BL21(DE3) that expressed $^{MBP\text{-}76}$EspP$_{S1299C}$/^{His}BamA$_{S425C}$ (**a**) or $^{MBP\text{-}76}$EspP$_{N1293C}$/^{His}BamA$_{G431C}$ (**b**) were treated with 4-DPS, mock-treated or PK-treated on ice, and then incubated for 0.5-15 min at 25° C in the absence or presence of DTT ('mock chase' or 'reduction chase'). Duplex-immunoblots were then conducted using αStrepII and αBamA$_C$, or αEspP$_{βN}$ and αHis. The results show that β-barrel assembly restarts following the release of MBP and reduction of the disulphide-bond between the fusion protein and BamA. Non-specific bands are denoted (*). A C-terminal PK-fragment of $^{MBP\text{-}76}$EspP that is disulphide-bonded to an N-terminal PK-fragment of ^{His}BamA (see Supplementary Fig. 2) is also indicated (**). **c,** The fraction of the $^{MBP\text{-}76}$EspP that was completely assembled (% mature β-barrel) was calculated using the signal from αEspP$_{βN}$ in **a**. Representative results from at least two independent experiments are shown.

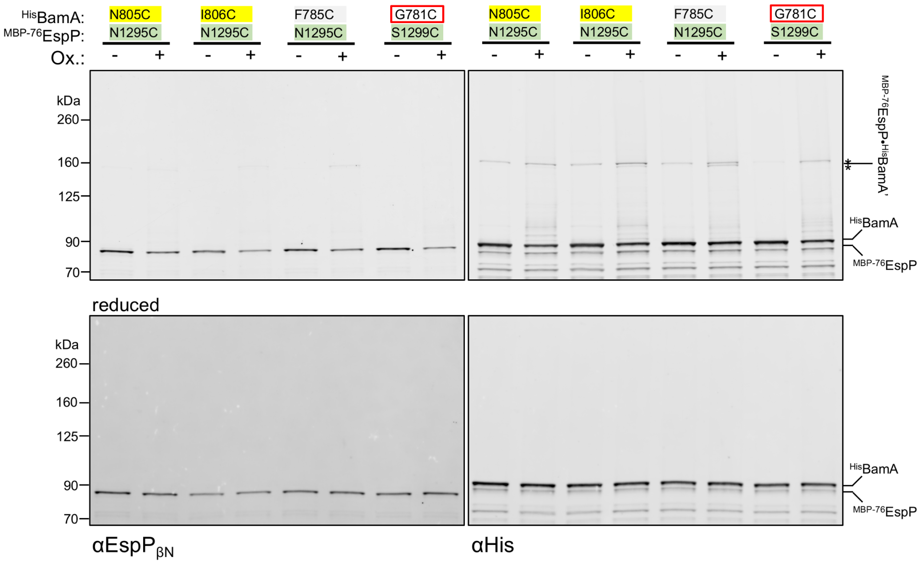

Supplementary Fig. 5: Screen for potential interactions between the $^{MBP\text{-}76}$EspP β-signal and ^{His}BamA(β15/β16).

BL21(DE3) that expressed $^{MBP\text{-}76}$EspP with a cysteine substitution in β12 and ^{His}BamABCDE with a cysteine substitution in ^{His}BamA β15 or β16 were mock-treated (-) or treated with 4-DPS (+). Duplex-immunoblots were then conducted using antibodies/antisera against the N-terminus of the $^{MBP\text{-}76}$EspP β-barrel domain (αEspP$_{βN}$) and the N-terminus of ^{His}BamA (αHis) to monitor disulphide-bond formation *in vivo*. Note that while $^{MBP\text{-}76}$EspP•^{His}BamA disulphide-bonded adducts usually migrate at or above 260 kDa, only very low levels of an unusually fast migrating ~160 kDa adduct ($^{MBP\text{-}76}$EspP•^{His}BamA') were observed. A quantitative analysis based on the αEspP$_{βN}$ signal from repeated experiments showed that the level of $^{MBP\text{-}76}$EspP•^{His}BamA' was as follows: $^{MBP\text{-}76}$EspP$_{N1295C}$/^{His}BamA$_{N805C}$ = 0.2%, $^{MBP\text{-}76}$EspP$_{N1295C}$/^{His}BamA$_{I806C}$ = 2.2-2.7%, $^{MBP\text{-}76}$EspP$_{N1295C}$/^{His}BamA$_{F785C}$ = 2.9-5%, $^{MBP\text{-}76}$EspP$_{S1299C}$/^{His}BamA$_{G781C}$ = 0.1%, . Samples that were reduced prior to SDS-PAGE are shown on the lower immunoblots. Non-specific bands are denoted (*). Data are representative of two independent experiments.

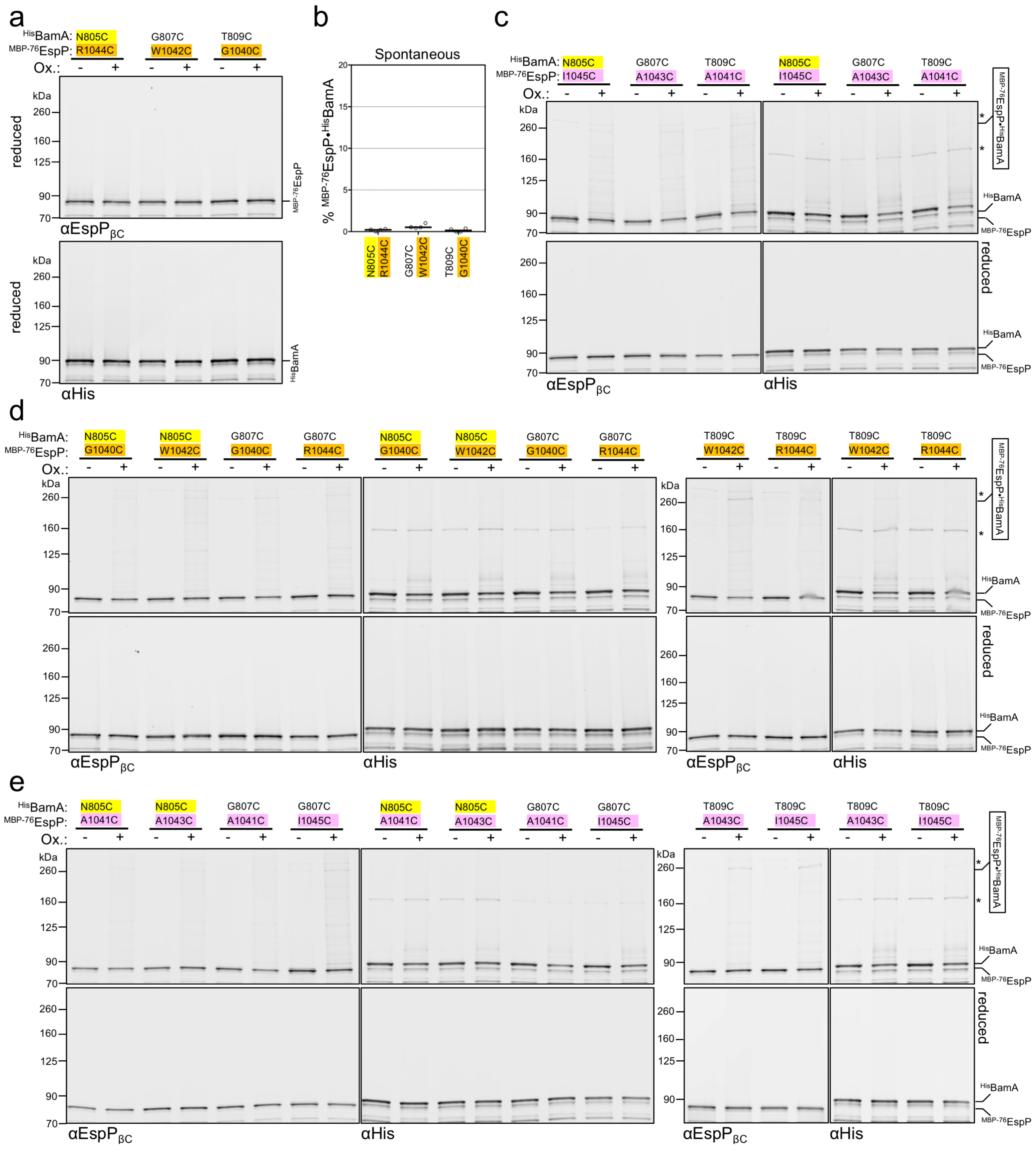

Supplementary Fig. 6 (legend next page)

Supplementary Fig. 6: Additional screens for potential interactions between cysteines in $^{MBP\text{-}76}$EspP(β1) and luminal cysteines in ^{His}BamA(β16).

a, Duplex-immunoblots were performed on reduced samples from the assays in Fig. 3b using antibodies/antisera against the C-terminus of $^{MBP\text{-}76}$EspP (αEspP$_{βC}$) and the N-terminus of ^{His}BamA (αHis). **b,** Quantitation of 'spontaneous' disulphide-bond formation between $^{MBP\text{-}76}$EspP(β1)-^{His}BamA(β16) cysteine-pairs in cells that were mock-treated in experiments shown in Fig. 3c. Only αStrepII was used for probing immunoblots. Bars = median, N= 4. **c,** Experiments shown in Fig. 3b were repeated except that cysteine substitutions at aligned lipid-facing positions in $^{MBP\text{-}76}$EspP(β1) and luminal-facing positions of ^{His}BamA(β16) were analysed. Samples that were reduced prior to SDS-PAGE are shown on the lower immunoblots. **d, e,** Experiments shown in Fig. 3b were repeated except that cysteine substitutions at lumen-facing (**d**) or lipid-facing (**e**) positions in $^{MBP\text{-}76}$EspP(β1) were paired in all combinations with cysteine substitutions at luminal positions of ^{His}BamA(β16). Samples that were reduced prior to SDS-PAGE are shown on the lower immunoblots. Non-specific bands are denoted (*). Data are representative of at least two independent experiments.

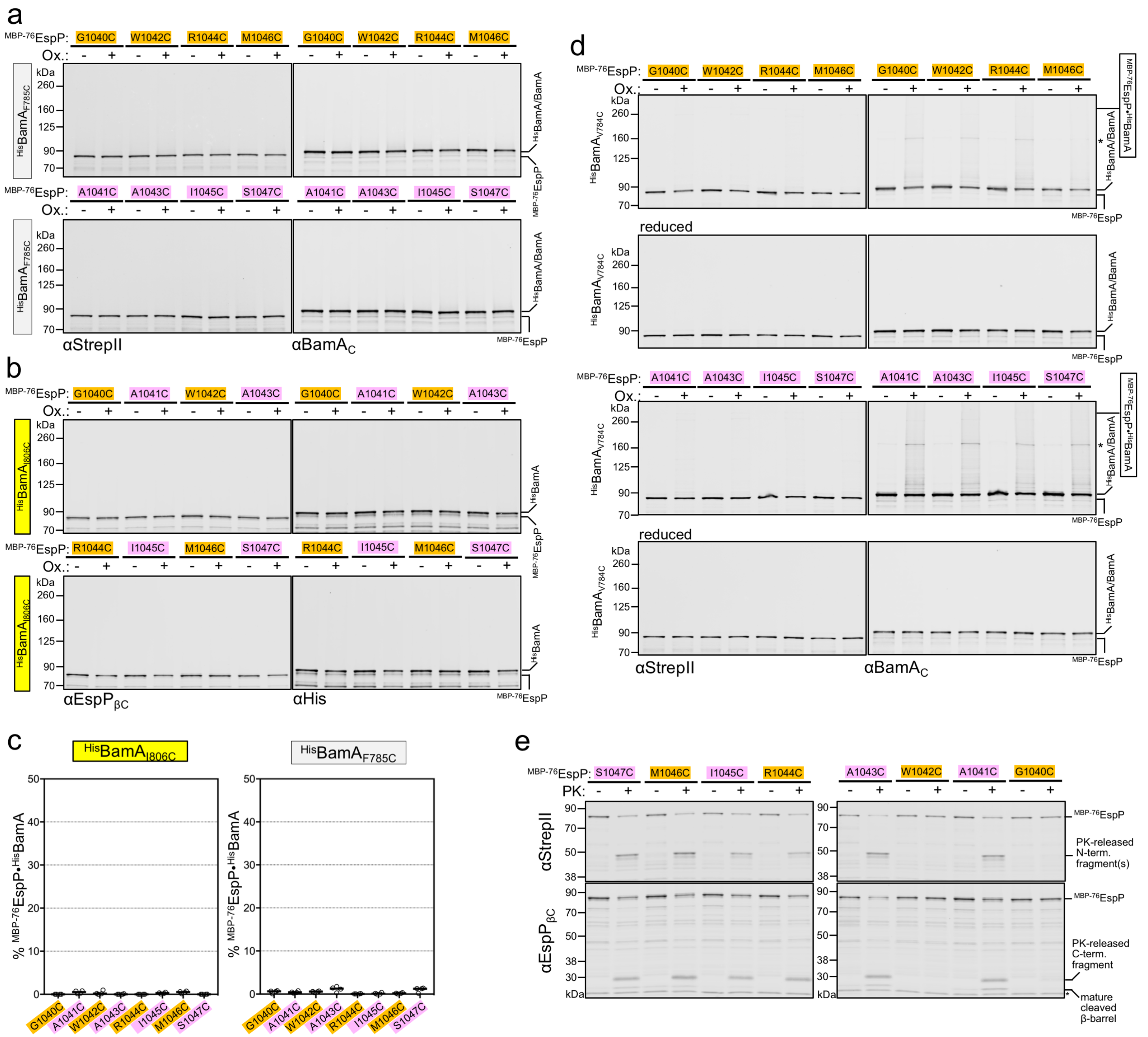

Supplementary Fig. 7 (legend next page)

Supplementary Fig. 7: Controls for disulphide bond formation assays described in Fig. 4. **a,b**, Duplex-immunoblots were performed on reduced samples from cysteine-pairing assays in Fig. 4b (**a**) and Fig. 4d (**b**) using αEspP$_{βC}$/αHis and αStrepII/αBamA$_C$ antibodies/antisera. **c**, Quantitation of ('spontaneous') disulphide-bond formation between $^{MBP\text{-}76}$EspP(β1) and ^{His}Bam$_{I806C}$ or ^{His}BamA$_{F785C}$ in cells that were mock-treated in experiments shown in Fig. 4c or Fig. 4e. Only αStrepII was used for probing immunoblots. Bars = median, N= 4. **d,** BL21(DE3) that expressed single cysteine substitutions throughout the transmembrane region of $^{MBP\text{-}76}$EspP(β1) and ^{His}BamABCDE with cysteine substitutions at lumen-facing ^{His}BamA$_{V784C}$ were mock-treated (-) or treated with 4-DPS (+). Duplex-immunoblots were then conducted using antibodies/antisera against the N-terminus of $^{MBP\text{-}76}$EspP (αStrepII) and the C-terminus of ^{His}BamA/BamA (αBamA$_C$) to monitor disulphide-bond formation *in vivo*. Samples that were reduced prior to SDS-PAGE are shown on the indicated immunoblots. Non-specific bands are denoted (*). Data are representative of at least two independent experiments. **e,** The expression of ^{His}BamA$_{I806C}$ and a $^{MBP\text{-}76}$EspP(β1) cysteine substitution was induced in the strains used in Figs. 4d and 4e and cells were PK-treated or mock-treated on ice. Immunoblots were then conducted using the indicated antibodies/antisera. A cross-reactive protein is denoted (*). Data are representative of at least two independent experiments.

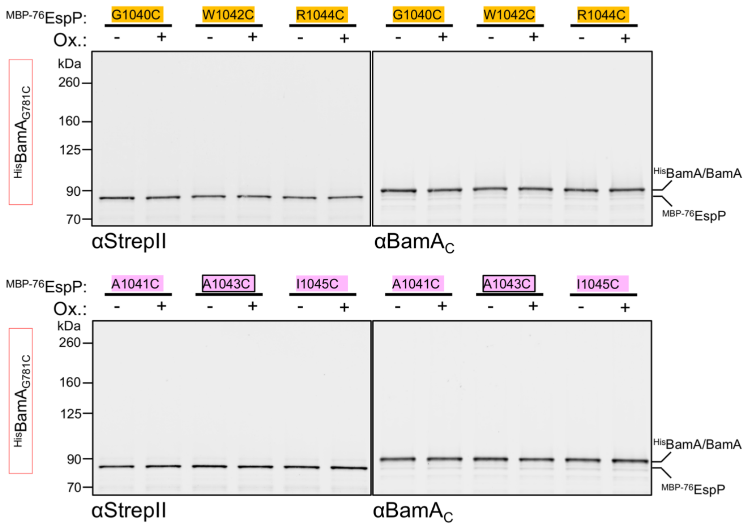

Supplementary Fig. 8: Disulphide-bond formation assay reduction controls for Fig. 5b. Duplex-immunoblots were conducted on reduced samples from cysteine-pairing assays in Fig. 5b using antibodies/antisera against the N-terminus of $^{MBP\text{-}76}$EspP (αStrep II) and the C-terminus of ^{His}BamA (αBamA$_C$). Data are representative of at least two independent experiments.

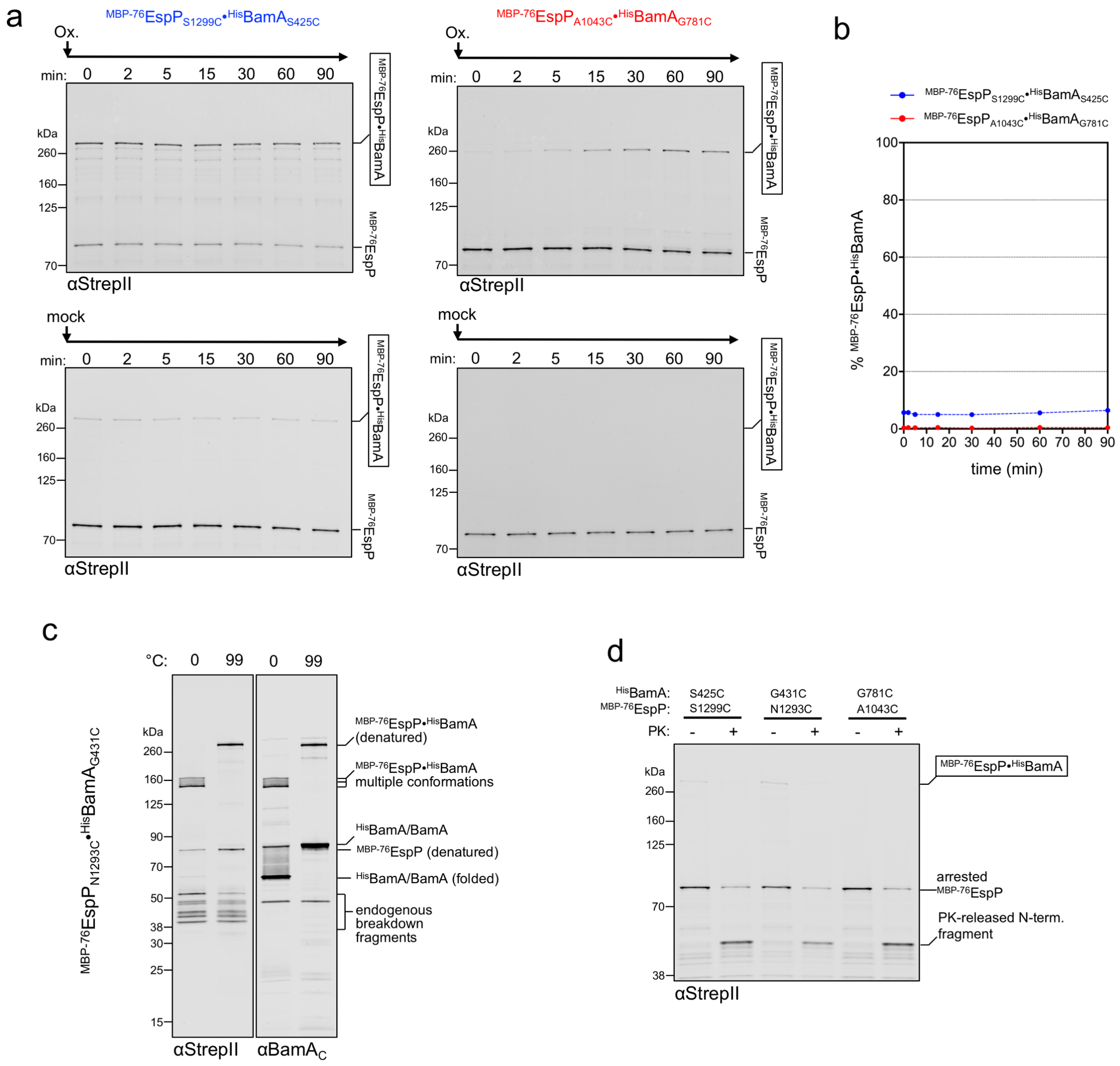

Supplementary Fig. 9: Raw data and control experiments for Fig. 6.

a, The αStrepII immunoblots from one of the replicates used to quantitate the kinetics of disulphide-bond formation in Fig. 6b are shown. Cells were treated with 4-DPS (Ox.) for up to 90 min on ice. **b,** Kinetics of spontaneous disulphide-bond formation in mock-treated cells in Fig. 6b. Points = mean ± standard error, N = 3. **c,** The experiment shown in Fig. 6c was repeated except that cells expressed the $^{MBP\text{-}76}$EspP$_{N1293C}$/^{His}BamA$_{G431C}$ pair. Duplex-immunoblots were conducted using αStrepII/αBamA$_C$ antibodies/antisera. Data are representative of at least two independent experiments. **d,** The expression of the indicated ^{His}BamA and $^{MBP\text{-}76}$EspP(β1) cysteine substitutions was induced in the strains used in experiments shown in Figs. 6b and 6c and cells were PK-treated or mock-treated on ice. Immunoblots were then conducted using αStrepII. Data are representative of at least two independent experiments.

Spontaneous oxidation						
	ANOVA					
	SS	df	MS	F	P	Sig. ?
Treatment	282	5	56.41	66.23	<0.0001	****
Residual	15.33	18	0.8517			
	Tukey's multiple comparisons					
	Adjusted P	Sig. ?				
**S425C**-S1299C vs. **N427C**-R1297C	0.9998					
**S425C**-S1299C vs. **G429C**-N1295C	>0.9999					
**S425C**-S1299C vs. **G431C**-N1293C	<0.0001	****				
**S425C**-S1299C vs. **N427C**-Y1298C	<0.0001	****				
**S425C**-S1299C vs. **S425C**-Y1298C	<0.0001	****				
**N427C**-R1297C vs. **G429C**-N1295C	0.9997					
**N427C**-R1297C vs. **G431C**-N1293C	<0.0001	****				
**N427C**-R1297C vs. **N427C**-Y1298C	<0.0001	****				
**N427C**-R1297C vs. **S425C**-Y1298C	<0.0001	****				
**G429C**-N1295C vs. **G431C**-N1293C	<0.0001	****				
**G429C**-N1295C vs. **N427C**-Y1298C	<0.0001	****				
**G429C**-N1295C vs. **S425C**-Y1298C	<0.0001	****				
**G431C**-N1293C vs. **N427C**-Y1298C	<0.0001	****				
**G431C**-N1293C vs. **S425C**-Y1298C	<0.0001	****				
**N427C**-Y1298C vs. **S425C**-Y1298C	>0.9999					
Catalysed oxidation						
	ANOVA					
	SS	df	MS	F	P	Sig. ?
Treatment	25833	5	5167	952.9	<0.0001	****
Residual	97.6	18	5.422			
	Tukey's multiple comparisons					
	Adjusted P	Sig. ?				
**S425C**-S1299C vs. **N427C**-R1297C	0.0852					
**S425C**-S1299C vs. **G429C**-N1295C	0.3225					
**S425C**-S1299C vs. **G431C**-N1293C	0.2121					
**S425C**-S1299C vs. **N427C**-Y1298C	<0.0001	****				
**S425C**-S1299C vs. **S425C**-Y1298C	<0.0001	****				
**N427C**-R1297C vs. **G429C**-N1295C	0.0011	**				
**N427C**-R1297C vs. **G431C**-N1293C	0.9949					
**N427C**-R1297C vs. **N427C**-Y1298C	<0.0001	****				
**N427C**-R1297C vs. **S425C**-Y1298C	<0.0001	****				
**G429C**-N1295C vs. **G431C**-N1293C	0.0032	**				
**G429C**-N1295C vs. **N427C**-Y1298C	<0.0001	****				
**G429C**-N1295C vs. **S425C**-Y1298C	<0.0001	****				
**G431C**-N1293C vs. **N427C**-Y1298C	<0.0001	****				
**G431C**-N1293C vs. **S425C**-Y1298C	<0.0001	****				
**N427C**-Y1298C vs. **S425C**-Y1298C	<0.0001	****				

Supplementary Table 1: ANOVA and multiple comparisons tests for disulphide-bond formation assays shown in Fig. 2e.

SS = sum of squares, df = degrees of freedom, MS = mean square. $^{MBP\text{-}76}$EspP cysteine substitutions are in regular font and ^{His}BamA substitutions are in bold font.

	ANOVA					
	SS	df	MS	F	P	Sig. ?
Treatment	28.49	2	14.24	6.34	P=0.0010	**
Residual	7.844	9	0.8716			
	Tukey's multiple comparisons					
	Adjusted P	Sig. ?				
**N805C**-R1044C vs. **G807C**-W1042C	0.3023					
**N805C**-R1044C vs. **T809C**-G1040C	0.0009	***				
**G807C**-W1042 vs. **T809C**-G1040C	0.0082	**				

Supplementary Table 2: ANOVA and multiple comparisons tests for disulphide-bond formation assays shown in Fig. 3c.

SS = sum of squares, df = degrees of freedom, MS = mean square. $^{MBP\text{-}76}$EspP cysteine substitutions are in regular font and ^{His}BamA cysteine substitutions are in bold font.

	ANOVA					
	SS	df	MS	F	P	Sig. ?
Treatment	795.3	7	113.6	12.17	<0.0001	****
Residual	224	24	9.332			
	Tukey's multiple comparisons					
	Adjusted P	Sig. ?				
**F785C**-G1040C vs. **F785C**-A1041C	>0.9999					
**F785C**-G1040C vs. **F785C**-W1042C	0.848					
**F785C**-G1040C vs. **F785C**-A1043C	0.0115	*				
**F785C**-G1040C vs. **F785C**-R1044C	0.0283	*				
**F785C**-G1040C vs. **F785C**-I1045C	0.0518					
**F785C**-G1040C vs. **F785C**-M1046C	0.9639					
**F785C**-G1040C vs. **F785C**-S1047C	0.8744					
**F785C**-A1041C vs. **F785C**-W1042C	0.9696					
**F785C**-A1041C vs. **F785C**-A1043C	0.0288	*				
**F785C**-A1041C vs. **F785C**-R1044C	0.0676					
**F785C**-A1041C vs. **F785C**-I1045C	0.0213	*				
**F785C**-A1041C vs. **F785C**-M1046C	0.8328					
**F785C**-A1041C vs. **F785C**-S1047C	0.6638					
**F785C**-W1042C vs. **F785C**-A1043C	0.2188					
**F785C**-W1042C vs. **F785C**-R1044C	0.4037					
**F785C**-W1042C vs. **F785C**-I1045C	0.002	**				
**F785C**-W1042C vs. **F785C**-M1046C	0.2693					
**F785C**-W1042C vs. **F785C**-S1047C	0.1583					
**F785C**-A1043C vs. **F785C**-R1044C	>0.9999					
**F785C**-A1043C vs. **F785C**-I1045C	<0.0001	****				
**F785C**-A1043C vs. **F785C**-M1046C	0.0009	***				
**F785C**-A1043C vs. **F785C**-S1047C	0.0004	***				
**F785C**-R1044C vs. **F785C**-I1045C	<0.0001	****				
**F785C**-R1044C vs. **F785C**-M1046C	0.0025	**				
**F785C**-R1044C vs. **F785C**-S1047C	0.0012	**				
**F785C**-I1045C vs. **F785C**-M1046C	0.3537					
**F785C**-I1045C vs. **F785C**-S1047C	0.5291					
**F785C**-M1046C vs. **F785C**-S1047C	>0.9999					

Supplementary Table 3: ANOVA and multiple comparisons tests for disulphide-bond formation assays shown in Fig. 4c.

SS = sum of squares, df = degrees of freedom, MS = mean square. $^{MBP\text{-}76}$EspP cysteine substitutions are in regular font and ^{His}BamA cysteine substitutions are in bold font.

	ANOVA					
	SS	df	MS	F	P	Sig. ?
Treatment	761.5	7	108.8	14.59	P<0.0001	****
Residual	178.9	24	7.455			
	Tukey's multiple comparisons					
	Adjusted P	Sig. ?				
**I806C**-G1040C vs. **I806C**-A1041C	0.9913					
**I806C**-G1040C vs. **I806C**-W1042C	<0.0001	****				
**I806C**-G1040C vs. **I806C**-A1043C	0.995					
**I806C**-G1040C vs. **I806C**-R1044C	0.9998					
**I806C**-G1040C vs. **I806C**-I1045C	0.73					
**I806C**-G1040C vs. **I806C**-M1046C	0.9209					
**I806C**-G1040C vs. **I806C**-S1047C	0.8576					
**I806C**-A1041C vs. **I806C**-W1042C	<0.0001	****				
**I806C**-A1041C vs. **I806C**-A1043C	>0.9999					
**I806C**-A1041C vs. **I806C**-R1044C	0.9077					
**I806C**-A1041C vs. **I806C**-I1045C	0.2719					
**I806C**-A1041C vs. **I806C**-M1046C	0.9999					
**I806C**-A1041C vs. **I806C**-S1047C	0.9989					
**I806C**-W1042C vs. **I806C**-A1043C	<0.0001	****				
**I806C**-W1042C vs. **I806C**-R1044C	<0.0001	****				
**I806C**-W1042C vs. **I806C**-I1045C	0.0006	***				
**I806C**-W1042C vs. **I806C**-M1046C	<0.0001	****				
**I806C**-W1042C vs. **I806C**-S1047C	<0.0001	****				
**I806C**-A1043C vs. **I806C**-R1044C	0.9298					
**I806C**-A1043C vs. **I806C**-I1045C	0.304					
**I806C**-A1043C vs. **I806C**-M1046C	0.9997					
**I806C**-A1043C vs. **I806C**-S1047C	0.9977					
**I806C**-R1044C vs. **I806C**-I1045C	0.9301					
**I806C**-R1044C vs. **I806C**-M1046C	0.7119					
**I806C**-R1044C vs. **I806C**-S1047C	0.6089					
**I806C**-I1045C vs. **I806C**-M1046C	0.1318					
**I806C**-I1045C vs. **I806C**-S1047C	0.0945					
**I806C**-M1046C vs. **I806C**-S1047C	>0.9999					

Supplementary Table 4: ANOVA and multiple comparisons tests for disulphide-bond formation assays shown in Fig. 4e.

SS = sum of squares, df = degrees of freedom, MS = mean square. $^{\text{MBP-76}}$EspP cysteine substitutions are in regular font and ^{His}BamA cysteine substitutions are in bold font.

	ANOVA					
	SS	df	MS	F	P	Sig. ?
Treatment	3163	5	632.6	63.4	P<0.0001	****
Residual	179.6	18	9.977			
	Tukey's multiple comparisons					
	Adjusted P	Sig. ?				
**G781C**-G1040C vs. **G781C**-A1041C	0.9437					
**G781C**-G1040C vs. **G781C**-W1042C	0.9707					
**G781C**-G1040C vs. **G781C**-A1043C	<0.0001	****				
**G781C**-G1040C vs. **G781C**-R1044C	0.0188	*				
**G781C**-G1040C vs. **G781C**-I1045C	>0.9999					
**G781C**-A1041C vs. **G781C**-W1042C	>0.9999					
**G781C**-A1041C vs. **G781C**-A1043C	<0.0001	****				
**G781C**-A1041C vs. **G781C**-R1044C	0.0029	**				
**G781C**-A1041C vs. **G781C**-I1045C	0.9742					
**G781C**-W1042C vs. **G781C**-A1043C	<0.0001	****				
**G781C**-W1042C vs. **G781C**-R1044C	0.0038	**				
**G781C**-W1042C vs. **G781C**-I1045C	0.989					
**G781C**-A1043C vs. **G781C**-R1044C	<0.0001	****				
**G781C**-A1043C vs. **G781C**-I1045C	<0.0001	****				
**G781C**-R1044C vs. **G781C**-I1045C	0.0137	*				

Supplementary Table 5: ANOVA and multiple comparisons tests for disulphide-bond formation assays shown in Fig. 5.

SS = sum of squares, df = degrees of freedom, MS = mean square. $^{\text{MBP-76}}$EspP cysteine substitutions are in regular font and ^{His}BamA cysteine substitutions are in bold font.

Plasmid	Alias/derivative	Notes	Source
pMAL-p2x		MBP-fusion cloning and expression vector	NEB
pJH64		pTrc99a::*espP(C)*$_{SP}$*-espP909-1300* (EspPΔ1), P_{Trc}, Amp^{R}	1
pWK1		pSCRhaB2-RDEspPΔ1, P_{rhaB}, Trim^{R}	2
pJH114		pTrc99a::*bamABCDE*^{His8}	3
pJH64::*malE*		pTrc99a::*espP(C)*$_{SP}$*-malE-espP909-1300*	This work
pRha-MBP-EspPΔ1		pSCrhaB2::*espP(C)*$_{SP}$*-malE-espP909-1300*	This work
pMTD321	pRha-MBP-EspPΔ1::mtd95	pSCrhaB2::*espP(C)*$_{SP}$*-TS-malE-espP909-1300* (115aa-linker) (gBlock mtd95 for TwinStrep inserted via GA at N-term of *malE-espPΔ1*)	This work
pMTD366	pJH114::mtd101	pTrc99a::^{His8}*bamABCDE*^{His8} (gBlock mtd101 inserted His8-GG via GA at N-term BamA POTRA via GA)	This work
pMTD372	pMTD366(mtd104/105)	pTrc99a::^{His8}*bamABCDE* (*bamE* native stop codon repaired via QC with primers mtd104/105)	This work
pMTD501	pMTD321(mtd145/146)	pSCrhaB2::*espP(C)*$_{SP}$*-TS-malE-espP927-1300* (97aa-linker) (*espP909-926* excised via primers mtd145/146 and BamHI)	This work
pMTD502	pMTD321(mtd145/147)	pSCrhaB2::*espP(C)*$_{SP}$*-TS-malE-espP948-1300* (76aa-linker) (*espP909-947* excised via primers mtd145/147 and BamHI*)*	This work
pMTD521	pMTD502::mtd137	pSCrhaB2::*espP(C)*$_{SP}$*-TS-malE-espP948-974-tev-espP975-1300* (gBlock mtd137 for Gly-TEV-Gly site inserted via GA after EspP974 via GA)	This work
pMTD607	pMTD502::mtd159	pSCrhaB2::*espP(C)*$_{SP}$*-TS-malE-espP948-984-tev-espP985-1300* (pRha^{MBP-76}EspP) (gBlock mtd159 for Gly-TEV-Gly site inserted via GA after EspP984 via GA)	This work
pMTD610	pMTD321::mtd145/164	pSCrhaB2::*espP(C)*$_{SP}$*-TS-malE-espP965-1300* (59aa-linker) (*espP909-964* excised via primers mtd145/164 and BamHI*)*	This work
pMTD710	pMTD372(mtd188/189)	pTrc99a::^{His8}*bamA*$_{S425C}$*-BCDE* (*bamA*$_{S425C}$ substitution via QC with mtd188/189 primers)	This work
pMTD712	pMTD607(mtd190/191)	pRha^{MBP-76}EspP$_{S1299C}$ (*espP*$_{S1299C}$ substitution via QC with mtd190/191 primers)	This work
pMTD725	pMTD372(mtd201/202)	pTrc99a::^{His8}*bamA*$_{C690S}$*-BCDE* (*bamA*$_{C690S}$ substitution via QC with mtd201/202 primers)	This work
pMTD744	pMTD725(mtd203/204)	pTrc99a::^{His8}*bamA*$_{C690S/C700S}$*-BCDE* (*bamA*$_{C700S}$ substitution via QC with mtd203/204 primers)	This work
pMTD754	pMTD744(mtd188/189)	pTrc99a::^{His8}*bamA*$_{C690S/C700S/S425C}$*-BCDE* (*bamA*$_{S425C}$ substitution via QC with mtd188/189 primers)	This work
pMTD792	pMTD607(mtd219/220)	pRha^{MBP-76}EspP$_{R1297C}$ (*espP*$_{R1297C}$ substitution via QC with mtd219/220 primers)	This work
pMTD798	pMTD607(mtd186/187)	BamHI introduced between TS and *malE* via QC with mtd186/187 primers	This work
pMTD800	pMTD607(mtd217/218)	pRha^{MBP-76}EspP$_{Y1298C}$ (*espP*$_{Y1298C}$ substitution via QC with mtd217/218 primers)	This work
pMTD814	pMTD607(mtd242/243)	pRha^{MBP-76}EspP$_{G1040C}$ (*espP*$_{G1040C}$ substitution via QC with mtd242/243 primers)	This work
pMTD816	pMTD607(mtd244/245)	pRha^{MBP-76}EspP$_{W1042C}$ (*espP*$_{W1042C}$ substitution via QC with mtd244/245 primers)	This work
pMTD818	pMTD372(mtd246/247)	pTrc99a::^{His8}*bamA*$_{G807C}$*-BCDE* (*bamA*$_{G807C}$ substitution via QC with mtd246/247 primers)	This work
pMTD820	pMTD372(mtd248/249)	pTrc99a::^{His8}*bamA*$_{T809C}$*-BCDE* (*bamA*$_{T809C}$ substitution via QC with mtd248/249 primers)	This work
pMTD826	pMTD798Δ*malE*	pSCrhaB2::*espP(C)*$_{SP}$*-TS-espP948-984-tev-espP985-1300* (^{76}EspP) (*malE* excised via BamHI)	This work
pMTD829	pMTD372(mtd205/206)	pTrc99a::^{His8}*bamA*$_{N427C}$*-BCDE* (*bamA*$_{N427C}$ substitution via QC with mtd205/206 primers)	This work
pMTD889	pMTD372(mtd253/254)	pTrc99a::^{His8}*bamA*$_{G429C}$*-BCDE* (*bamA*$_{G429C}$ substitution via QC with mtd253/254 primers)	This work
pMTD891	pMTD372(mtd255/256)	pTrc99a::^{His8}*bamA*$_{G431C}$*-BCDE* (*bamA*$_{G431C}$ substitution via QC with mtd255/256 primers)	This work
pMTD893	pMTD372(mtd257/258)	pTrc99a::^{His8}*bamA*$_{G781C}$*-BCDE* (*bamA*$_{G781C}$ substitution via QC with mtd257/258 primers)	This work
pMTD896	pMTD372(mtd261/262)	pTrc99a::^{His8}*bamA*$_{N805C}$*-BCDE* (*bamA*$_{N805C}$ substitution via QC with mtd261/262 primers)	This work
pMTD918	pMTD607(mtd265/266)	pRha^{MBP-76}EspP$_{R1044C}$ (*espP*$_{R1044C}$ substitution via QC with mtd265/266 primers)	This work
pMTD920	pMTD607(mtd267/268)	pRha^{MBP-76}EspP$_{N1295C}$ (*espP*$_{N1295C}$ substitution via QC with mtd267/268 primers)	This work
pMTD922	pMTD607(mtd269/270)	pRha^{MBP-76}EspP$_{N1293C}$ (*espP*$_{N1293C}$ substitution via QC with mtd269/270 primers)	This work
pMTD949	pMTD607(mtd271/272)	pRha^{MBP-76}EspP$_{A1041C}$ (*espP*$_{A1041C}$ substitution via QC with mtd271/272 primers)	This work
pMTD951	pMTD607(mtd273/274)	pRha^{MBP-76}EspP$_{A1043C}$ (*espP*$_{A1043C}$ substitution via QC with mtd273/274 primers)	This work
pMTD957	pMTD372(mtd275/276)	pTrc99a::^{His8}*bamA*$_{I806C}$*-BCDE* (*bamA*$_{I806C}$ substitution via QC with mtd275/276 primers)	This work
pMTD1024	pMTD372(mtd281/282)	pTrc99a::^{His8}*bamA*$_{V784C}$*-BCDE* (*bamA*$_{V784C}$ substitution via QC with mtd281/282 primers)	This work
pMTD1026	pMTD372(mtd283/284)	pTrc99a::^{His8}*bamA*$_{F785C}$*-BCDE* (*bamA*$_{F785C}$ substitution via QC with mtd283/284 primers)	This work
pMTD1029	pMTD372(mtd207/208)	pTrc99a::^{His8}*bamA*$_{G443C}$*-BCDE* (*bamA*$_{G443C}$ substitution via QC with mtd207/208 primers)	This work
pMTD1062	pMTD607(mtd279/280)	pRha^{MBP-76}EspP$_{I1045C}$ (*espP*$_{I1045C}$ substitution via QC with mtd279/280 primers)	This work
pMTD1086	pMTD607(mtd289/290)	pRha^{MBP-76}EspP$_{M1046C}$ (*espP*$_{M1046C}$ substitution via QC with mtd289/290 primers)	This work
pMTD1089	pMTD607(mtd291/292)	pRha^{MBP-76}EspP$_{S1047C}$ (*espP*$_{S1047C}$ substitution via QC with mtd291/292 primers)	This work

Supplementary Table 6 (legend next page)

Supplementary Table 6: Plasmids used in this study.
Plasmid numbering is hierarchical with plasmid number derived from the cloning/storage strain number. Abbreviations: (C)SP = C-terminal signal peptide sequence, *malE* = , *malE26-392* codons, MBP = Maltose/Maltodextrin binding protein (26-392), Amp^R = Ampicillin resistance, $Trim^R$ = Trimethoprim resistance, TS = TwinStrepII tag, His/His8 = His x 8 tag, QC = QuikChange site directed mutagenesis, GA = Gibson Assembly.

(1) Szabady, R. L., Peterson, J. H., Skillman, K. M. & Bernstein, H. D. An unusual signal peptide facilitates late steps in the biogenesis of a bacterial autotransporter. Proc Natl Acad Sci U S A 102, 221-226, (2005).
(2) Kang'ethe, W. & Bernstein, H. D. Charge-dependent secretion of an intrinsically disordered protein via the autotransporter pathway. Proc Natl Acad Sci U S A 110, E4246-4255, (2013).
(3) Roman-Hernandez, G., Peterson, J. H. & Bernstein, H. D. Reconstitution of bacterial autotransporter assembly using purified components. eLife 3, e04234, (2014).

Supplementary Table 7: Oligonucleotides and double-stranded DNA fragments used in this study.

DNA	Sequence	Notes
**ssDNA oligos**		
mtd1	TAATCATCCGGCTCGTATAATGTG	F, sequencing primer, pTrc99a
mtd20	GGCATGGGGTCAGGTGG	R, sequencing primer, pTrc99a
mtd42	CATCACGTTCATCTTTCCCTGG	F, sequencing primer, pSCrhaB2
mtd43	CGGCGCTACGGCGTTTCAC	R, sequencing primer, pSCrhaB2
mtd96	GGCCGCAAAAGAATATGAGG	R, primer to linearize pMTD294 for TwinStrepII insertion (mtd95) between *espP(C)*$_{SP}$ and *malE* via GA
mtd97	GGCCGCAAAAGAATATGAGG	F, primer to linearize pMTD294 for TwinStrepII insertion (mtd95) between *espP(C)*$_{SP}$ and *malE* via GA
mtd100	GCTGAGCGCAGGTATTAACGC	F, sequencing primer, *malE* middle
mtd102	AGCACCGTATACGGTGGCG	R, primer to linearize pJH114 at *bamA* sequence at +1 SP cleavage site for His-tag insertion (mtd101) via GA
mtd103	GAAGGGTTCGTAGTGAAAGATATTC	F, primer to linearize pJH114 at *bamA* sequence at +1 SP cleavage site for His-tag insertion (mtd101) via GA
mtd104	CAAACCTGCGCTGAGTGGTAACtaaGGTCATCATCACCACC	F, QC primer to put stop codon at end of *bamE-His* (to remove His-tag)
mtd105	GGTGGTGATGATGACCttaGTTACCACTCAGCGCAGGTTTG	F, QC primer to put stop codon at end of *bamE-His* (to remove His-tag)
mtd110	ACCCAGGTCAGTCTGACGCCAGAT	F, sequencing primer, *bamA* second (pTrc99a::*His8bamABCDE*)
mtd111	TGGTACAGACGTGACGTTGGGCTT	F, sequencing primer, *bamA* third (pTrc99a::*His8bamABCDE*)
mtd112	CTGGTGTTCCAGGAAGGTGTGTCA	F, sequencing primer, *bamA* first (pTrc99a::*His8bamABCDE*)
mtd113	CGGGTATCGCATTACAATGGATGTC	F, sequencing primer, *bamA* into *bamB* (pTrc99a::*His8bamABCDE*)
mtd114	GGTGTTAATCCACACCAGTAACGG	F, sequencing primer, *bamB* second (pTrc99a::*His8bamABCDE*)
mtd115	CGTCGAAGATGGTCGTTTCGTTGCC	F, sequencing primer, *bamB* into *bamC* (pTrc99a::*His8bamABCDE*)
mtd116	CCCAACGTGATGATGCTGGTCAGAC	F, sequencing primer, *bamC* second (pTrc99a::*His8bamABCDE*)
mtd117	TGACGCGCATGAAATATCTGGTGGC	F, sequencing primer, *bamD* first (pTrc99a::*His8bamABCDE*)
mtd118	TACTCCGTGGCCGAGTACTATACAG	F, sequencing primer, *bamD* into *bamE* (pTrc99a::*His8bamABCDE*)
mtd119	GATGAGATCCAGCTGCACCTGCTGC	R, sequencing primer, *bamD* backwards into *bamC* (pTrc99a::*His8bamABCDE*)
mtd120	CGCATCAGGCCAGTAATTATGATC	F, sequencing primer, *bamA* fourth (pTrc99a::*His8bamABCDE*)
mtd135	ATCACTGAAACCAATGGTTTGTTTACTG	R, primer to linearize pMTD502 between *espP* codons 974 and 975 for G-TEV-G insertion (mtd137) via GA
mtd136	GTAACGCCGGTCATTACAACCAGG	F, primer to linearize pMTD502 between *espP* codons 974 and 975 for G-TEV-G insertion (mtd137) via GA
mtd143	GTGCGTACTGCGGTGATCAAC	F, sequencing primer, *malE* C-terminus coding
mtd145	TTTTTTggatccCGAATTAGTCTGCGCGTCTTTC	R, to linearize pMTD321 from 3' end of *malE* plus BamHI restriction site
mtd146	TTTTTTggatccTCTGGTGAGAACAATATTTTACTCGTTG	F, to linearize pMTD321 from *espP* codon 927 plus BamHI restriction site
mtd147	TTTTTTggatccAATATTGAACTGGTAAGCGCGCC	F, to linearize pMTD321 from *espP* codon 948 plus BamHI restriction site
mtd160	GGTTTCCCTGGTTGTAATGACCG	R, primer to linearize pMTD502 between *espP* codons 984 and 985 for G-TEV-G insertion (mtd159) via GA
mtd161	GATGACAAAATAACATGGTCACTGAC	F, primer to linearize pMTD502 between *espP* codons 984 and 985 for G-TEV-G insertion (mtd159) via GA
mtd164	TTTTTTggatccGCCAGTAAACAAACCATTGG	F, to linearize pMTD321 from *espP* codon 965 plus BamHI restriction site
mtd186	CCGCAGTTTGAAAAGGGTGGCGgatccATCGAAGAAGGTAAACTGGTAATC	F, QC primer to put BamHI between TS and *malE* in pMTD607
mtd187	GATTACCAGTTTACCTTCTTCGATggatcCGCCACCCTTTTCAAACTGCGG	R, QC primer to put BamHI between TS and *malE* in pMTD607
mtd188	GGTAAAAGAGCGCAACACCGGTtGCTTCAACTTTGGTATTGG	F, QC primer for *bamA S425C* substitution
mtd189	CCAATACCAAAGTTGAAGCaACCGGTGTTGCGCTCTTTTACC	R, QC primer for *bamA S425C* substitution
mtd190	GTCAACGCTAATTTCCGTTACTgcTTCTGATACTCCGTATTC	F, QC primer for *espP S1299C* substitution
mtd191	GAATACGGAGTATCAGAAgcAGTAACGGAAATTAGCGTTGAC	R, QC primer for *espP S1299C* substitution
mtd201	GGACTATGATTACGAATcTGCGACTCAGGACGGCG	F, QC primer for *bamA C690S* substitution

Continued next page

mtd202	CGCCGTCCTGAGTCGCAgATTCGTAATCATAGTCC	R, QC primer for *bamA C690S* substitution
mtd203	GGCGCGAAAGACCTGTcTAAATCGGATGATGCTGTAGGC	F, QC primer for *bamA C700S* substitution
mtd204	GCCTACAGCATCATCCGATTTAgACAGGTCTTTCGCGCC	R, QC primer for *bamA C700S* substitution
mtd205	CGCAACACCGGTAGCTTCtgCTTTGGTATTGGTTACGG	F, QC primer for *bamA N427C* substitution
mtd206	CCGTAACCAATACCAAAGcaGAAGCTACCGGTGTTGCG	R, QC primer for *bamA N427C* substitution
mtd207	CGTGAGCTTCCAGGCTtGTGTGCAGCAGGATAACTGG	F, QC primer for *bamA G443C* substitution
mtd208	CCAGTTATCCTGCTGCACACaAGCCTGGAAGCTCACG	R, QC primer for *bamA G443C* substitution
mtd215	GTCAACGCTAATTTCCGTTACTCGTgCTGATACTCCGTATTC	F, QC primer for *espP F1300C* substitution
mtd216	GAATACGGAGTATCAGcACGAGTAACGGAAATTAGCGTTGAC	R, QC primer for *espP F1300C* substitution
mtd217	GTCAACGCTAATTTCCGTTgCTCGTTCTGATACTCCGTATTC	F, QC primer for *espP Y1298C* substitution
mtd218	GAATACGGAGTATCAGAACGAGcAACGGAAATTAGCGTTGAC	R, QC primer for *espP Y1298C* substitution
mtd219	GTCAACGCTAATTTCtGTTACTCGTTCTGATACTCCGTATTC	F, QC primer for *espP R1297C* substitution
mtd220	GAATACGGAGTATCAGAACGAGTAACaGAAATTAGCGTTGAC	R, QC primer for *espP R1297C* substitution
mtd242	CAACGGCGAAGCCtGTGCATGGGCACGCATC	F, QC primer for *espP G1040C* substitution
mtd243	GATGCGTGCCCATGCACaGGCTTCGCCGTTG	R, QC primer for *espP G1040C* substitution
mtd244	CGAAGCCGGTGCATGcGCACGCATCATG	F, QC primer for *espP W1042C* substitution
mtd245	CATGATGCGTGCgCATGCACCGGCTTCG	R, QC primer for *espP W1042C* substitution
mtd246	CAGTTCCAGTTTAACATCtGTAAAACCTGGTAAGTGGG	F, QC primer for *bamA G807C* substitution
mtd247	CCCACTTACCAGGTTTTACaGATGTTAAACTGGAACTG	R, QC primer for *bamA G807C* substitution
mtd248	CAGTTCCAGTTTAACATCGGTAAAtgCTGGTAAGTGGGATC	F, QC primer for *bamA T809C* substitution
mtd249	GATCCCACTTACCAGcaTTTACCGATGTTAAACTGGAACTG	R, QC primer for *bamA T809C* substitution
mtd253	GCAACACCGGTAGCTTCAACTTTtGTATTGGTTACGGTAC	F, QC primer for *bamA G429C* substitution
mtd254	GTACCGTAACCAATACaAAAGTTGAAGCTACCGGTGTTGC	R, QC primer for *bamA G429C* substitution
mtd255	CCGGTAGCTTCAACTTTGGTATTtGTTACGGTACTGAAAGTGG	F, QC primer for *bamA G431C* substitution
mtd256	CCACTTTCAGTACCGTAACaAATACCAAAGTTGAAGCTACCGG	R, QC primer for *bamA G431C* substitution
mtd257	GGATGTCCCCATTGtGcCCGTTGGTGTTCTCC	F, QC primer for *bamA G781C* substitution
mtd258	GGAGAACACCAACGGgCaCAATGGGGACATCC	R, QC primer for *bamA G781C* substitution
mtd261	CAAGGCAGAACAGTTCCAGTTTtgCATCGGTAAAACCTGG	F, QC primer for *bamA N805C* substitution
mtd262	CCAGGTTTTACCGATGcaAAACTGGAACTGTTCTGCCTTG	R, QC primer for *bamA N805C* substitution
mtd265	CGAAGCCGGTGCATGGGCAtGCATCATGAGCGG	F, QC primer for *espP R1044C* substitution
mtd266	CCGCTCATGATGCaTGCCCATGCACCGGCTTCG	R, QC primer for *espP R1044C* substitution
mtd267	CGTTGATAATGCTGTCAACGCTTgTTTCCGTTACTCGTTCTG	F, QC primer for *espP N1295C* substitution
mtd268	CAGAACGAGTAACGGAAAcaAGCGTTGACAGCATTATCAACG	R, QC primer for *espP N1295C* substitution
mtd269	CGTTGATAATGCTGTCtgCGCTAATTTCCGTTACTCGTTCTG	F, QC primer for *espP N1293C* substitution
mtd270	CAGAACGAGTAACGGAAATTAGCGcaGACAGCATTATCAACG	R, QC primer for *espP N1293C* substitution
mtd271	CGGCGAAGCCGGTtgcTGGGCACGCATCATGAGCGG	F, QC primer for *espP A1041C* substitution
mtd272	CCGCTCATGATGCGTGCCCAgcaACCGGCTTCGCCG	R, QC primer for *espP A1041C* substitution
mtd273	CGGCGAAGCCGGTGCATGGtgcCGCATCATGAGCGG	F, QC primer for *espP A1043C* substitution
mtd274	CCGCTCATGATGCGgcaCCATGCACCGGCTTCGCCG	R, QC primer for *espP A1043C* substitution
mtd275	CAAGGCAGAACAGTTCCAGTTTAACtgCGGTAAAACCTGG	F, QC primer for *bamA I806C* substitution

Continued next page

mtd276	CCAGGTTTTACCGcaGTTAAACTGGAACTGTTCTGCCTTG	R, QC primer for *bamA I806C* substitution
mtd279	GCCGGTGCATGGGCACGCtgCATGAGCGGTACCGGC	F, QC primer for *espP I1045C* substitution
mtd280	GCCGGTACCGCTCATGcaGCGTGCCCATGCACCGGC	R, QC primer for *espP I1045C* substitution
mtd281	GTCCCCATTGGGGCCGTTGtgcTTCTCCTACGCCCAGCCG	F, QC primer for *bamA V784C* substitution
mtd282	CGGCTGGGCGTAGGAGAAgcaCAACGGCCCCAATGGGGAC	R, QC primer for *bamA V784C* substitution
mtd283	CCATTGGGGCCGTTGGTGTgCTCCTACGCCCAGC	F, QC primer for *bamA F785C* substitution
mtd284	GCTGGGCGTAGGAGcACACCAACGGCCCCAATGG	R, QC primer for *bamA F785C* substitution
mtd289	GCATGGGCACGCATCtgcAGCGGTACCGGCTC	F, QC primer for *espP M1046C* substitution
mtd290	GAGCCGGTACCGCTgcaGATGCGTGCCCATGC	R, QC primer for *espP M1046C* substitution
mtd291	GCATGGGCACGCATCATGtGCGGTACCGGCTCTG	F, QC primer for *espP S1047C* substitution
mtd292	CAGAGCCGGTACCGCaCATGATGCGTGCCCATGC	R, QC primer for *espP S1047C* substitution
pTRC 399 R	TTATCAGACCGCTTCTGCGTTCTG	R, pTrc sequencing primer 2
Mal.Eag(+)	CGATGATGTTTTCCGCCTCGGCGGCCGCCAAAATC	F, primer for subcloning *malE* from pMAL-p2x
Mal.Eag(-)	GTTGTTGTTGTTCGCGGCCGAATTAGTCTGCGCG	R, primer for subcloning *malE* from pMAL-p2x
**Linear dsDNA fragments**		
mtd95	CTTGCATTATGTTTTTTAGGCTTATTACAATCCTCATATTCTTTTGCGGCCT<br>GGTCTCATCCGCAGTTTGAAAAGGGTGGCGGAGCGGTGGCGGTAGCGGTGG<br>CTCCGCGTGGAGCCATCCGCAGTTTGAAAAGGGTGGCGCCAAAATCGAAGAA<br>GGTAAACTGGTAATCTGGATTAACGGCGATAAAGGC	Fragment containing TwinStrepII-tag to assemble with pMTD294
mtd101	AGTTGCTCATAGCGTCGCTGCTGTTTAGCAGCGCCACCGTATACGGTGCTCA<br>TCACCATCACCATCACCATCACGGTGGCGAAGGGTTCGTAGTGAAAGATATT<br>CATTTCGAAGGCCTTCAGCGTGTCGC	Fragment containing His8-Gly2-tag to assemble with pJH114
mtd137	CCAATGAAAATGTCTTTAAAGCCAGTAAACAAACCATTGGTTTCAGTGATGG<br>CGAAAACCTGTATTTTCAGGGCGGCGTAACGCCGGTCATTACAACCAGGGAA<br>ACCGATGACAAAATAACATGGTC	Fragment containing TEV cleavage site to assemble with pMTD502 (between *espP974/975*)
mtd159	CCATTGGTTTCAGTGATGTAACGCCGGTCATTACAACCAGGGAAACCGGCGA<br>AAACCTGTATTTTCAGGGCGGCGATGACAAAATAACATGGTCACTGACAGGC<br>TATAACACGGTAGCAAACAAGG	Fragment containing TEV cleavage site to assemble with pMTD502 (between *espP984/985*)

Supplementary Table 7: Oligonucleotides and double-stranded DNA fragments used in this study.
F = forward strand, R = reverse strand, SP = signal peptide, GA = Gibson assembly, QC = QuikChange
