# Supplementary material for: Bacterial outer membrane proteins assemble via asymmetric interactions with the BamA β-barrel
Source: Nat Commun. 2019 Jul 26;10:3358. doi: 10.1038/s41467-019-11230-9 (PMC6659671; doi:10.1038/s41467-019-11230-9)
Supplement: Supplementary file 3 — Description of Additional Supplementary Files [file 41467_2019_11230_MOESM3_ESM.docx]

**Title:** Supplementary Movie 1
**Description:** OMP assembly by BamA via asymmetric hybrid-barrel intermediates. An aminated version of the model depicted in Fig. 7 is shown.
