# Supplementary material for: Bacterial outer membrane proteins assemble via asymmetric interactions with the BamA β-barrel
Source: Nat Commun. 2019 Jul 26;10:3358. doi: 10.1038/s41467-019-11230-9 (PMC6659671; doi:10.1038/s41467-019-11230-9)
Supplement: Supplementary file 5 — Source Data [file 41467_2019_11230_MOESM5_ESM.zip › 202991_2_related_ms_3899545_ptpnrr.pdf]

Fig. 1e

	% mature beta-barrel	
time (min)	PK- (mock)	PK+
0.5	0.1227965	7.239044
2	0.1981483	84.923039
10	0.1536407	90.050109
30	0.7385194	93.551264

Fig. 2e

Spontaneous

% $^{MBP-76}$EspP-^{His}BamA					
S425C S1299C	N427C R1297C	G429C N1295C	G431C N1293C	N427C Y1298C	S425C Y1298C
4.8300472	6.011379364	5.424215255	7.13413024	0	0
5.572155651	4.664270991	5.739197157	10.59934058	0	0
4.843457229	5.523320839	4.062119638	10.25990339	0	0
5.545790174	5.295402917	5.503154443	11.81439605	0.543073999	0

Catalysed

% $^{MBP-76}$EspP-^{His}BamA					
S425C S1299C	N427C R1297C	G429C N1295C	G431C N1293C	N427C Y1298C	S425C Y1298C
83.58332493	83.94802483	80.23296739	84.60393246	5.839048857	23.68135397
82.46665021	87.84196607	80.23002675	87.5921715	4.364774495	27.74647385
83.01515834	88.68400492	80.4970445	89.47120414	1.953110331	28.68228366
82.56545739	90.27071993	76.73157199	85.68697224	6.101534187	32.66311035

Fig. 3c

% $^{MBP-76}$EspP-^{His}BamA		
N805C R1044C	G807C W1042C	T809C G1040C
2.09001517	2.34816036	3.29957266
0.9082681	2.38272101	4.05127191
0.99075759	1.78595064	5.87412428
0.99219735	2.63777272	6.40687502

Fig. 4c

% $^{MBP-76}$EspP-^{His}BamA							
F785C G1040C	F785C A1041C	F785C W1042C	F785C A1043C	F785C R1044C	F785C I1045C	F785C M1046C	F785C S1047C
12.17105032	12.93545681	8.700758181	4.529768733	5.914492401	12.85838519	17.77658213	19.29889196
12.91466873	14.61069437	10.05862614	5.362672825	4.798227824	21.57586158	15.11303774	15.23961119
12.69313817	11.00936403	8.240279034	4.377916062	5.64810651	18.26501753	18.05243202	17.60282038
14.49683605	10.27536248	13.16952045	3.776534369	5.076515789	28.05357233	10.27881593	11.71147655

Fig. 4e

% $^{MBP-76}$EspP-^{His}BamA							
I806C G1040C	I806C A1041C	I806C W1042C	I806C A1043C	I806C R1044C	I806C I1045C	I806C M1046C	I806C S1047C
4.01372755	4.06311171	15.6074133	4.63866317	7.09817102	9.0304811	2.55303795	2.74186274
4.76216423	3.76950584	17.5822756	4.4778723	4.80649925	5.51933203	2.84384613	3.29810764
3.27779407	3.55416737	15.9762604	4.54502158	3.282233	6.2853451	2.75346668	2.99688623
10.2259606	4.6919146	25.7866341	2.95835267	10.5526704	14.0139549	4.78086934	2.58949227

Fig. 5c

Spontaneous

% $^{MBP-76}$EspP-^{His}BamA					
G781C G1040C	G781C A1041C	G781C W1042C	G781C A1043C	G781C R1044C	G781C I1045C
0.880915187	0	0.708269477	1.370256132	0.44604081	0.051672909
1.045941749	0	0.573396387	0.349156139	0.468280042	0.209128147
1.104455698	0	0.85978148	0.209624257	0.375597973	0.123857492
0.185550539	0	0.424131858	0.099917826	0.108200622	0.167617344

Catalysed

% $^{MBP-76}$EspP-^{His}BamA					
G781C G1040C	G781C A1041C	G781C W1042C	G781C A1043C	G781C R1044C	G781C I1045C
13.61917674	14.95962082	15.11947342	31.71549628	5.075906282	16.41384824
12.26773437	13.33783788	12.76537111	44.53384875	3.674412235	11.70926879
11.2051381	12.88508485	14.32967065	43.45012279	2.959709358	11.98041539
8.782831082	12.65530008	10.46221506	40.46803001	1.466592672	7.135457256

Fig. 6b

	% $^{MBP-76}$EspP-^{His}BamA					
Time (min)	S425C S1299C			G781C A1043C		
0	64.9356893	53.5093836	58.060185	1.0890497	1.225183	1.3769786
2	62.8366348	62.3070573	63.1628502	2.9316826	3.2284058	1.9184291
5	65.4800265	59.9873742	61.2458729	4.3858139	6.7446093	4.7676913
15	70.5605258	60.3740379	59.487475	13.0687084	14.2236628	11.7078262
30	71.9055449	66.172902	61.2309632	23.2751874	21.2393458	18.9100473
60	70.2733845	69.1162908	68.5268478	28.5993079	28.3007863	26.7801676
90	73.7053971	68.7468428	66.8831017	31.9738882	29.034606	24.7409313
Rep	1	2	3	1	2	3

Fig. S4c

	% $^{MBP-76}$EspP-^{His}BamA			
	Mock			
	S425C S1299C		G431C N1293C	
time(min)	PK- MOCK chase	PK+ MOCK chase	PK- MOCK chase	PK+ MOCK chase
0.5	0.706199925	4.204987281	1.59096519	4.663796082
2	0.729685214	7.37820867	3.108503701	4.553422791
5	0.834783546	5.116974049	1.959322218	4.150137224
15	0.364039998	6.830416736	2.051029021	4.75071763

Fig. S4c

	% $^{MBP-76}$EspP-^{His}BamA			
	Reduction chase			
	S425C S1299C		G431C N1293C	
time(min)	PK- DTT chase	PK+ DTT chase	PK- DTT chase	PK+ DTT chase
0.5	1.308664727	31.07764269	4.841971115	27.41144091
2	4.457443827	80.57262072	5.372833263	78.65851737
5	10.28340795	89.38206413	5.484029043	93.59390993
15	15.55681291	90.27688585	10.24144453	96.79617433

Fig. S5b

% $^{MBP-76}$EspP-^{His}BamA		
N805C R1044C	G807C W1042C	T809C G1040C
0.20445881	0	0.44236967
0.03095991	0	0.54664503
0.32618413	0.39017903	1.02959127
0.23833361	0.2846655	0.51980285

Fig. S6c

% $^{MBP-76}$EspP-^{His}BamA							
I806C G1040C	I806C A1041C	I806C W1042C	I806C A1043C	I806C R1044C	I806C I1045C	I806C M1046C	I806C S1047C
0	0	0.81185228	0.08963907	0.02589334	0.75434365	0	0
0	0	0.27014353	0.23113046	1.00218606	0.62221957	0	0
0	0	0.15572396	0.32535144	0.15466229	0.41696882	0.14299547	0
0	0	0.76895332	0.48438745	0.20318182	0.15870054	0	0.05143953

Fig. S6c

% $^{MBP-76}$EspP-^{His}BamA							
F785C G1040C	F785C A1041C	F785C W1042C	F785C A1043C	F785C R1044C	F785C I1045C	F785C M1046C	F785C S1047C
0.600927035	0.047397899	0.479816666	0	0.713714354	0.176759665	1.129423589	1.381225175
0.796348782	0.031384877	0.774373008	0	0.61281406	0.189566604	1.417802989	1.238771232
0.676654443	0.130373969	0.372762138	0.400808265	0.559378967	0.046271709	1.753199936	1.265764104
0.079068354	0.05283492	0.216837279	0.170993903	0.323921635	0.340341308	0.293385829	0.205700691

Fig. S8b

	% $^{MBP-76}$EspP-^{His}BamA					
Time (min)	S425C S1299C			G781C A1043C		
0	5.0448374	5.2146359	6.8454717	0.3349438	0.3682011	0.4145963
2	4.807096	5.9110087	6.3229716	0.2847438	0.2482138	0.8688313
5	4.4037897	5.62821	5.0364751	0.28615	0.5907829	0.373863
15	4.6874143	5.2215133	5.1154272	0.4538203	0.3658471	0.7481522
30	4.283202	5.1240133	5.4967144	0.1382771	0.0482554	0.7435212
60	4.9600896	5.5195021	6.3374345	0.4217434	0.3358566	0.6934161
90	6.034023	6.6199469	6.6397909	0.2856925	0.4253689	0.5916333
Rep	1	2	3	1	2	3

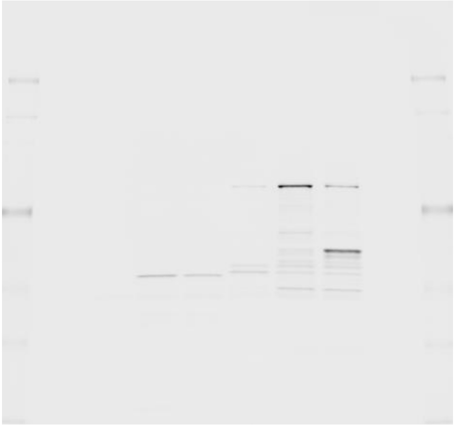

Fig 1d top aStrepII

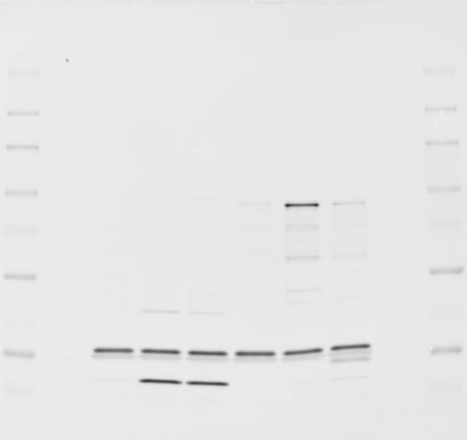

Fig 1d mid $aEspP_{bN}$

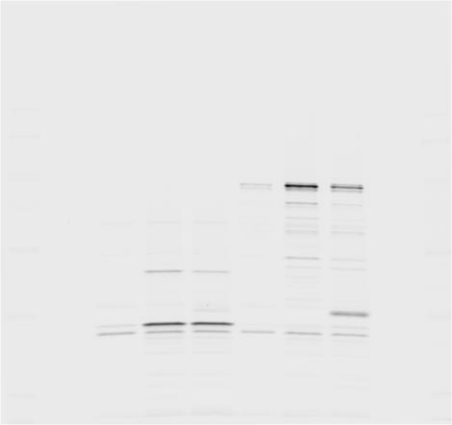

Fig 1d bot aEspP$_{bC}$

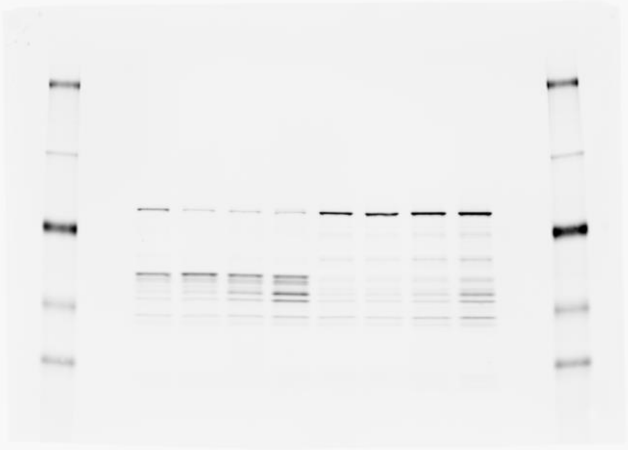

Fig 1e top aStrepII

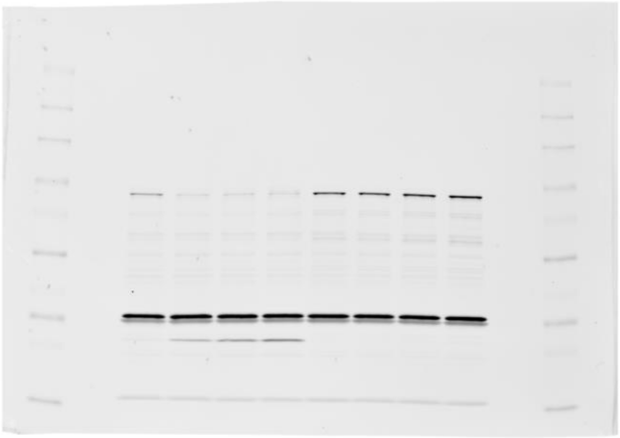

Fig 1e mid aEspP$_{bN}$

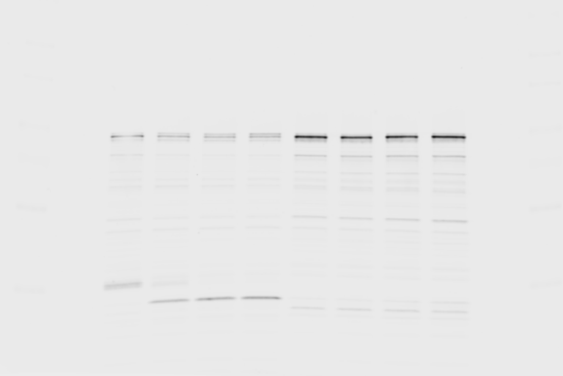

Fig 1e bot aEspP$_{bC}$

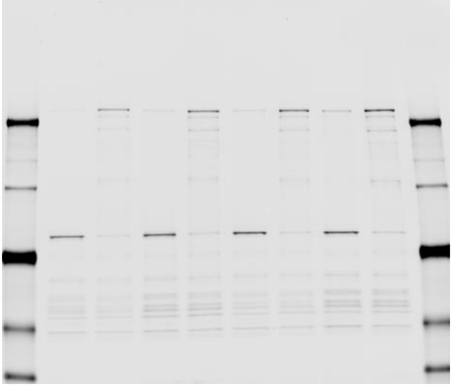

Fig 2b left aStrepII

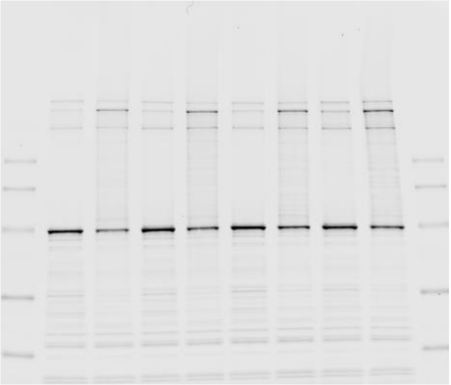

Fig 2b right aBamA_C

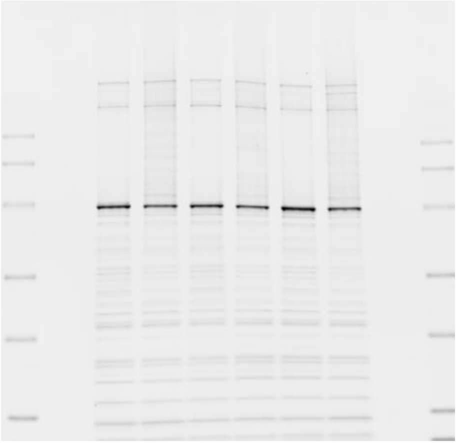

Fig 2c bot aBamA$_C$

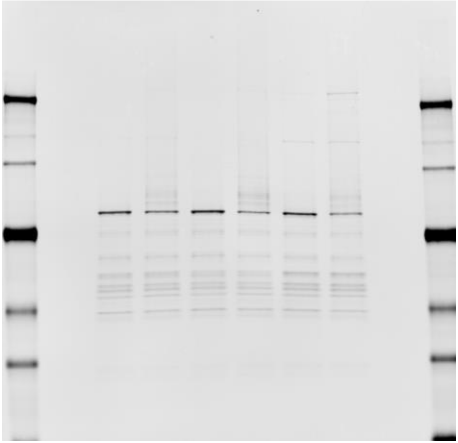

Fig 2c top aStrepII

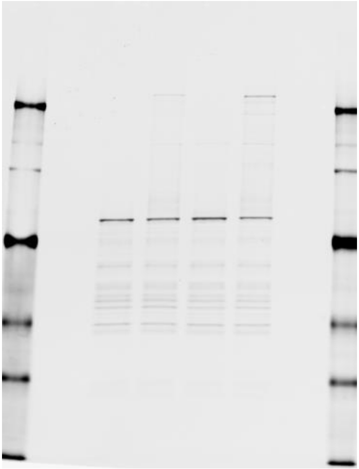

Fig 2d left aStrepII

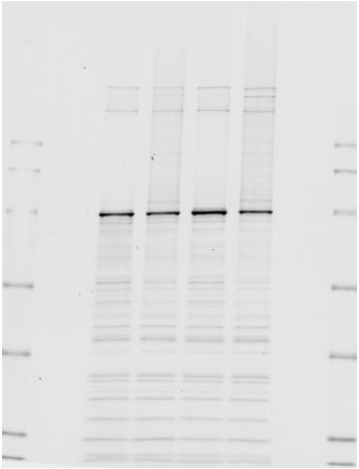

Fig 2d right aBamA$_C$

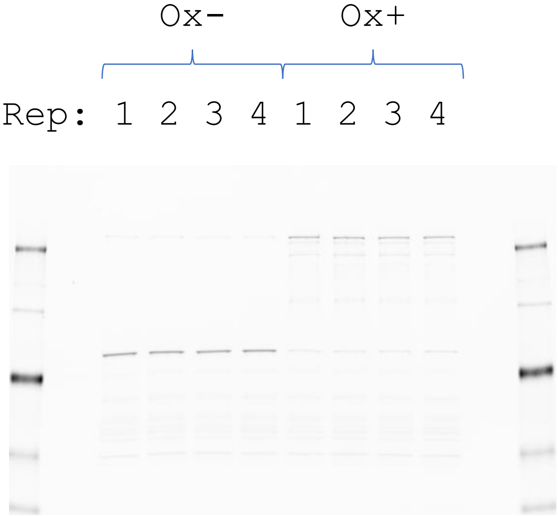

Fig 2e quant G429C N1295C aStrepII

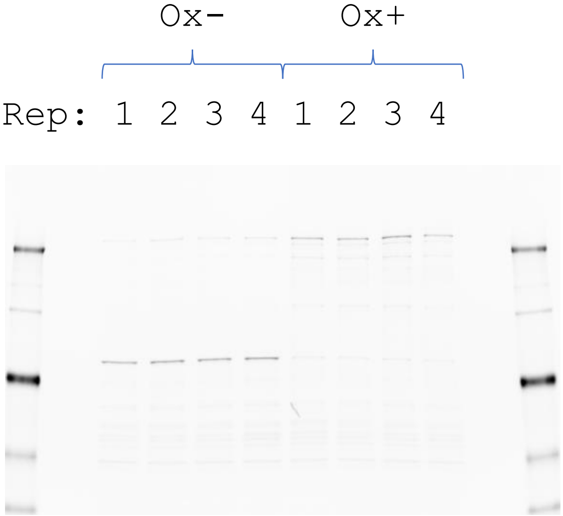

Fig 2e quant G431C N1293C aStrepII

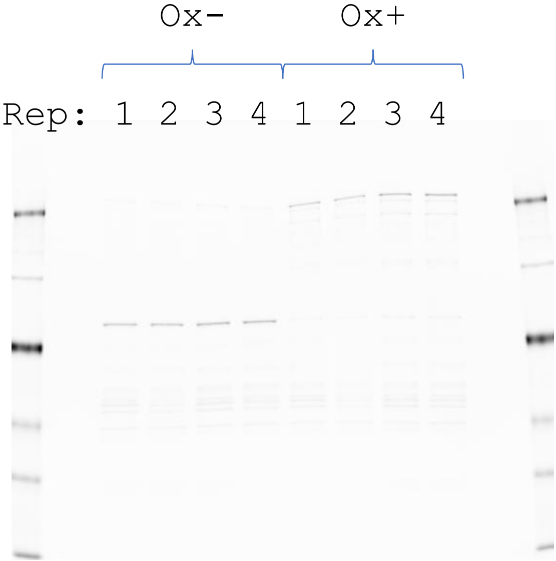

Fig 2e quant N427C R1297C aStrepII

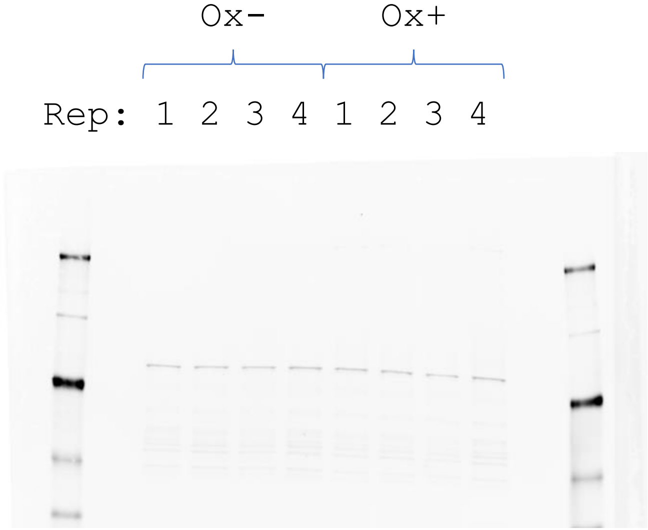

Fig 2e quant N427C Y1298C aStrepII

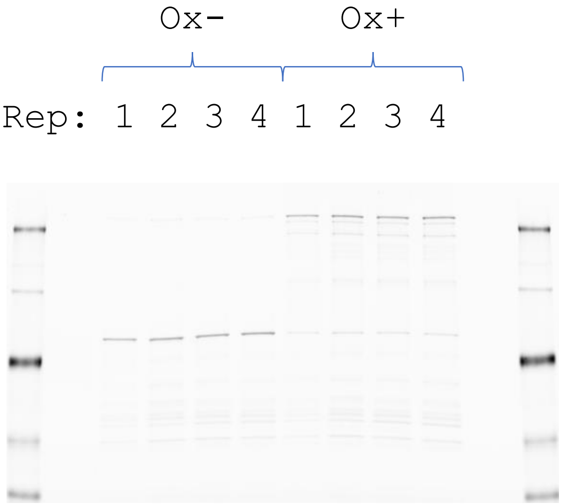

Fig 2e quant S425C S1299C aStrepII

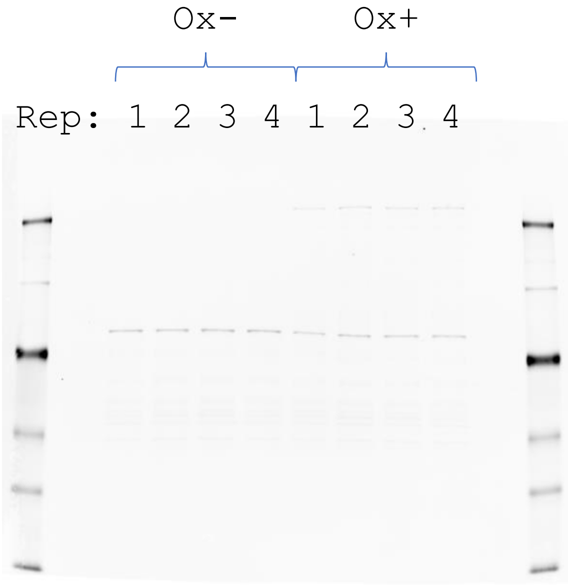

Fig 2e quant S425C Y1298C aStrepII

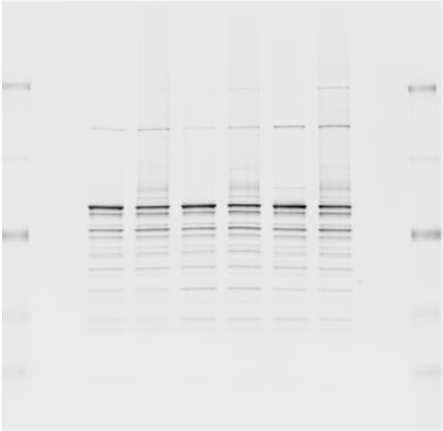

Fig 3b bot aBamA$_C$

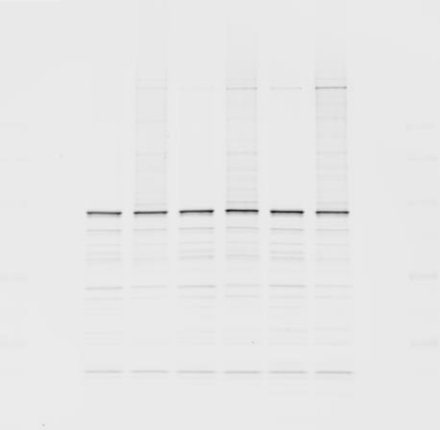

Fig 3b top aStrepII

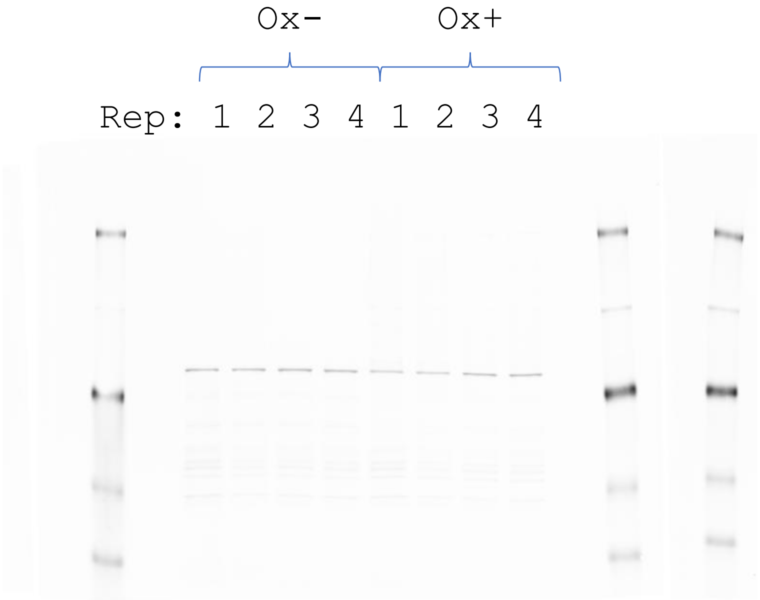

Fig 3c quant G807C W1042C aStrepII

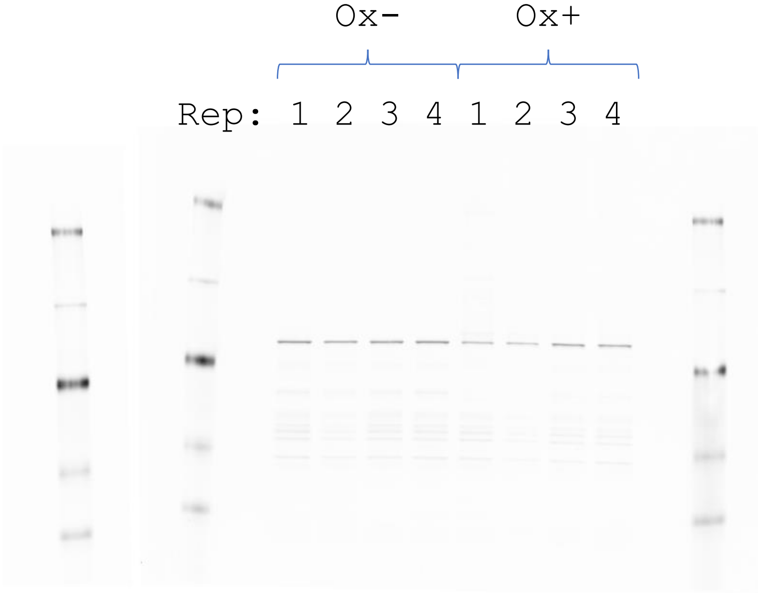

Fig 3c quant N805C R1044C aStrepII

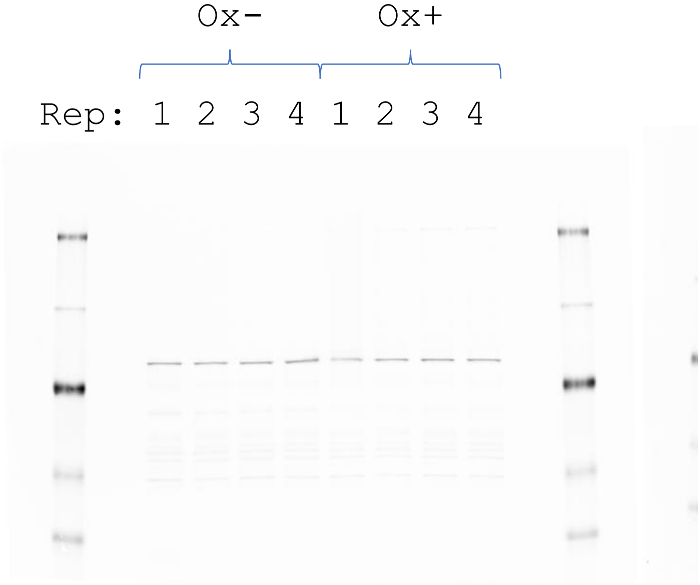

Fig 3c quant T809C G1040C aStrepII

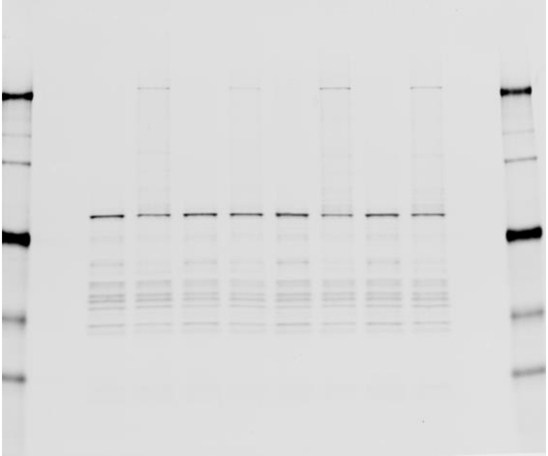

Fig 4b bot left aStrepII

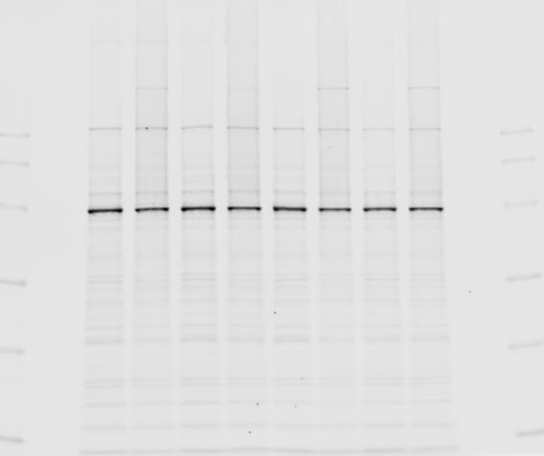

Fig 4b bot right $aBamA_C$

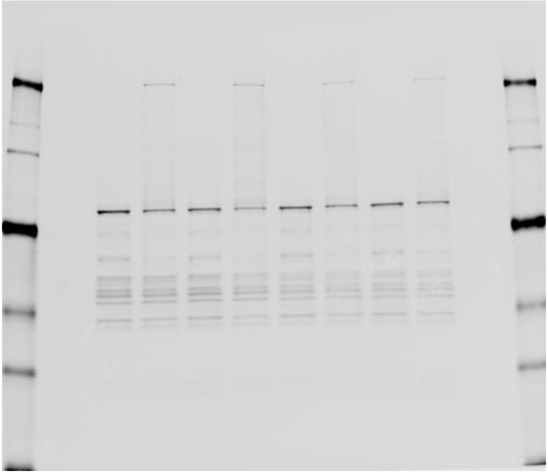

Fig 4b top left aStrepII

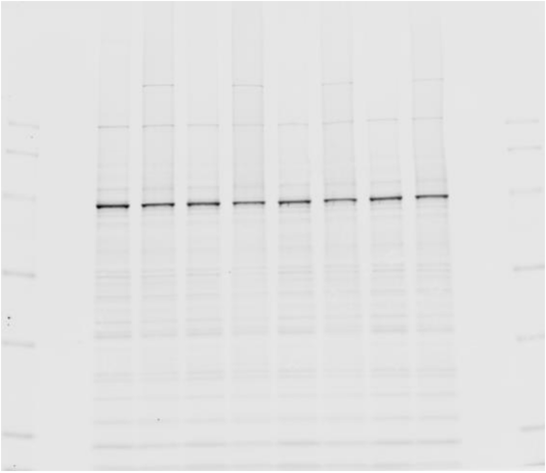

Fig 4b top right aBamA$_C$

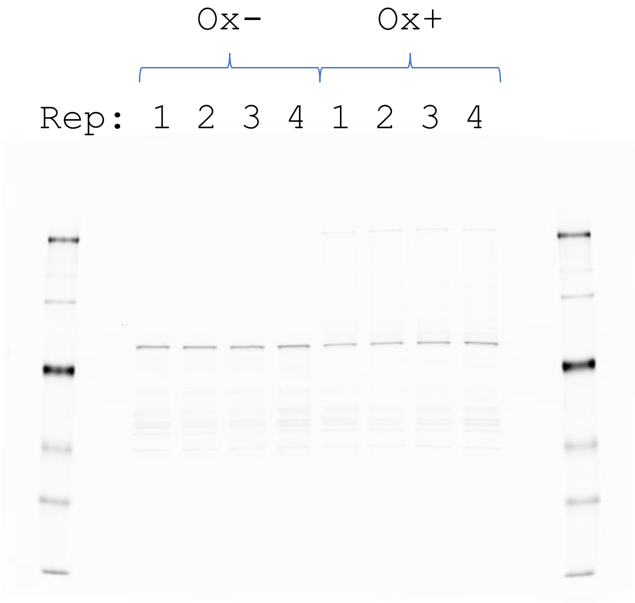

Fig 4c quant F785C A1041C aStrepII

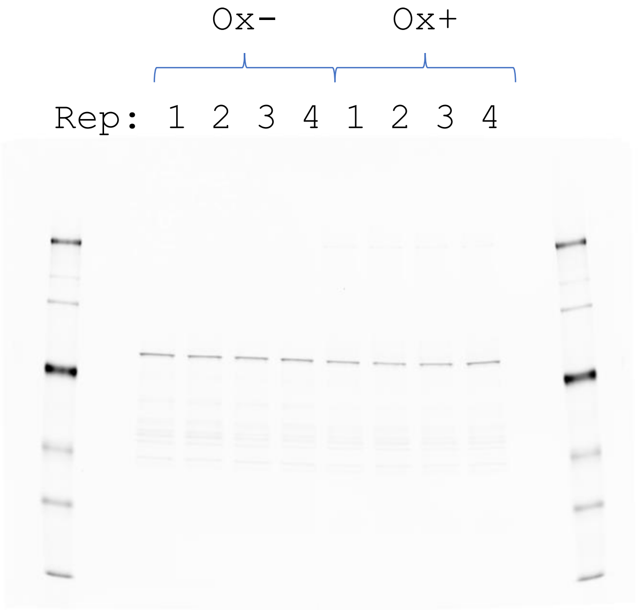

Fig 4c quant F785C A1043C aStrepII

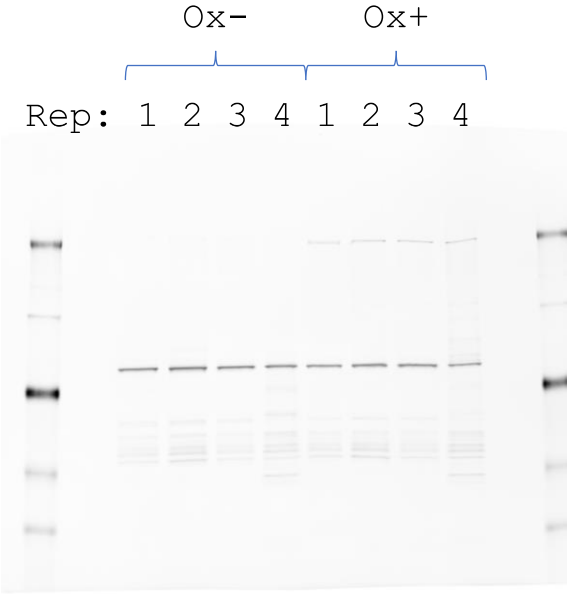

Fig 4c quant F785C G1040C aStrepII

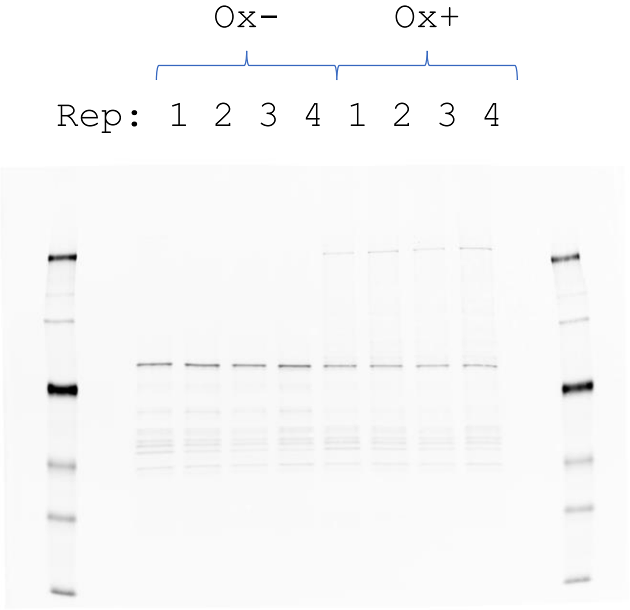

Fig 4c quant F785C I1045C aStrepII

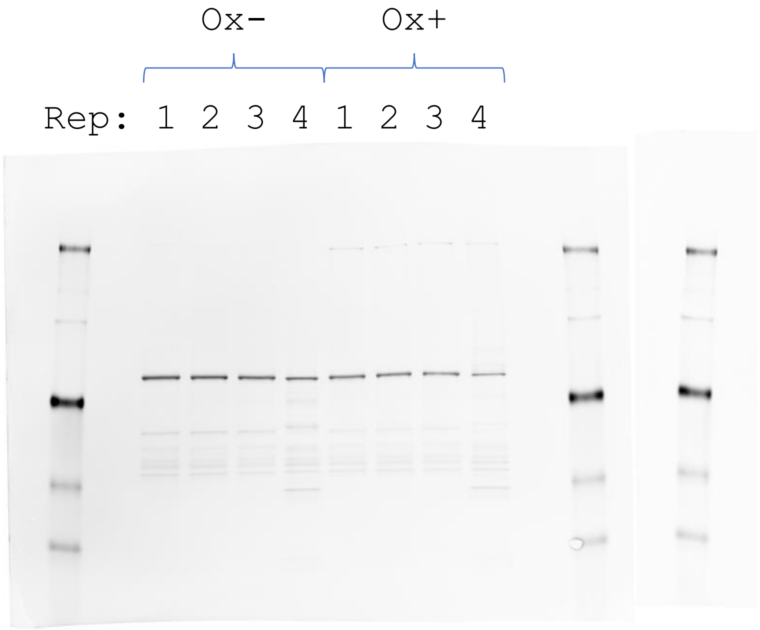

Fig 4c quant F785C R1044C aStrepII

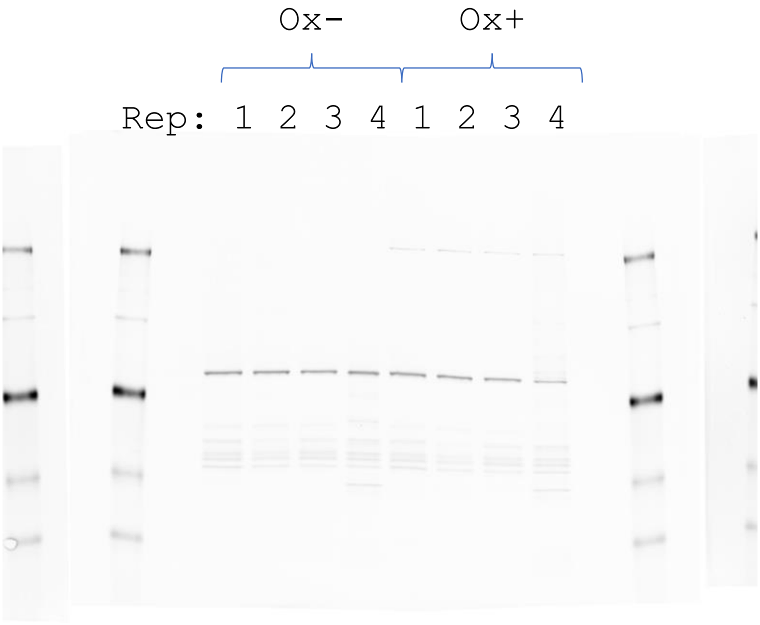

Fig 4c quant F785C W1042C aStrepII

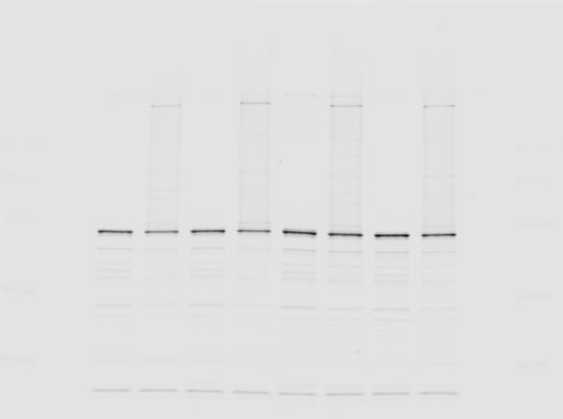

Fig 4d bot left aEspP$_{bC}$

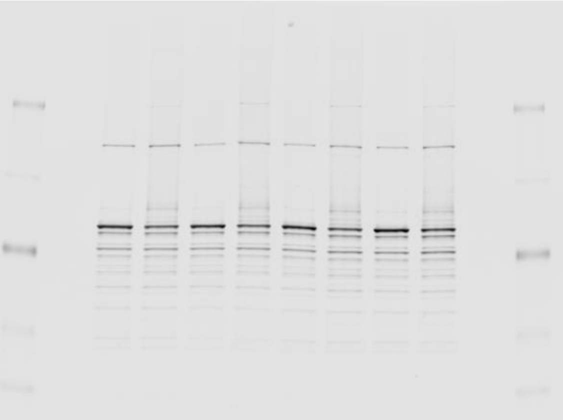

Fig 4d bot right aHis

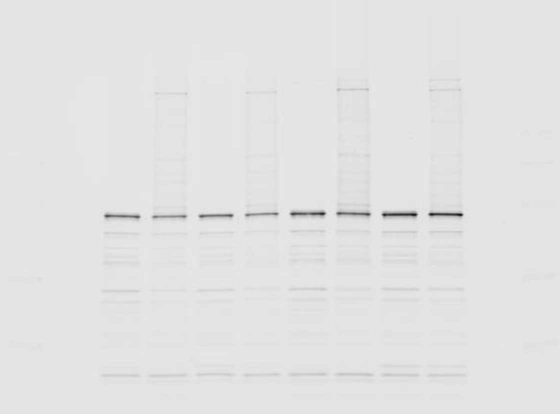

Fig 4d top left aEspP$_{bC}$

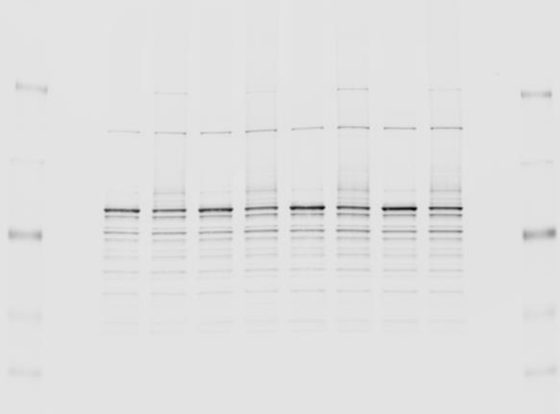

Fig 4d top right aHis

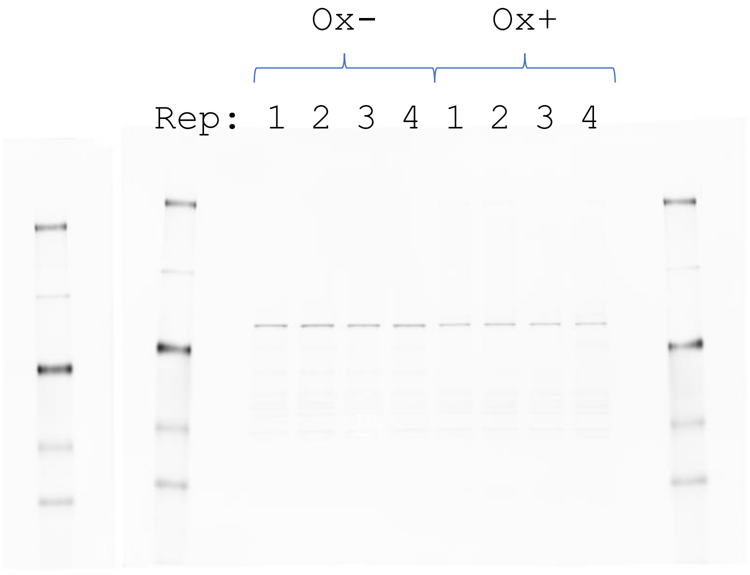

Fig 4e quant I806C A1041C aStrepII

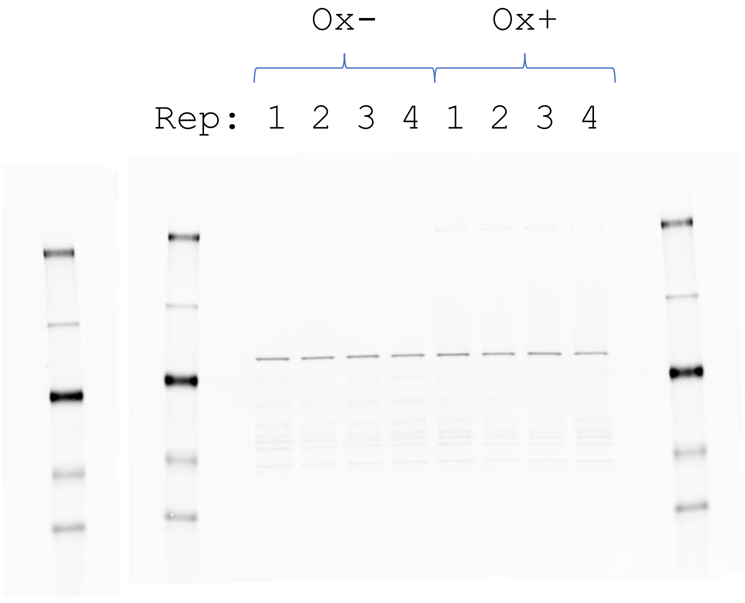

Fig 4e quant I806C A1043C aStrepII

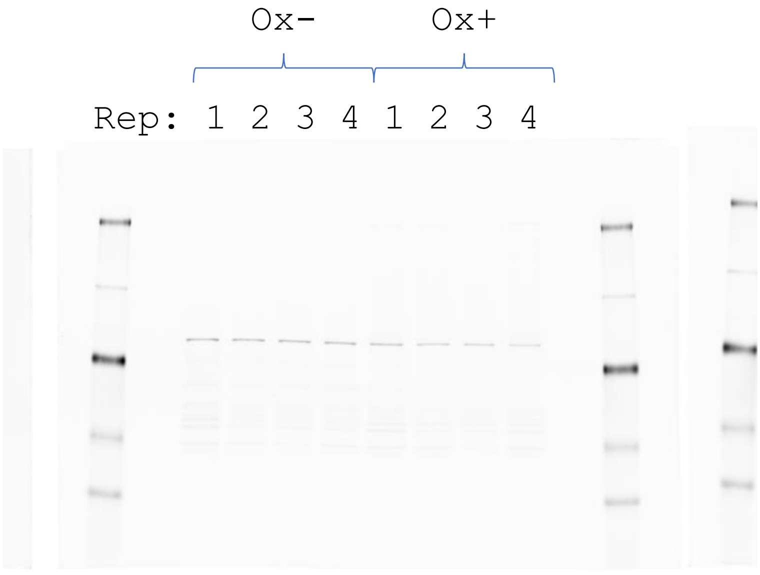

Fig 4e quant I806C G1040C aStrepII

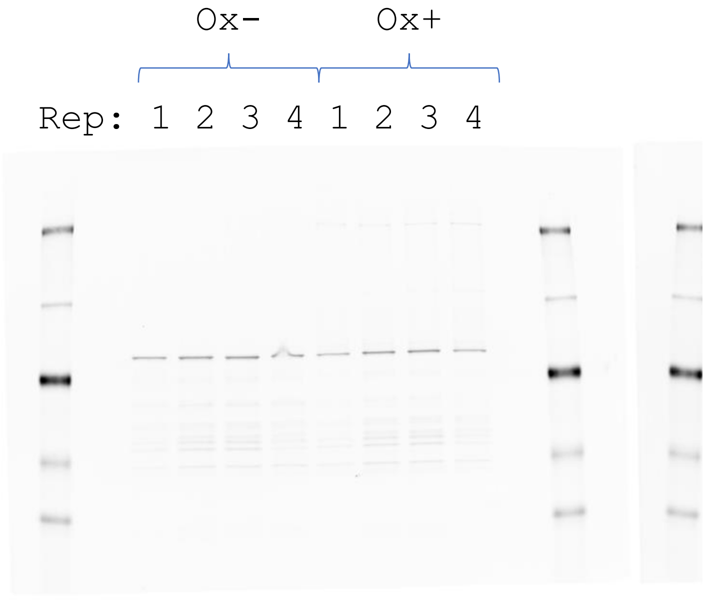

Fig 4e quant I806C I1045C aStrepII

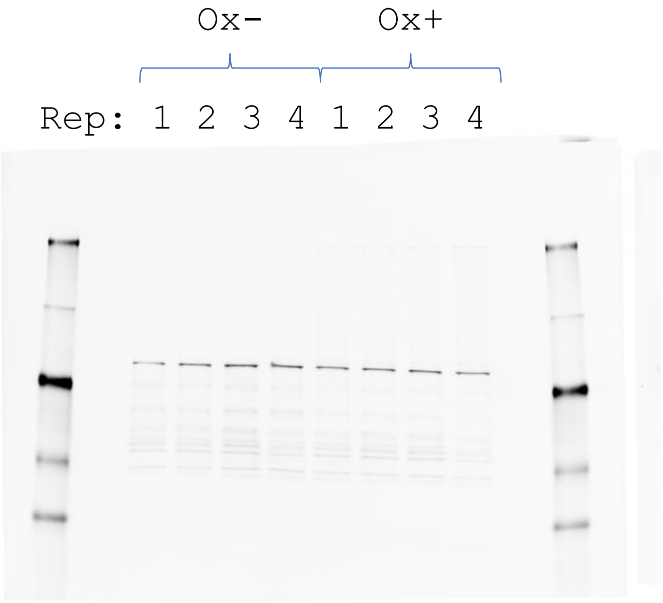

Fig 4e quant I806C M1046C aStrepII

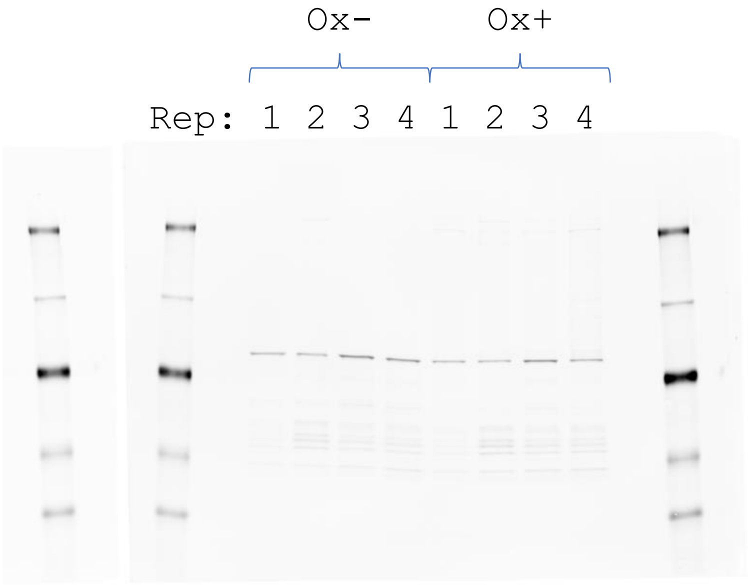

Fig 4e quant I806C R1044C aStrepII

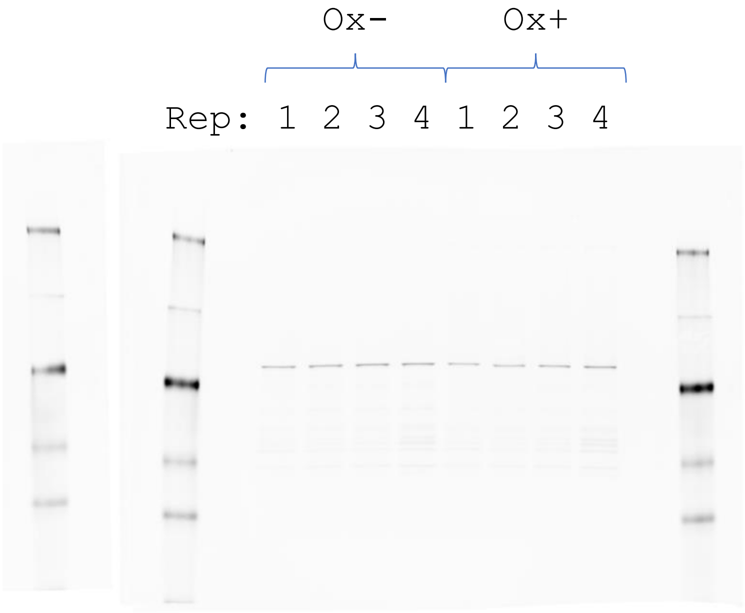

Fig 4e quant I806C S1047C aStrepII

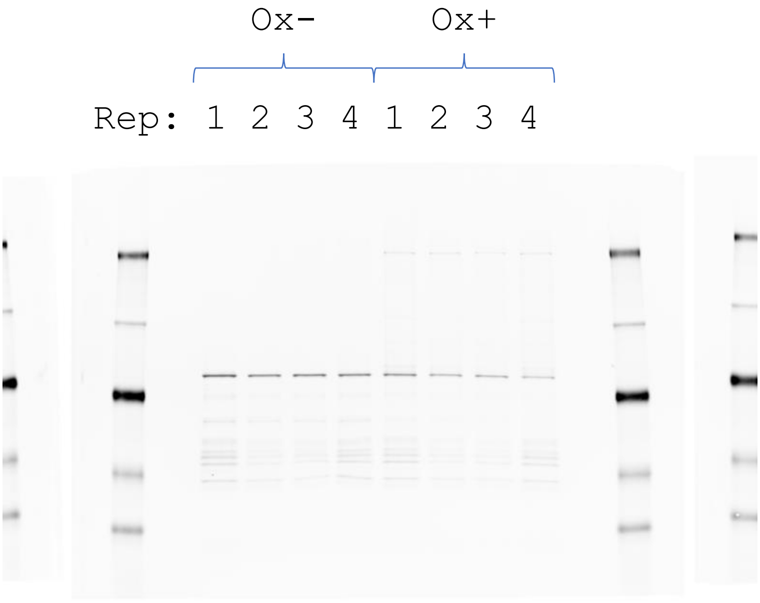

Fig 4e quant I806C W1042C aStrepII

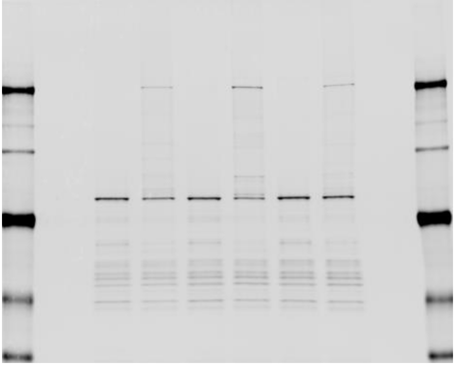

Fig 5b bot left aStrepII

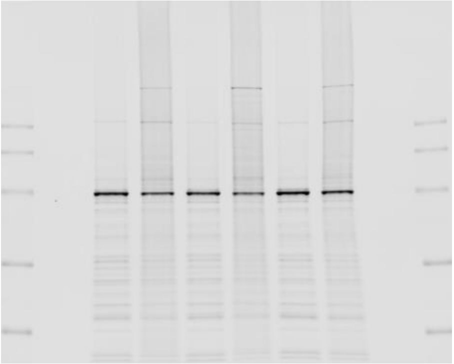

Fig 5b bot right aBamA$_C$

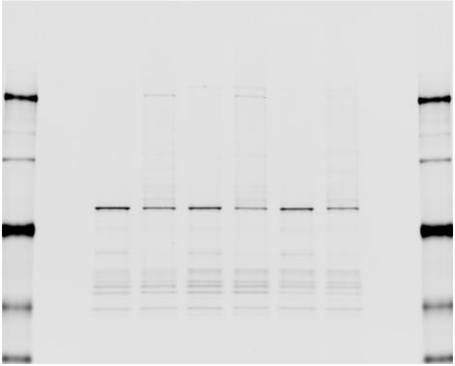

Fig 5b top left aStrepII

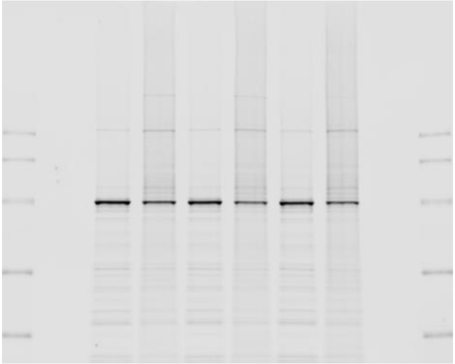

Fig 5b top right $aBamA_C$

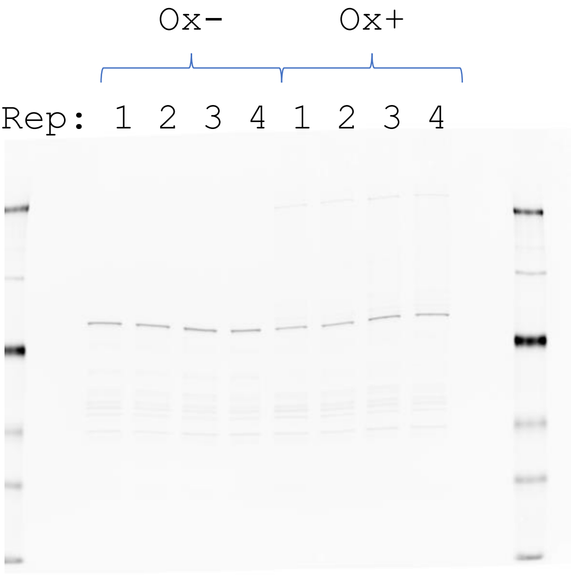

Fig 5C quant G781C A1041C aStrepII

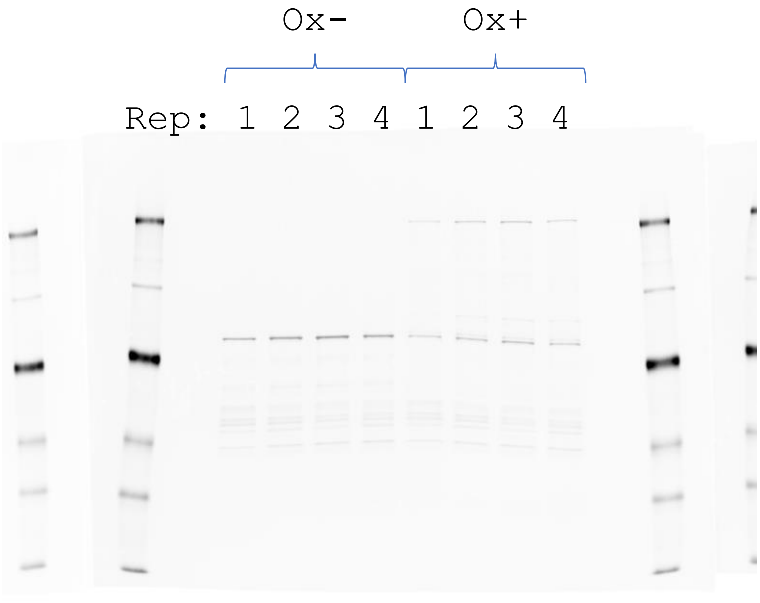

Fig 5C quant G781C A1043C aStrepII

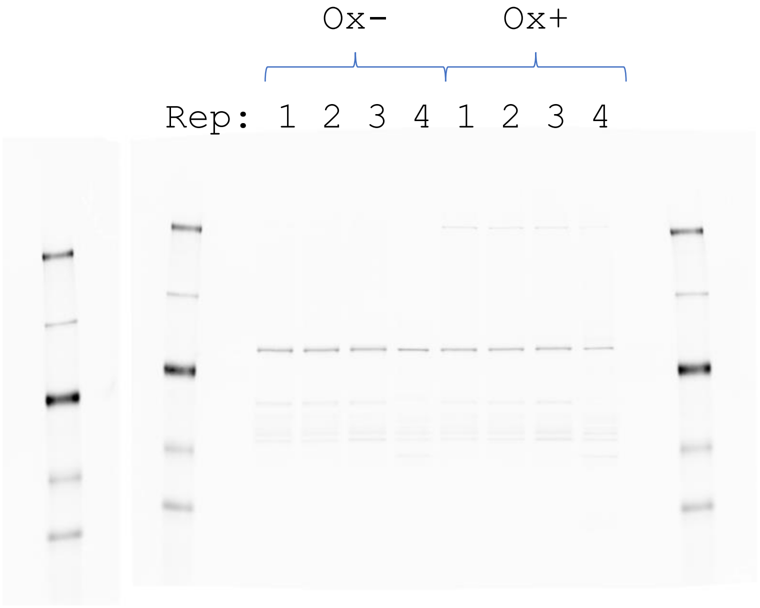

Fig 5C quant G781C G1040C aStrepII

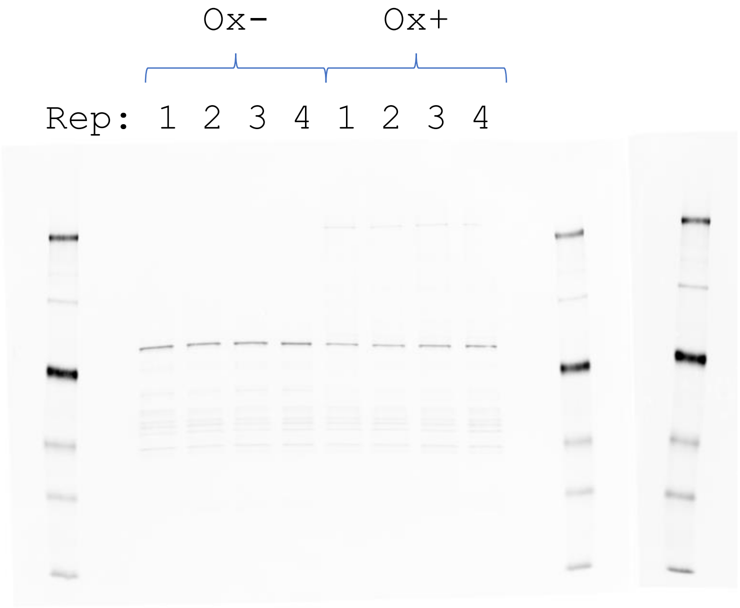

Fig 5C quant G781C I1045C aStrepII

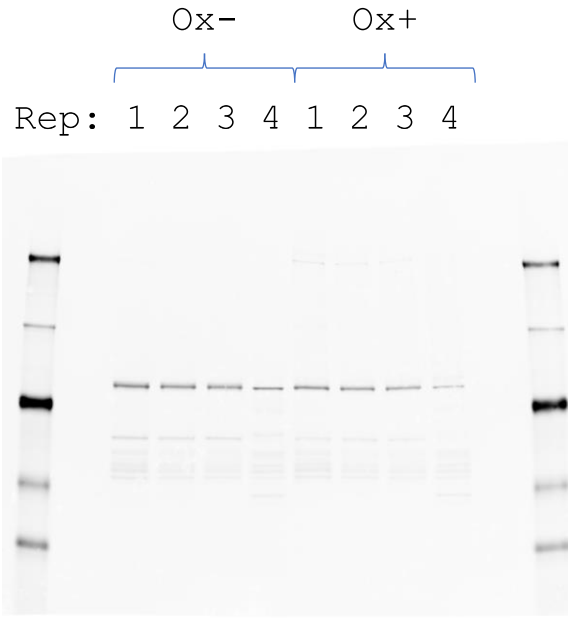

Fig 5C quant G781C R1044C aStrepII

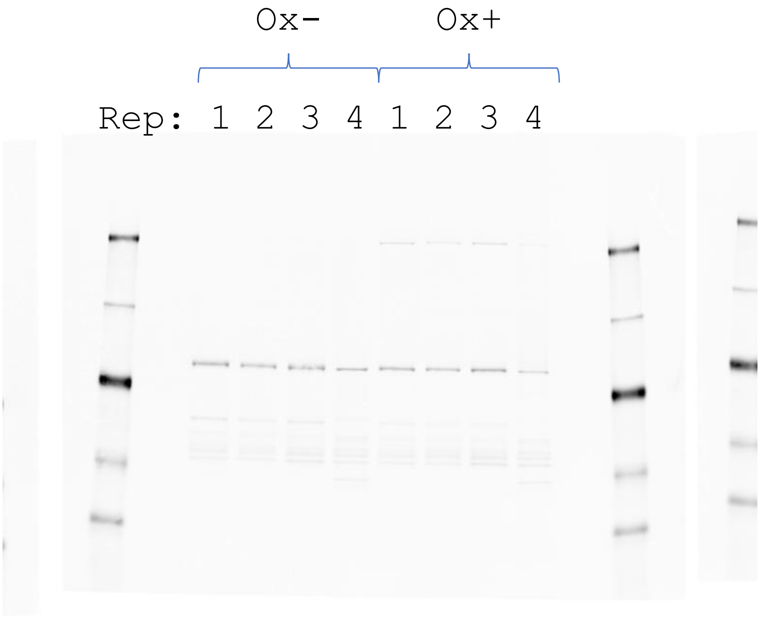

Fig 5C quant G781C W1042C aStrepII

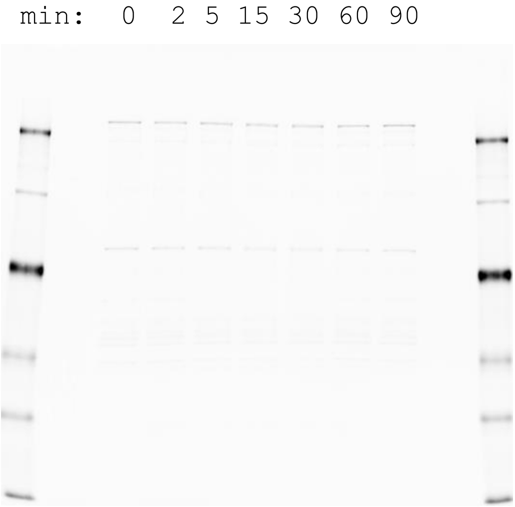

Fig 6b quant S1299C S425C rep 1 aStrepII

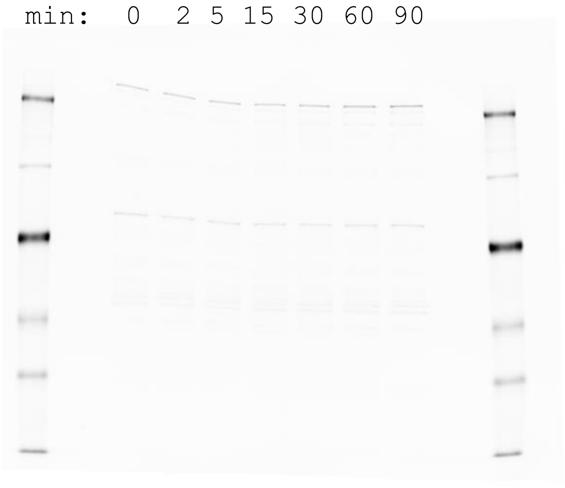

Fig 6b quant S1299C S425C rep 2 aStrepII

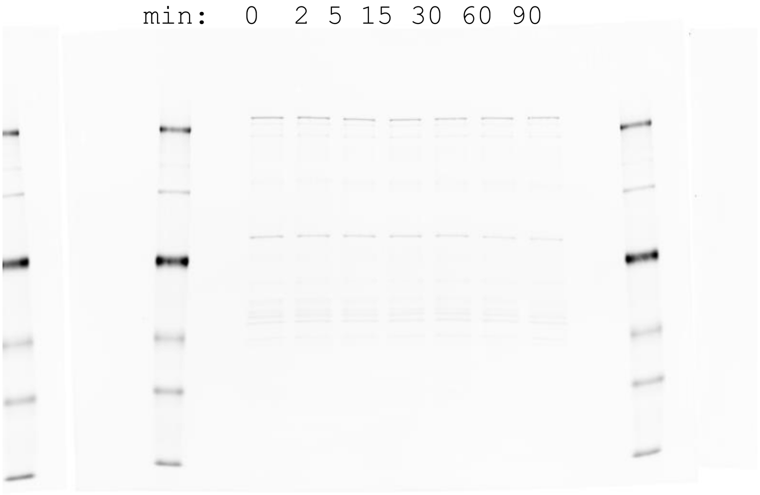

Fig 6b quant S1299C S425C rep 3 aStrepII

min: 0 2 5 15 30 60 90

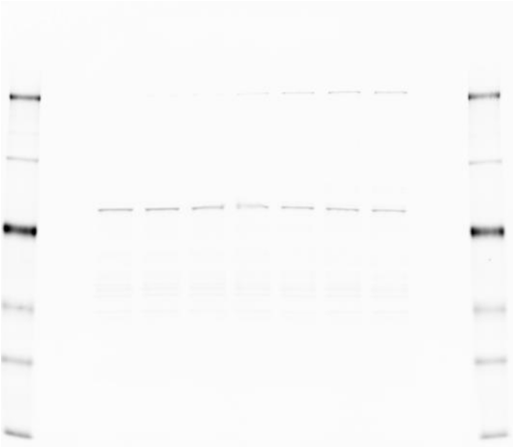

Fig 6b quant A1043C G781C rep 1
aStrepII

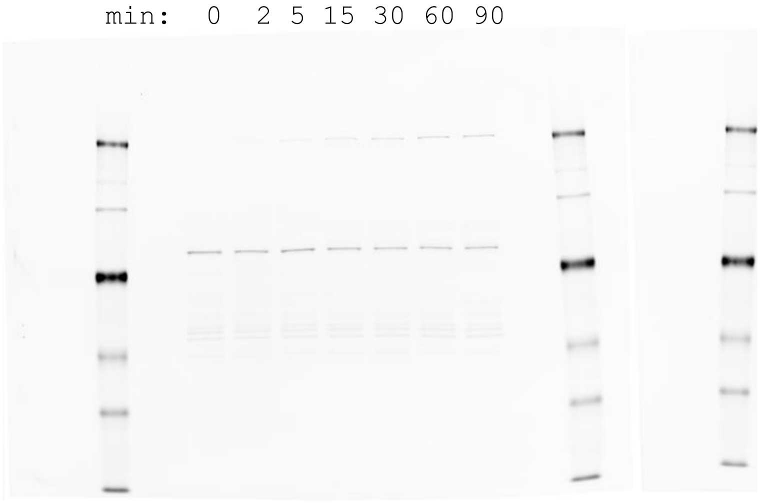

Fig 6b quant A1043C G781C rep 2 aStrepII

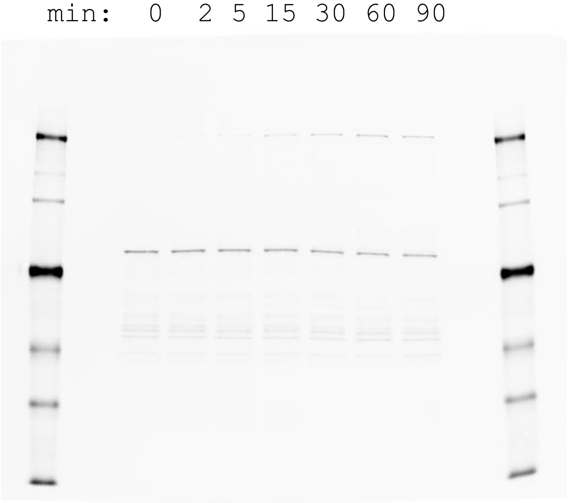

Fig 6b quant A1043C G781C rep 3 aStrepII

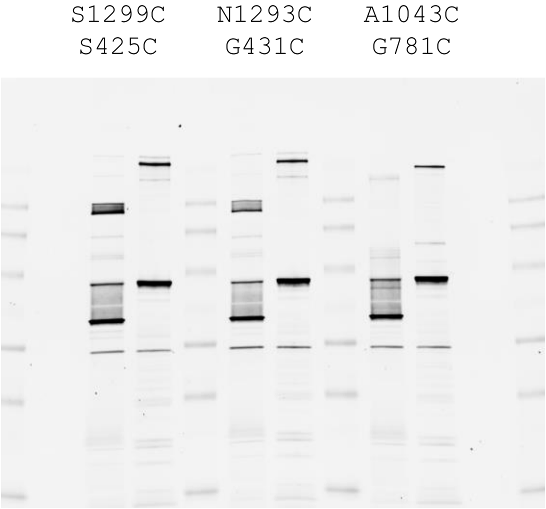

Fig 6c aBamA$_C$

S1299C S425C | N1293C G431C | A1043C G781C

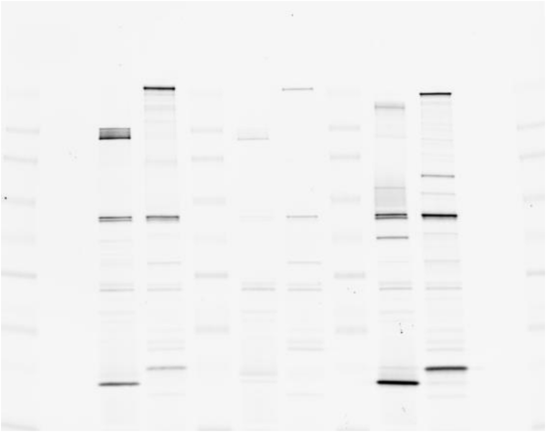

Fig 6c $aEspP_{bC}$

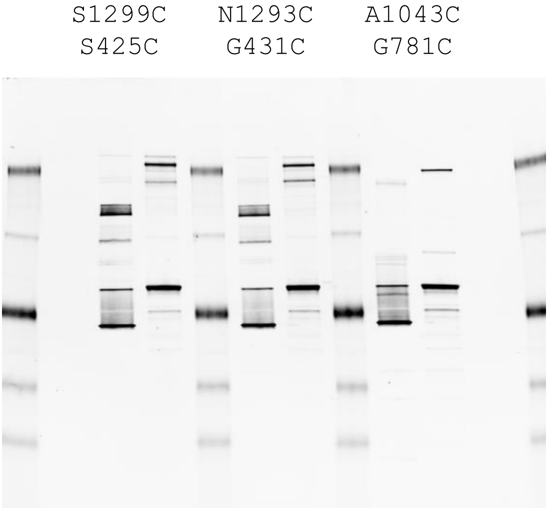

Fig 6c aHis

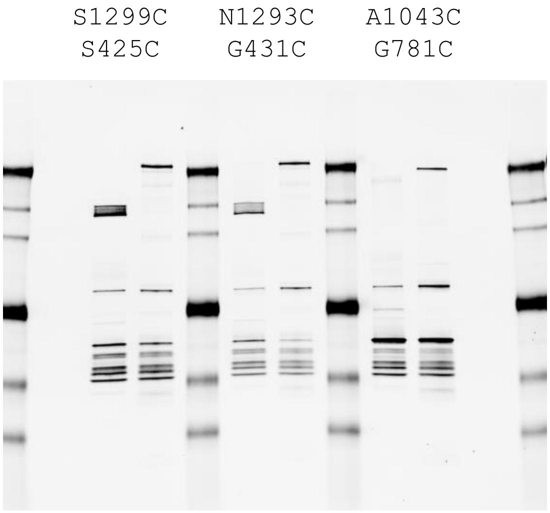

Fig 6c aStrepII

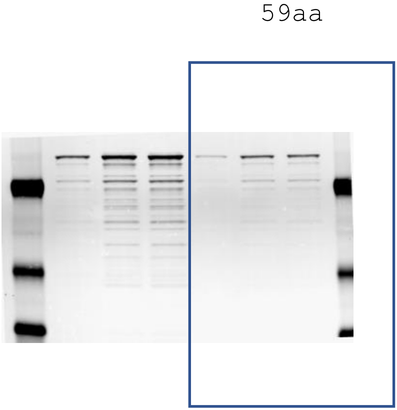

Fig S1a bot right aHis

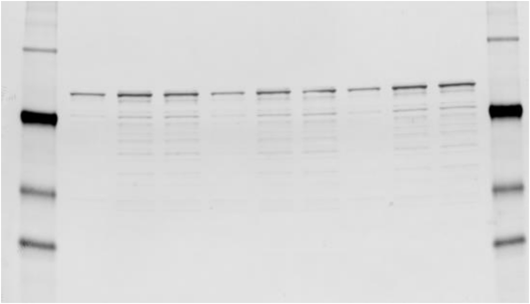

Fig S1a bot aHis

59aa

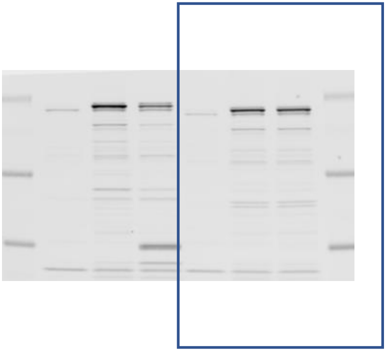

Fig S1a mid right $aEspP_{bC}$

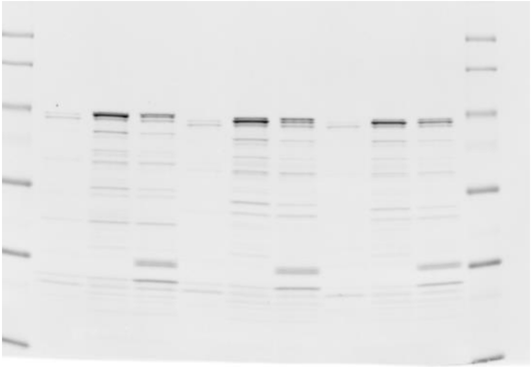

Fig S1a mid $aEspP_{bC}$

59aa

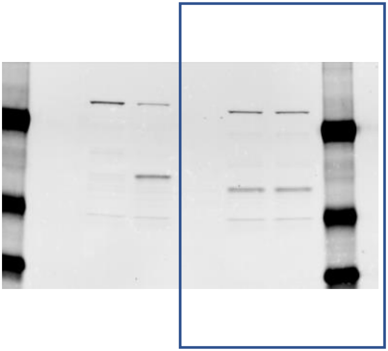

Fig S1a top right aStrepII

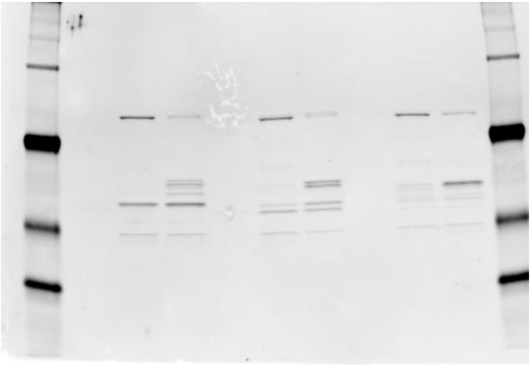

Fig S1a top aStrepII

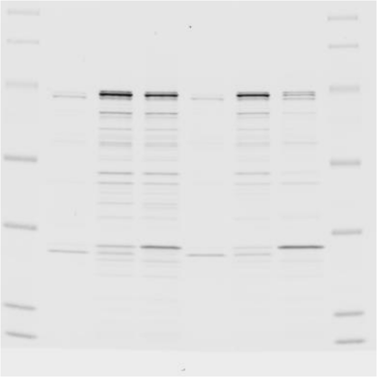

Fig S1b bot aEspP$_{bC}$

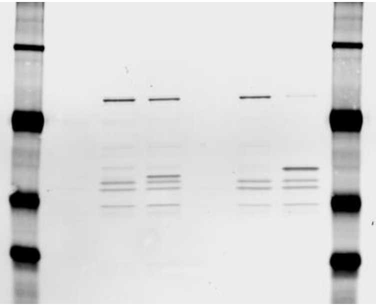

Fig S1b top aStrepII

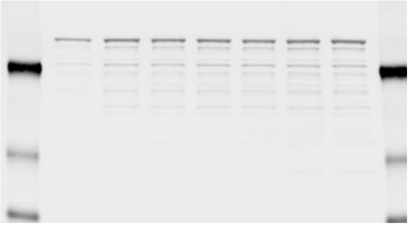

Fig S1c bot left aHis

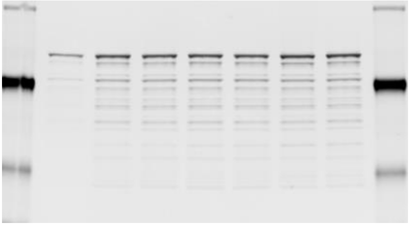

Fig S1c bot right aHis

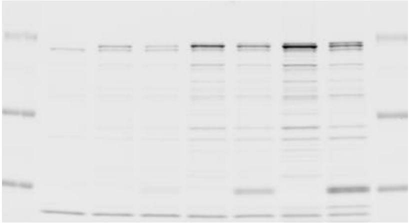

Fig S1c mid right aEspP$_{bC}$

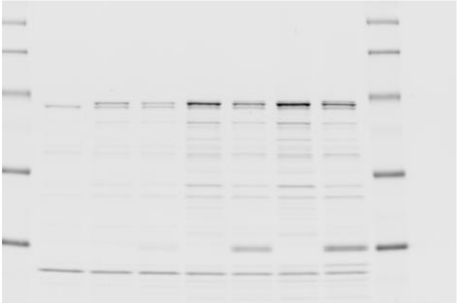

Fig S1c mid left aEspP$_{bC}$

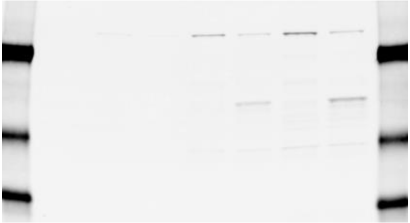

Fig S1c top left aStrepII

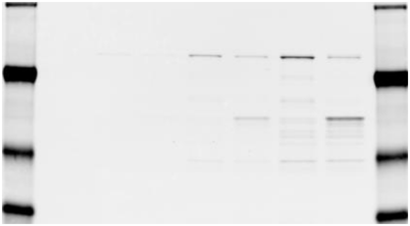

Fig S1c top right aStrepII

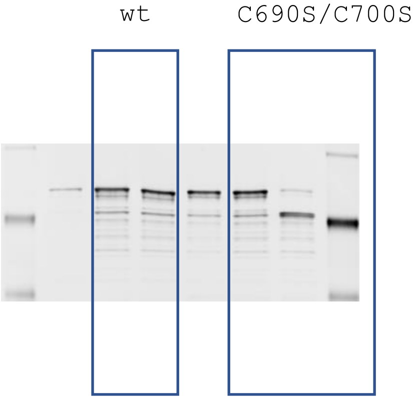

Fig S2a aHis

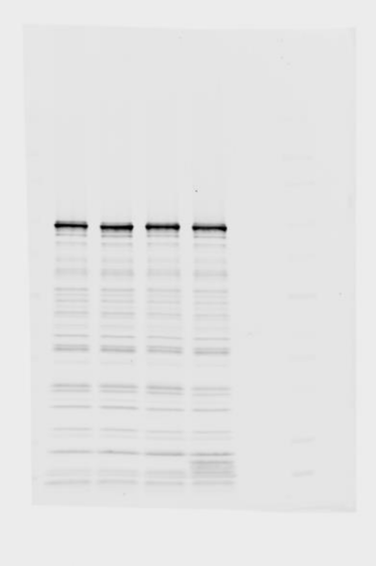

Fig S2b bot left $aBamA_C$

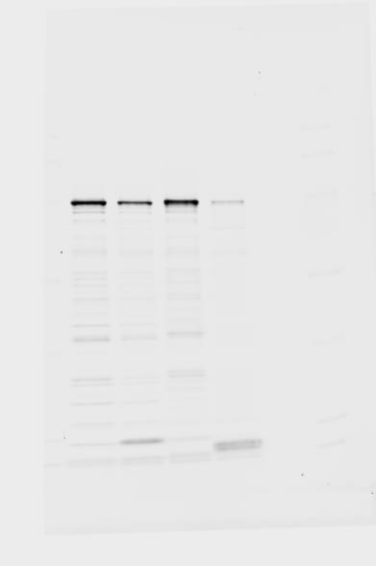

Fig S2b bot right $aBamA_C$

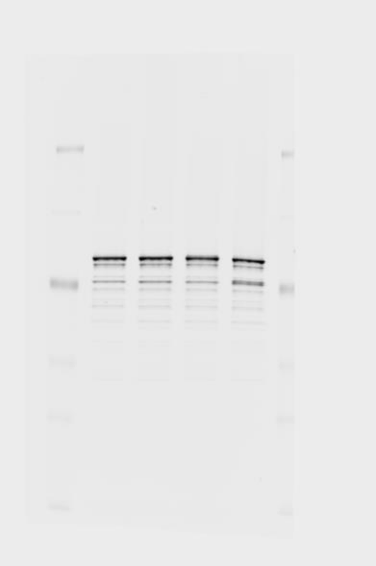

Fig S2b top left aHis

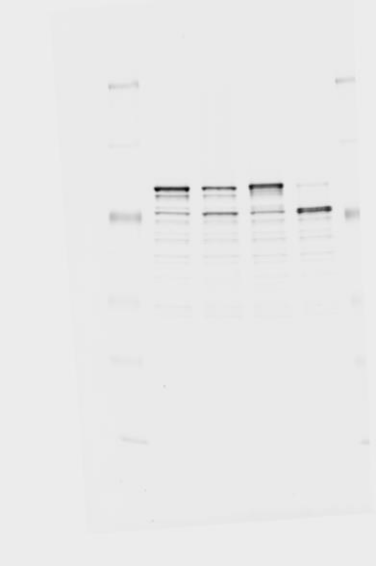

Fig S2b top right aHis

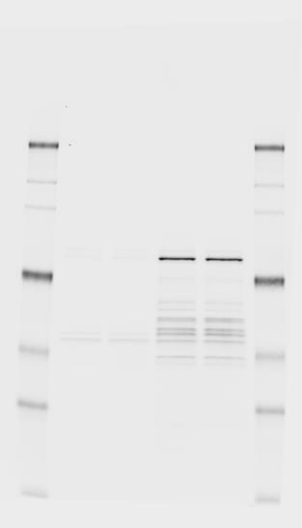

Fig S2c bot left aStrepII

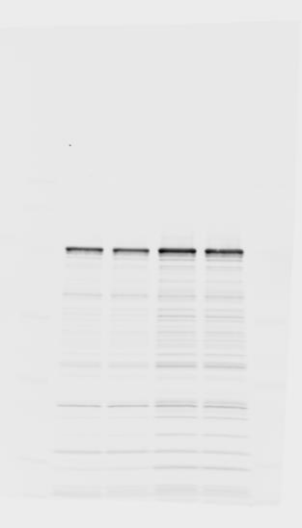

Fig S2c bot right aBamA$_C$

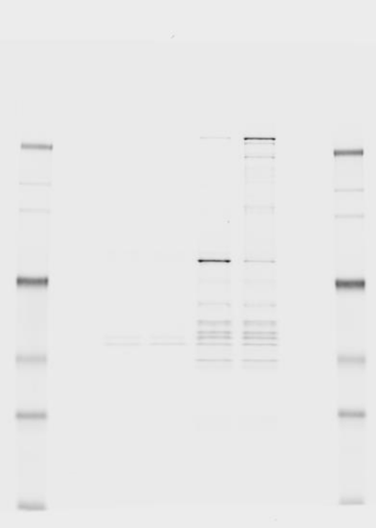

Fig S2c top left aStrepII

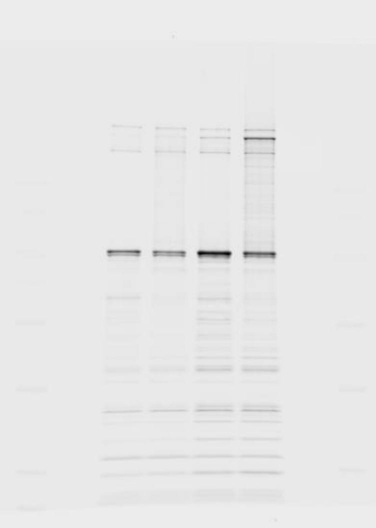

Fig S2c top right aBamA$_C$

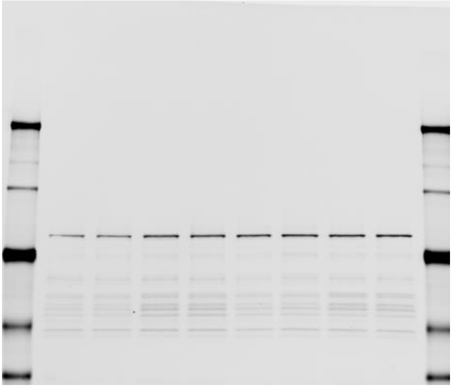

Fig S3a left aStrepII

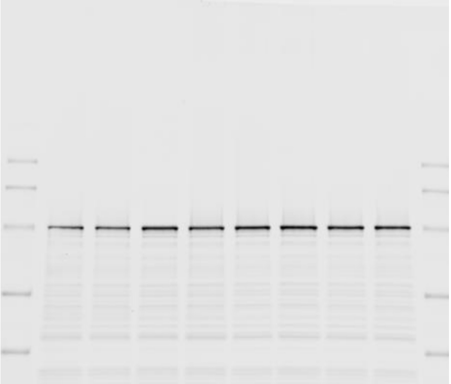

Fig S3a right aBamA$_C$

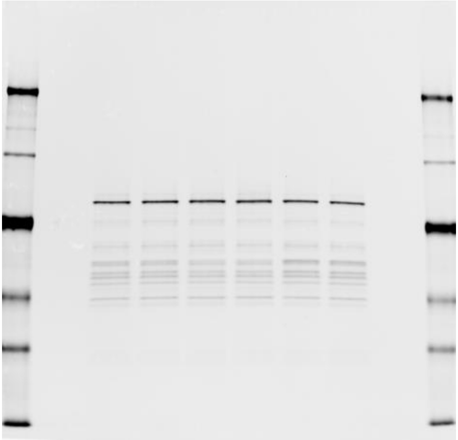

Fig S3b left aStrepII

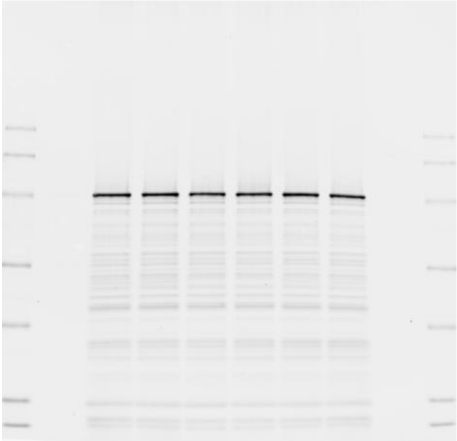

Fig S3b right $aBamA_C$

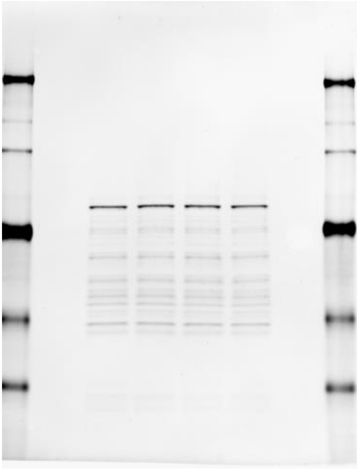

Fig S3c left aStrepII

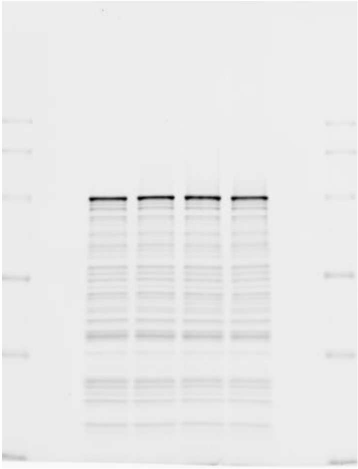

Fig S3c right aBamA$_C$

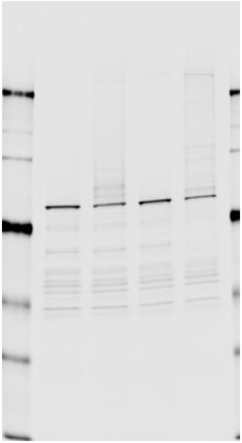

Fig S3d far left aStrepII

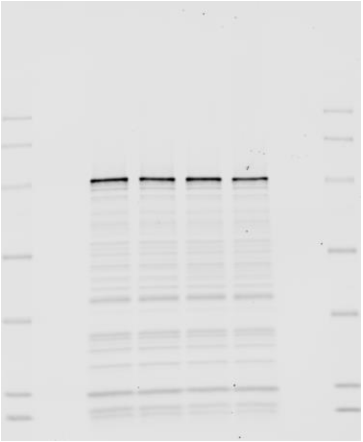

Fig S3d far right $aBamA_C$

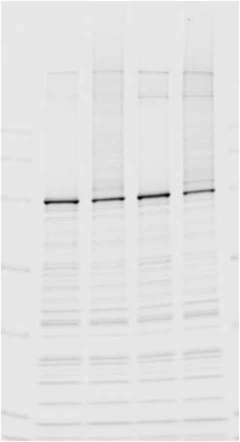

Fig S3d left aBamA$_C$

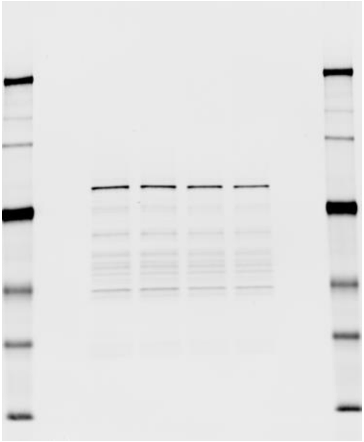

Fig S3d right aStrepII

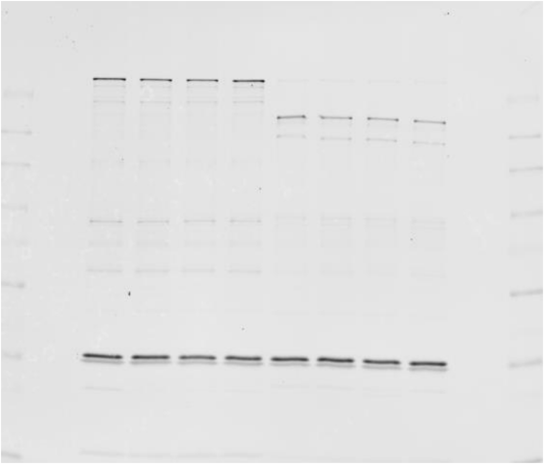

Fig S4a bot far left aEspP$_{bN}$

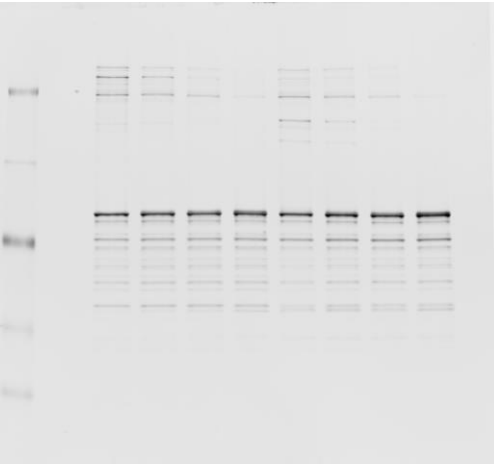

Fig S4a bot far right aHis

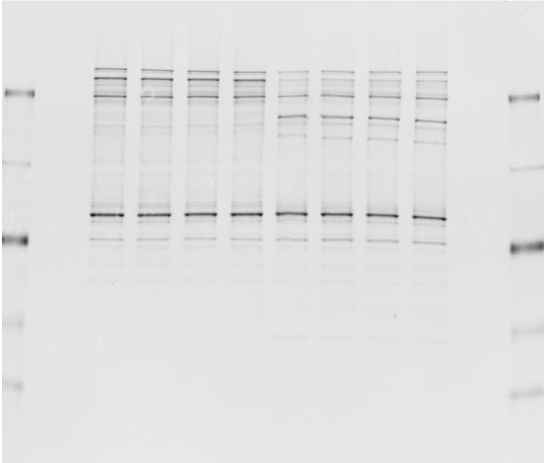

Fig S4a bot left aHis

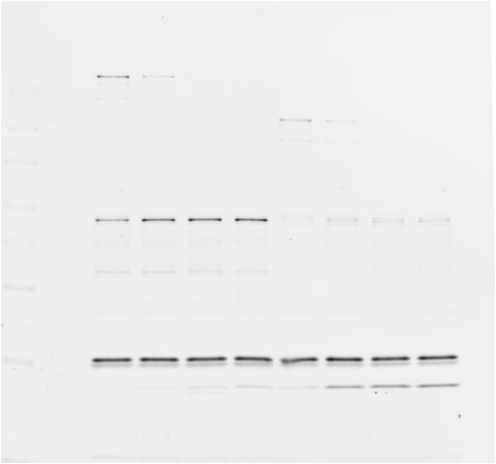

Fig S4a bot right aEspP$_{bN}$

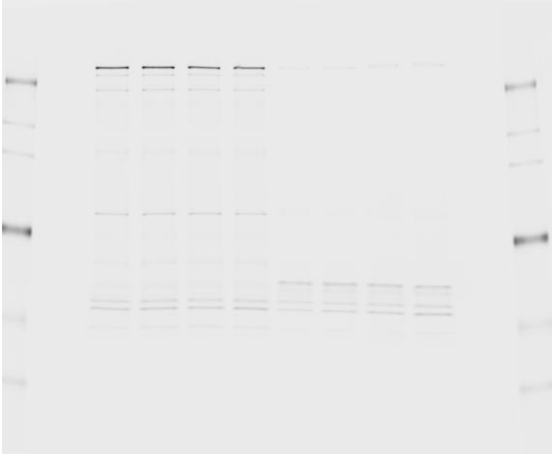

Fig S4a top far left aStrepII

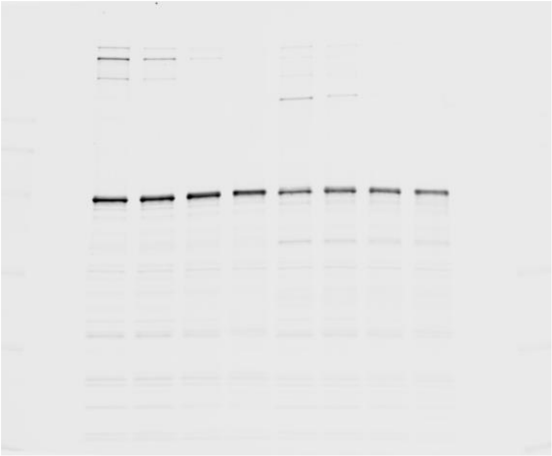

Fig S4a top far right aBamA$_C$

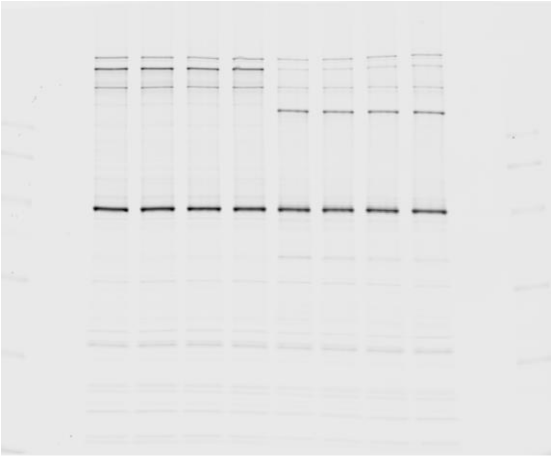

Fig S4a top left aBamA$_C$

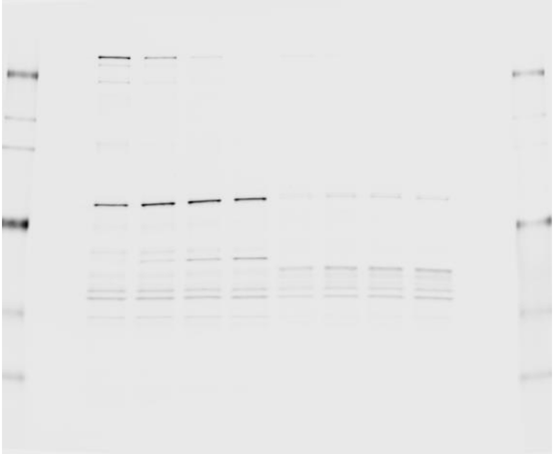

Fig S4a top right aStrepII

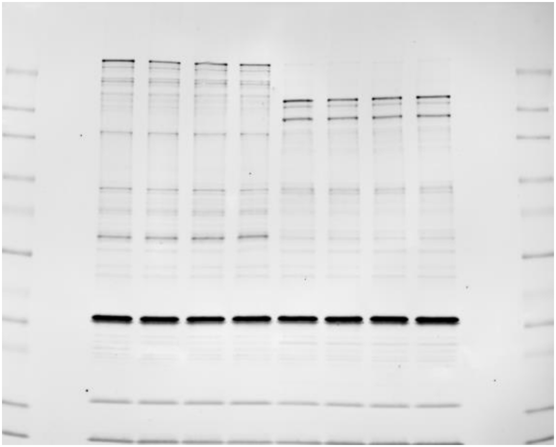

Fig S4b bot far left $aEspP_{bN}$

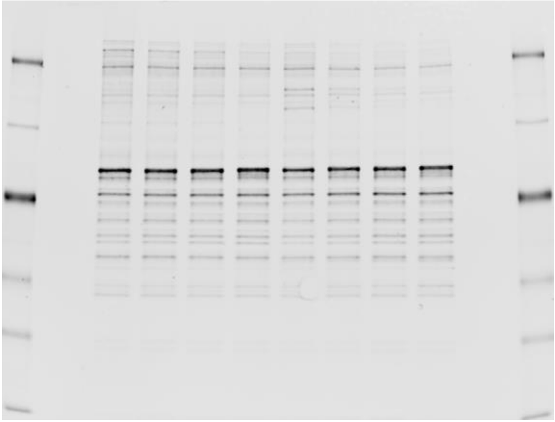

Fig S4b bot far right aHis

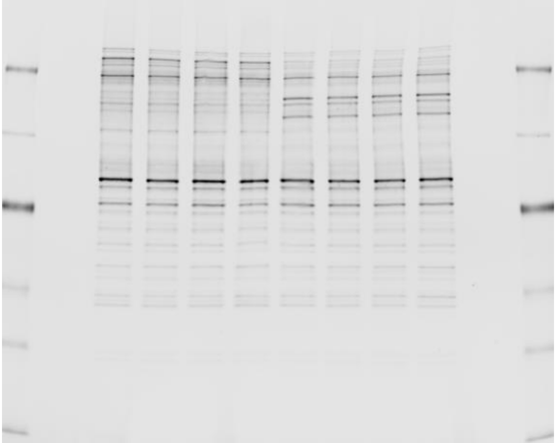

Fig S4b bot left aHis

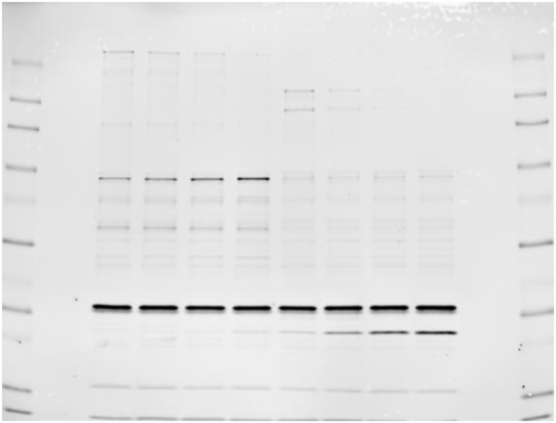

Fig S4b bot right $aEspP_{bN}$

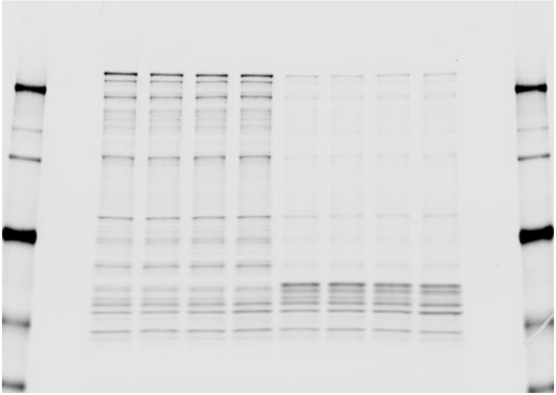

Fig S4b top far left aStrepII

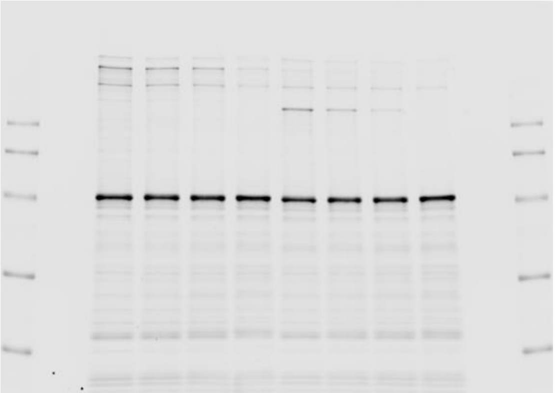

Fig S4b top far right aBamA$_C$

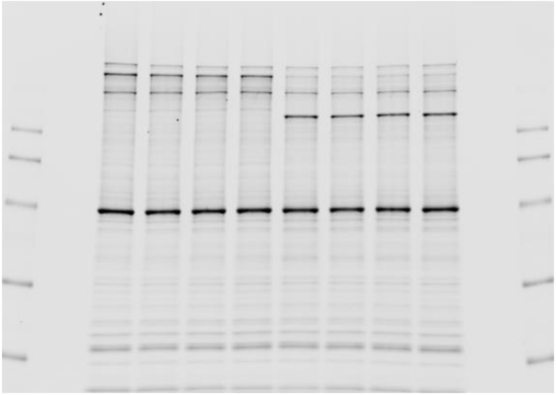

Fig S4b top left aBamA_C

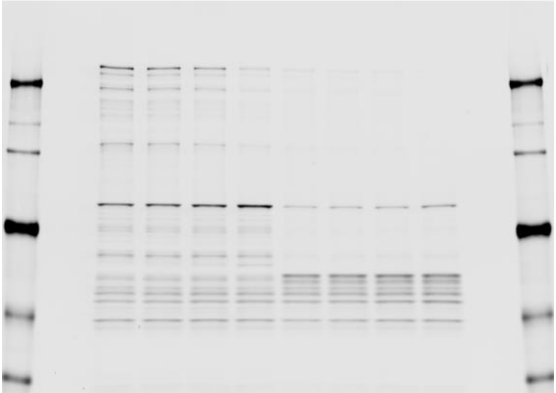

Fig S4b top right aStrepII

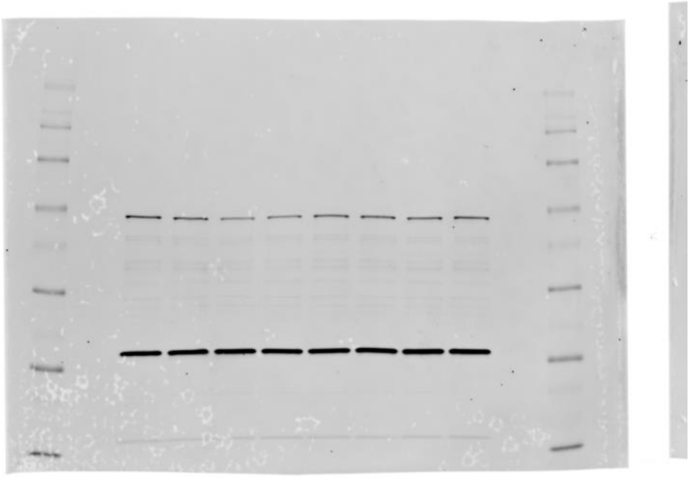

Fig S5 bot left aEspP$_{bN}$

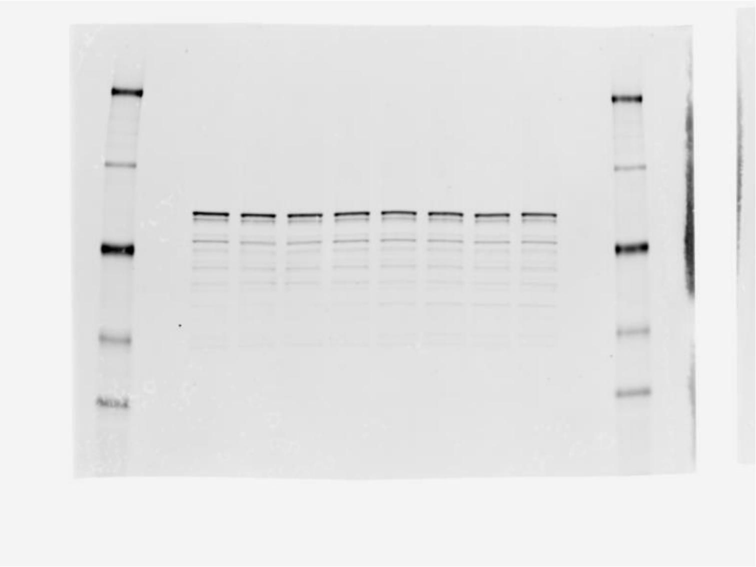

Fig S5 bot right aHis

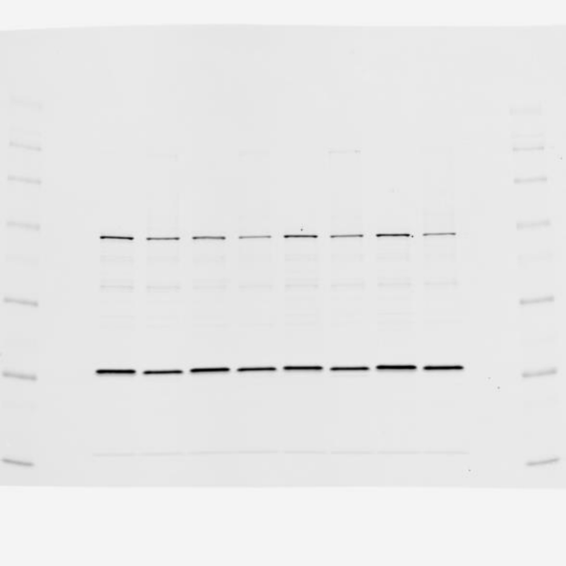

Fig S5 top left aEspP$_{bN}$

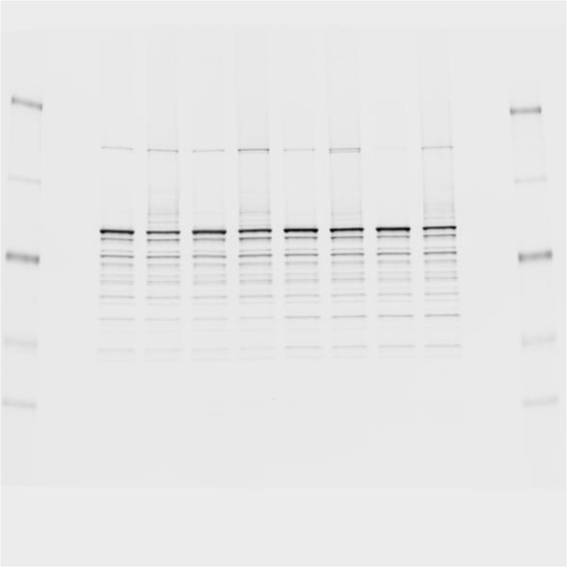

Fig S5 top right aHis

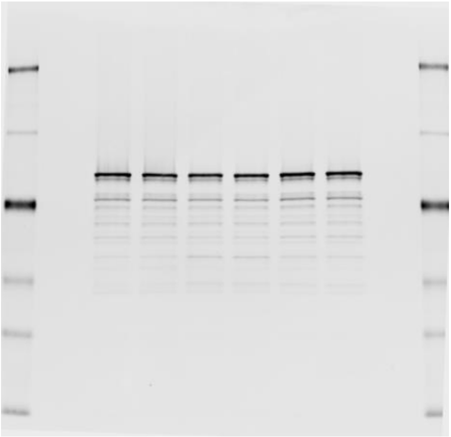

Fig S6a bot aHis

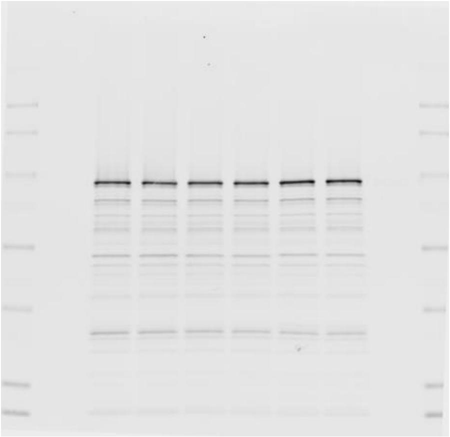

Fig S6a top aEspP$_{bC}$

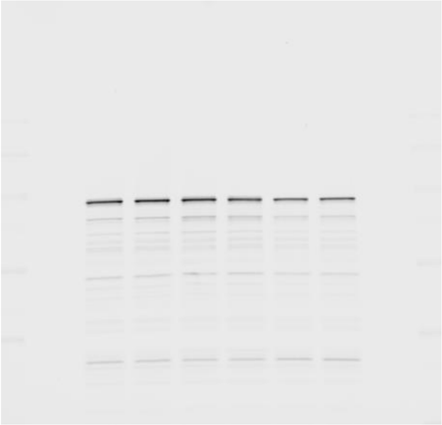

Fig S6c bot left aEspP$_{bC}$

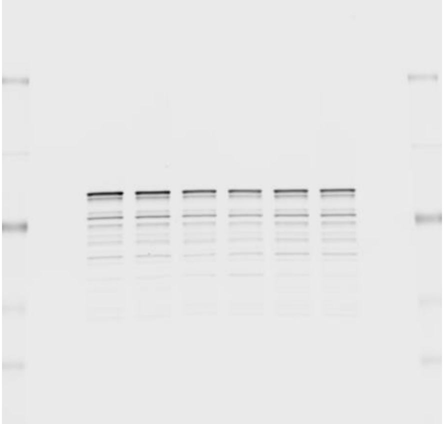

Fig S6c bot right aHis

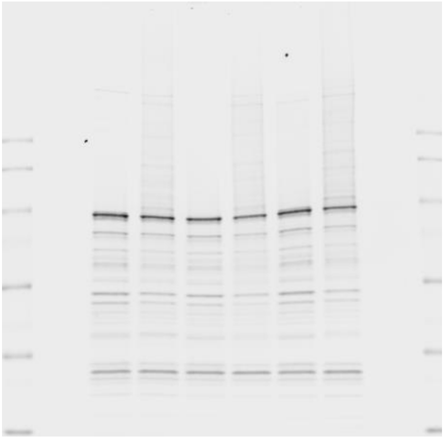

Fig S6c top left aEspP$_{bC}$

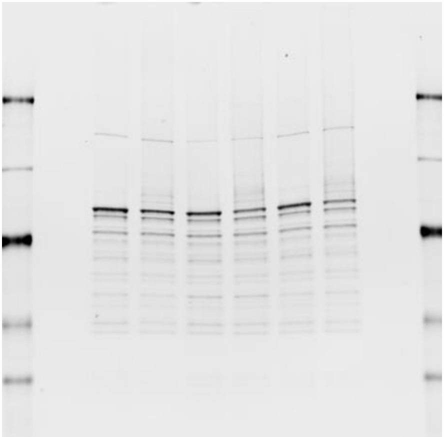

Fig S6c top right aHis

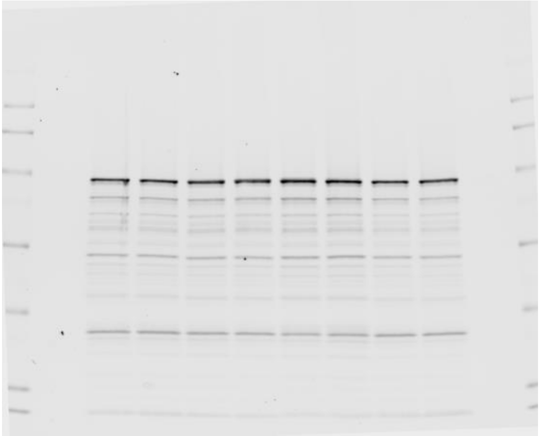

Fig S6d bot far left aEspP$_{bC}$

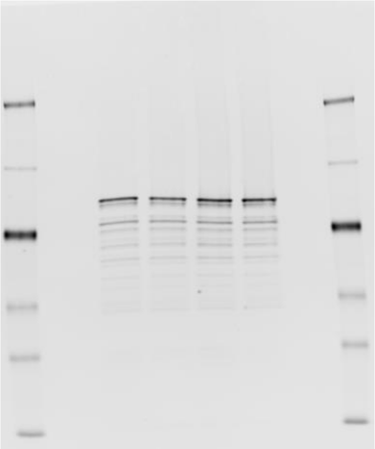

Fig S6d bot far right aHis

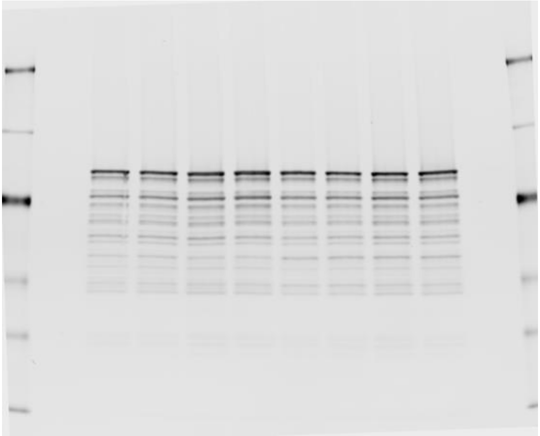

Fig S6d bot left aHis

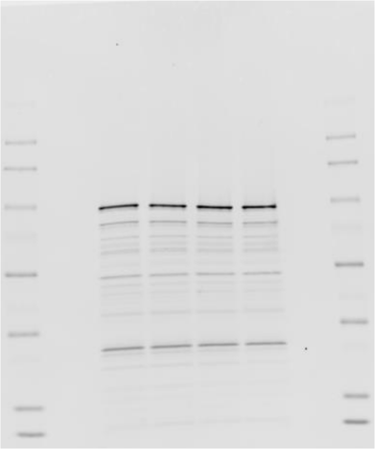

Fig S6d bot right aEspP_{bC}

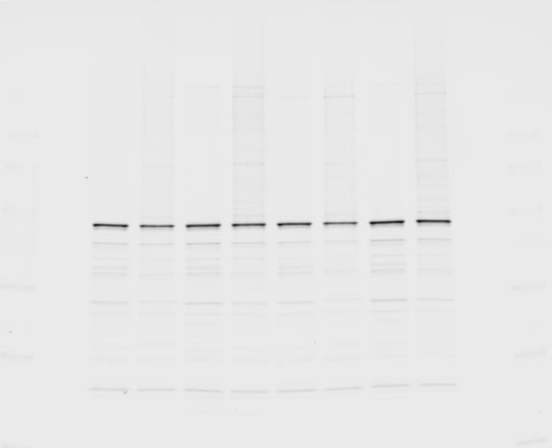

Fig S6d top far left aEspP$_{bC}$

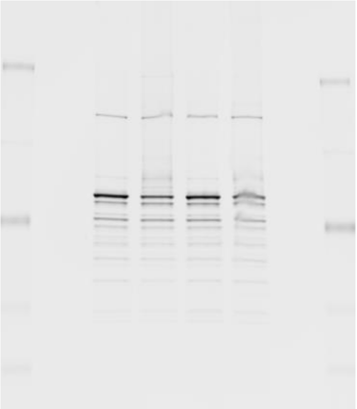

Fig S6d top far right aHis

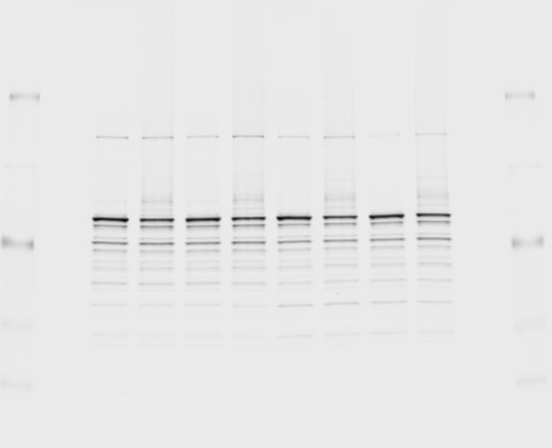

Fig S6d top left aHis

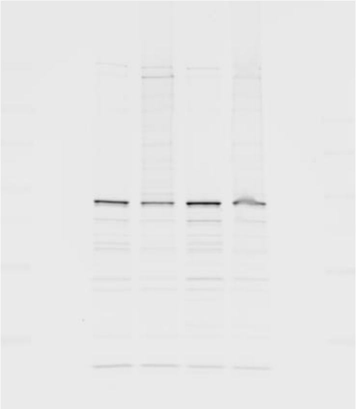

Fig S6d top right aEspP$_{bC}$

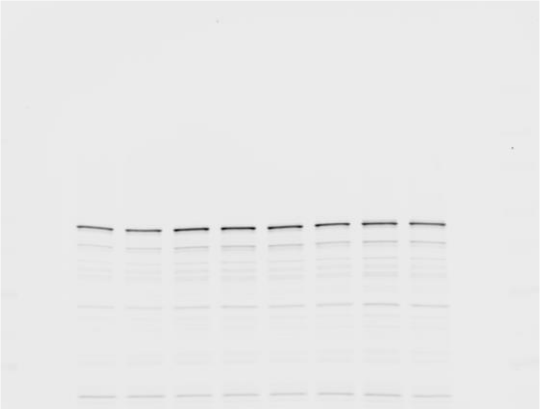

Fig S6e bot far left aEspP$_{bC}$

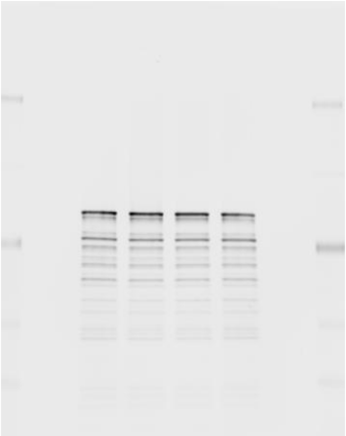

Fig S6e bot far right aHis

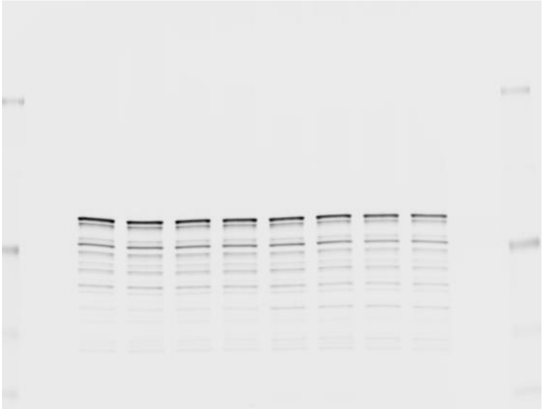

Fig S6e bot left aHis

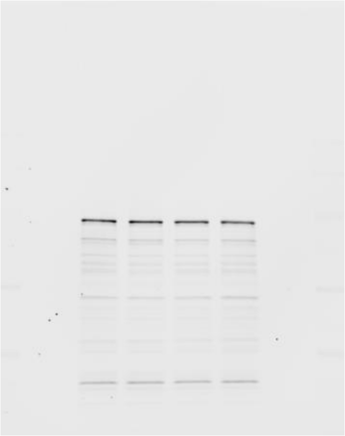

Fig S6e bot right aEspP$_{bC}$

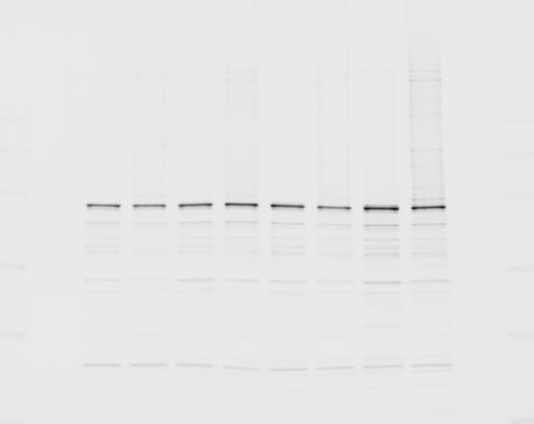

Fig S6e top far left aEspP$_{bC}$

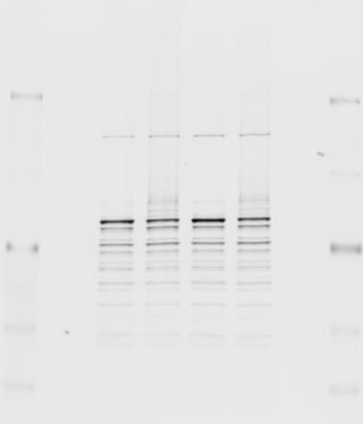

Fig S6e top far right aHis

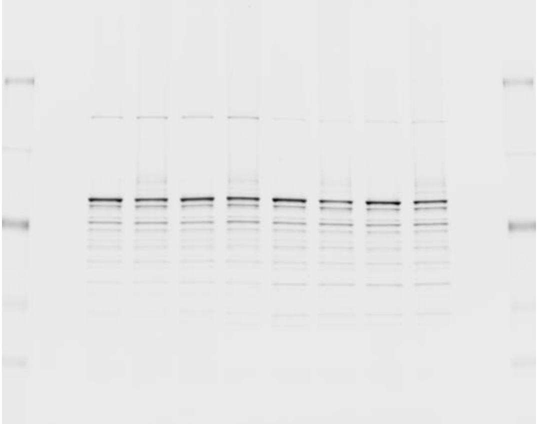

Fig S6e top left aHis

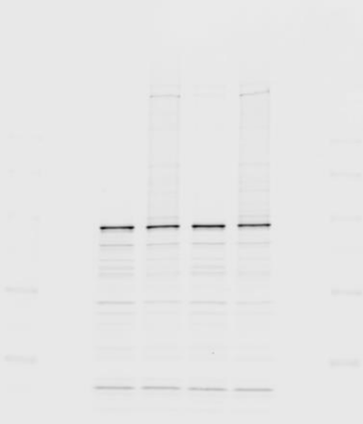

Fig S6e top right aEspP$_{bC}$

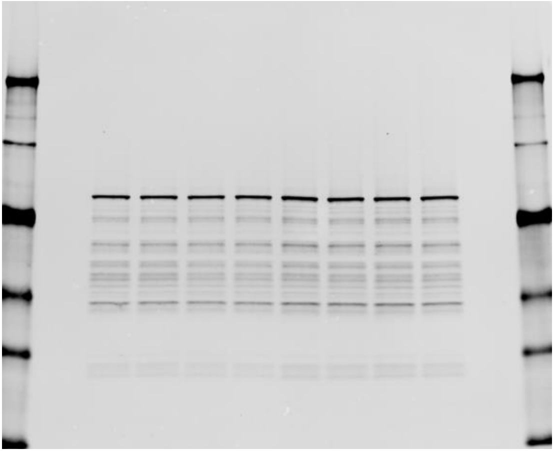

Fig S7a bot left aStrepII

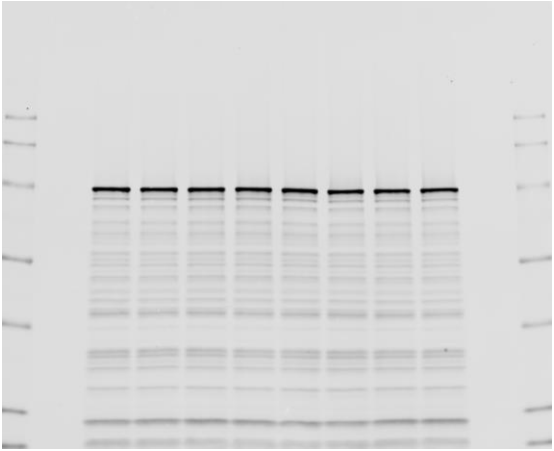

Fig S7a bot right aBamA$_C$

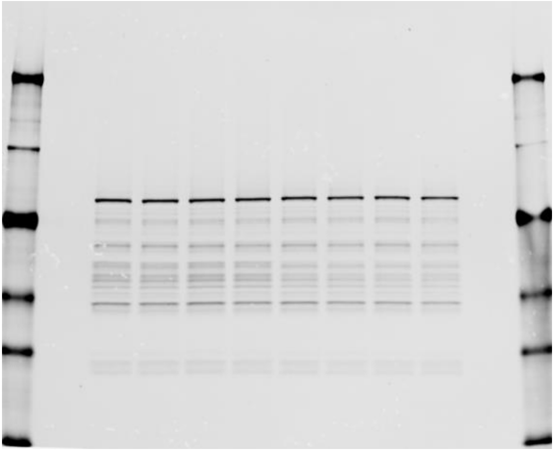

Fig S7a top left aStrepII

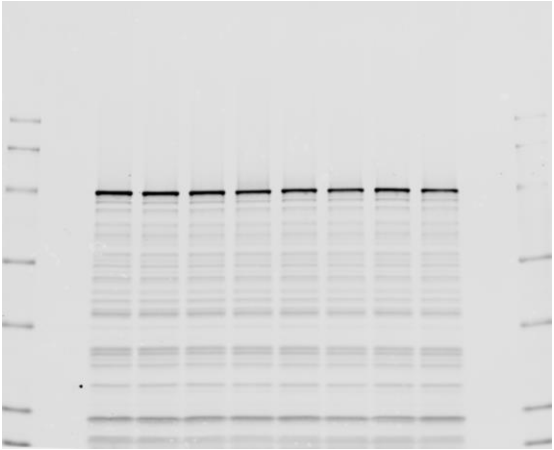

Fig S7a top right $aBamA_C$

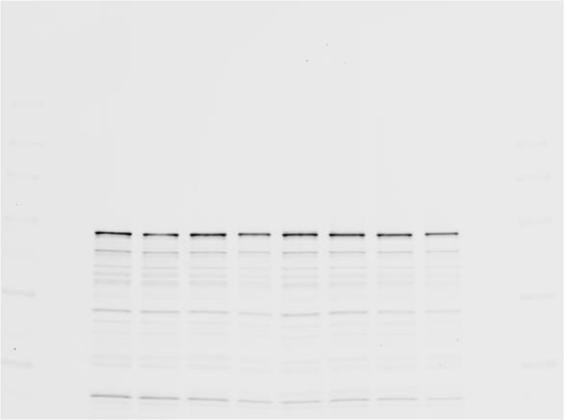

Fig S7b bot left aEspP$_{bC}$

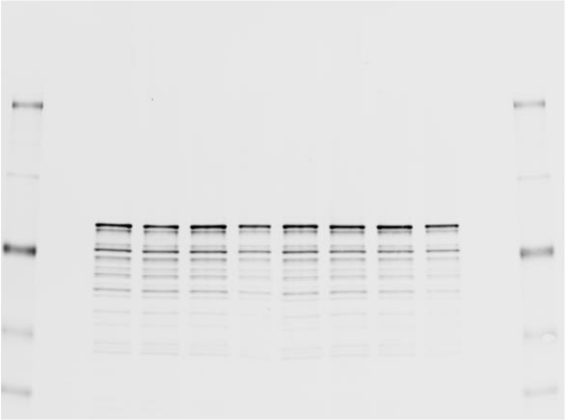

Fig S7b bot right aHis

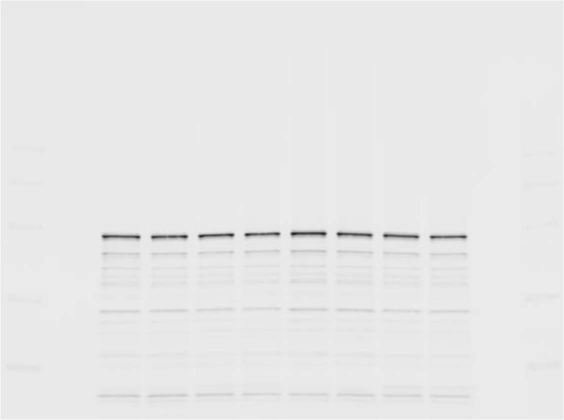

Fig S7b top left aEspP$_{bC}$

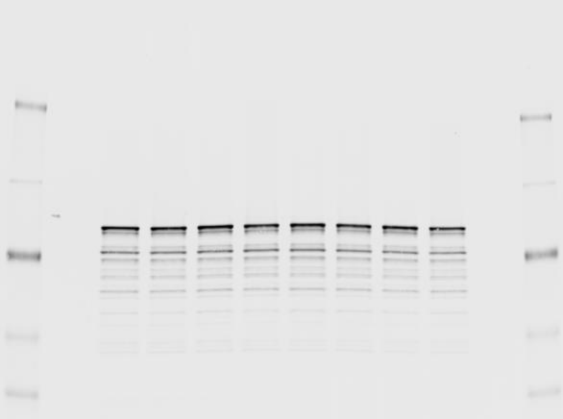

Fig S7b top right aHis

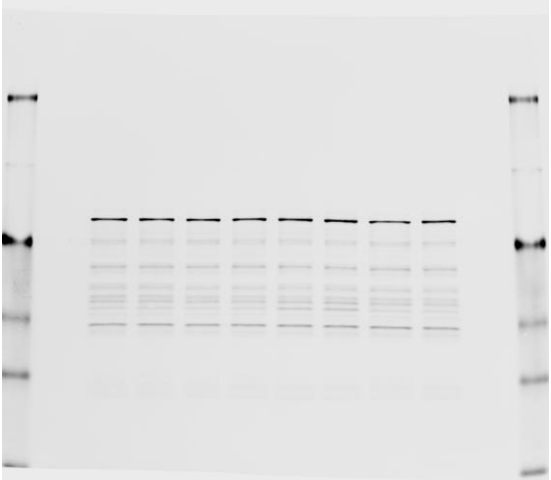

Fig S7d far bot left aStrepII

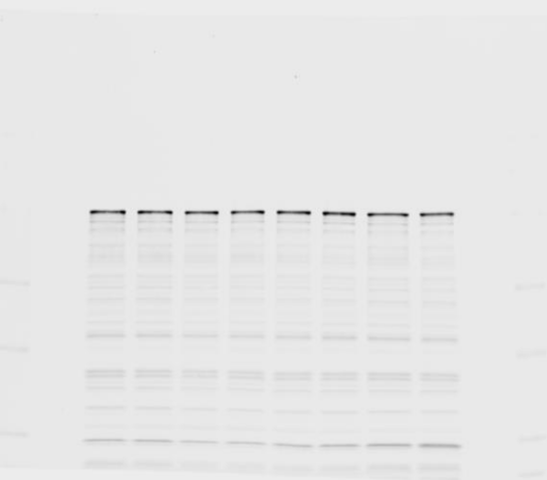

Fig S7d far bot right aBamA$_C$

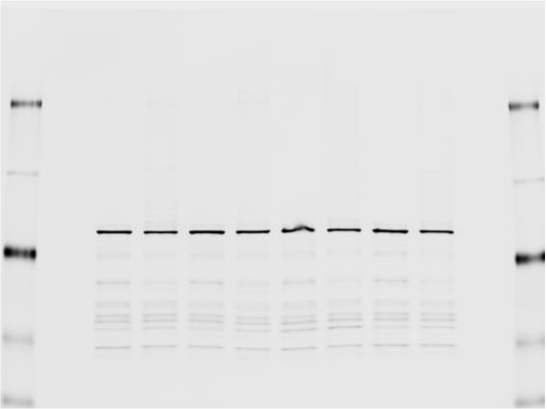

Fig S7d bot left aStrepII

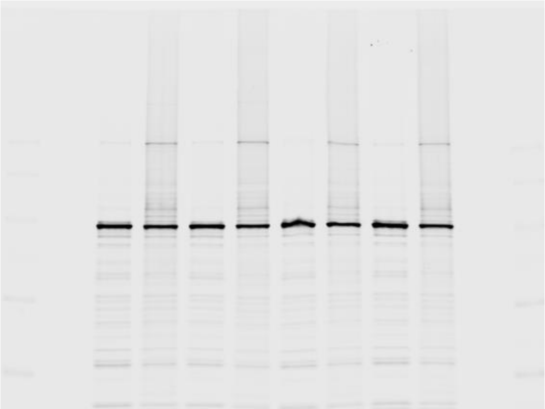

Fig S7d bot right $aBamA_C$

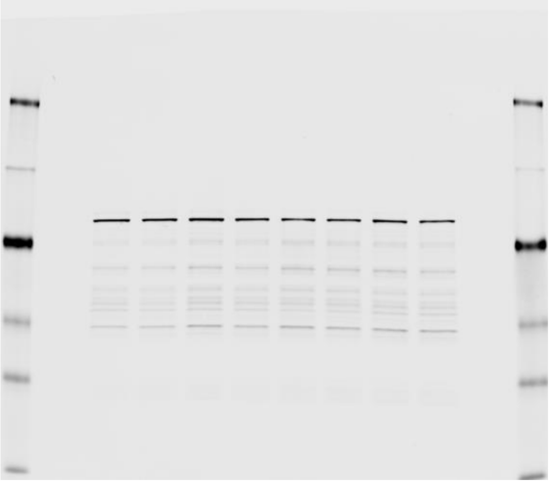

Fig S7d top left aStrepII

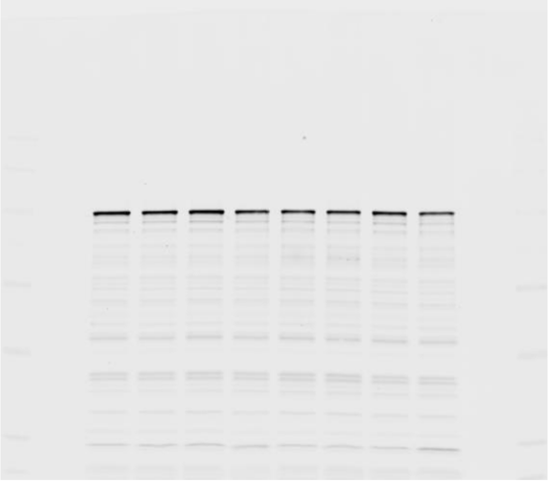

Fig S7d top right aBamA$_C$

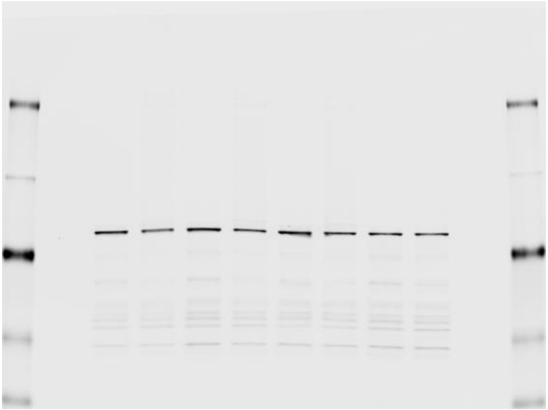

Fig S7d far top left aStrepII

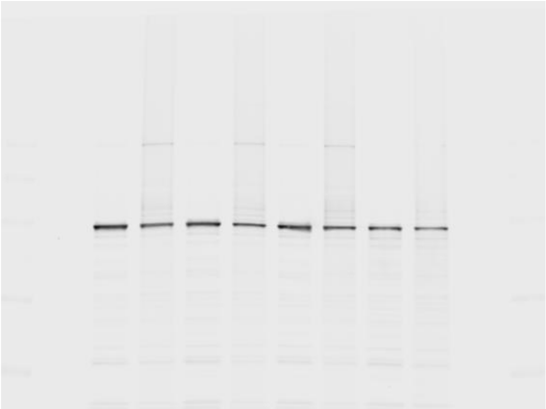

Fig S7d far top right aBamA$_C$

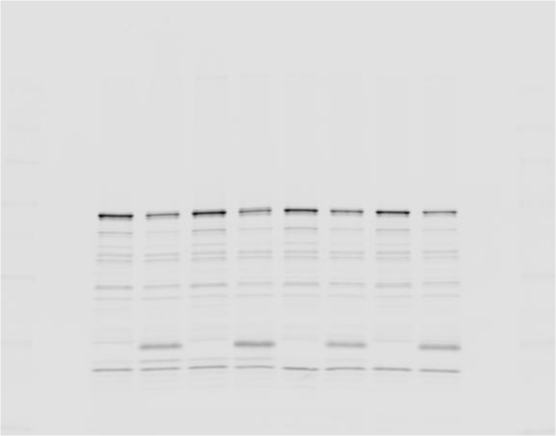

Fig S7e bot left $aEspP_{bC}$

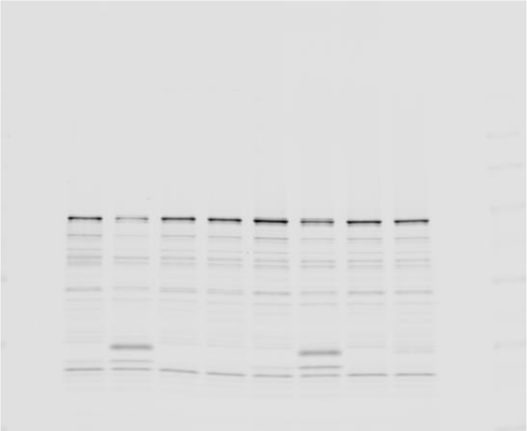

Fig S7e bot right aEspP$_{bC}$

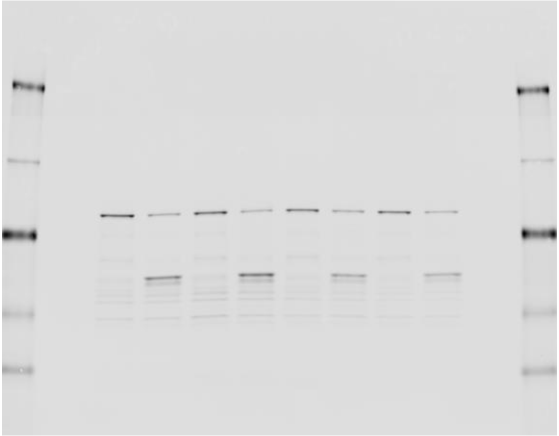

Fig S7e top left aStrepII

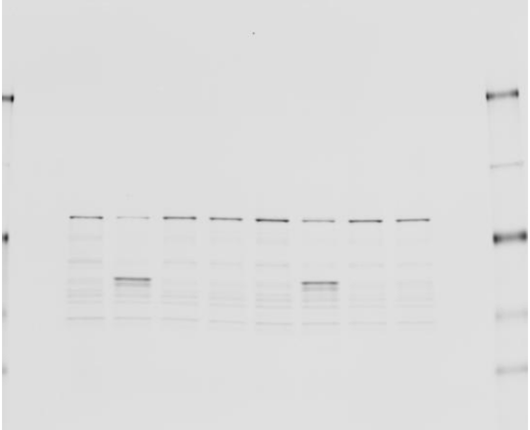

Fig S7e top right aStrepII

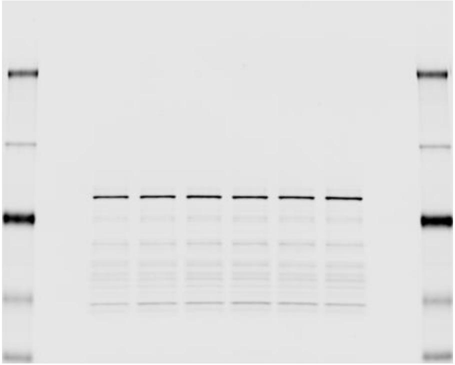

Fig S8 bot left aStrepII

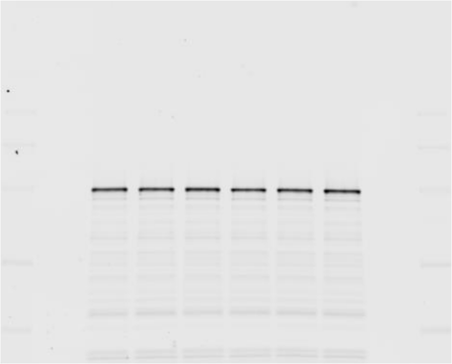

Fig S8 bot right aBamA$_C$

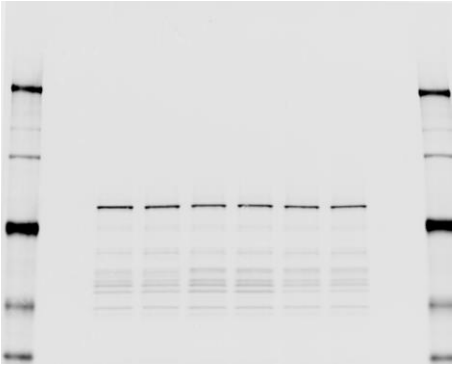

Fig S8 top left aStrepII

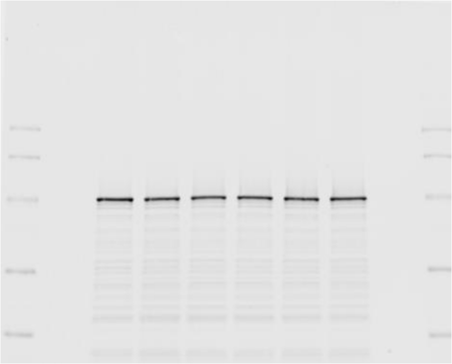

Fig S8 top right aBamA$_C$

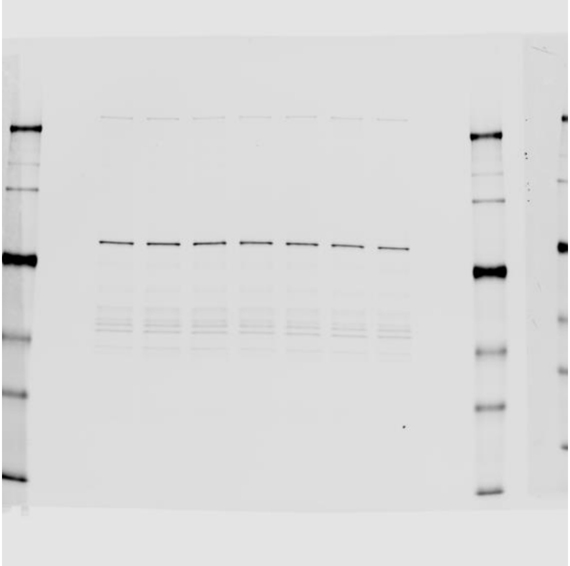

Fig S9a bot left aStrepII

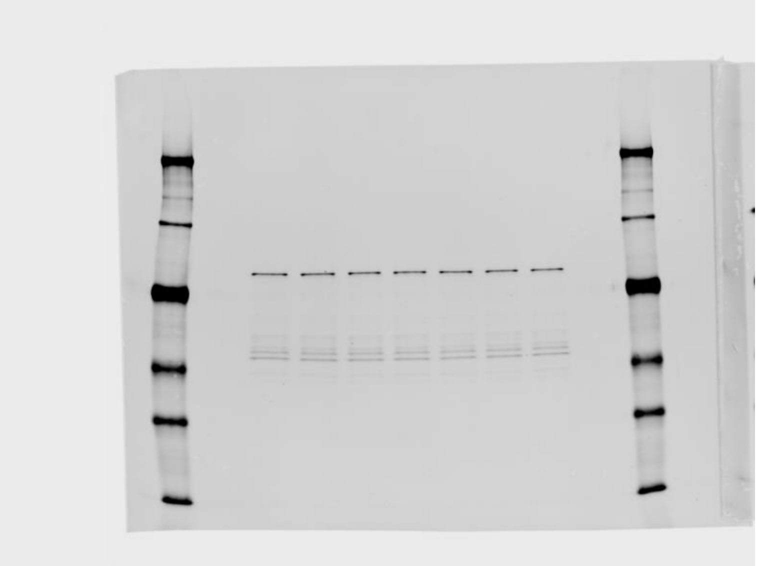

Fig S9a bot right aStrepII

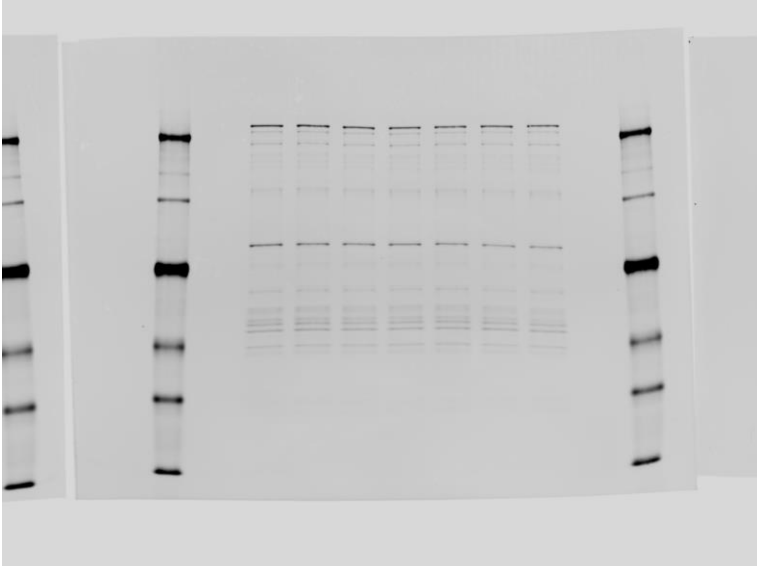

Fig S9a top left aStrepII

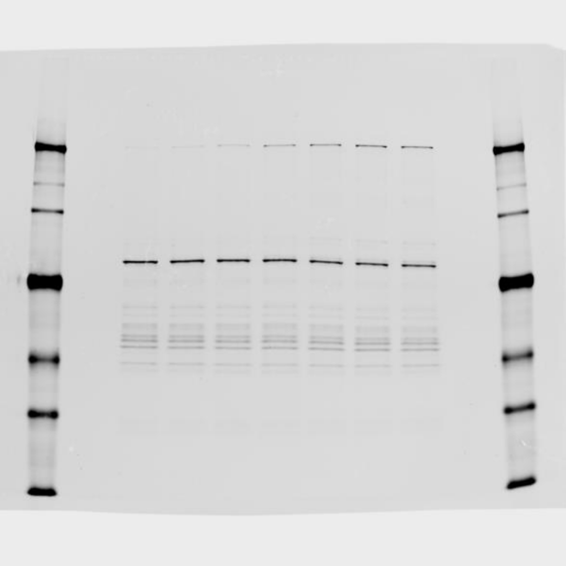

Fig S9a top right aStrepII

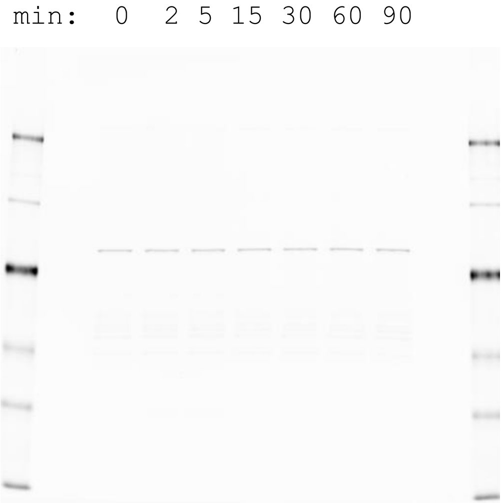

Fig S9b quant S1299C S425C rep 1 mock aStrepII

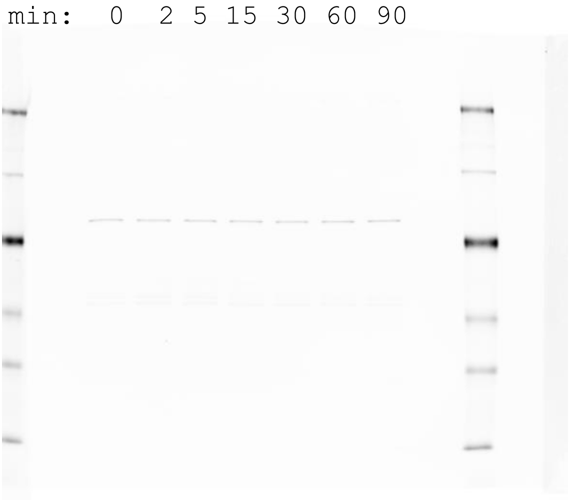

Fig S9b quant S1299C S425C rep 2 mock aStrepII

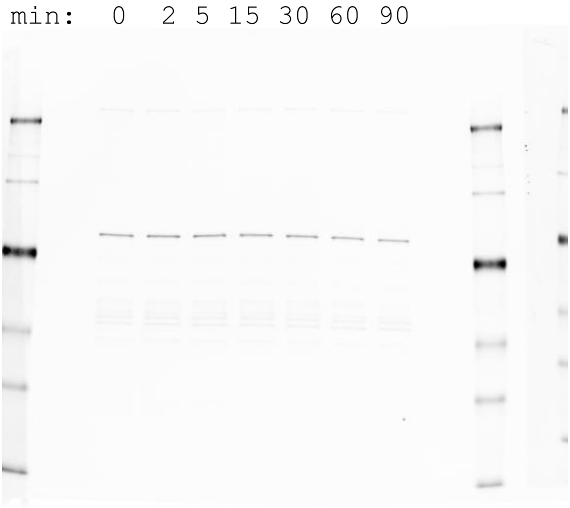

Fig S9b quant S1299C S425C rep 3 mock aStrepII

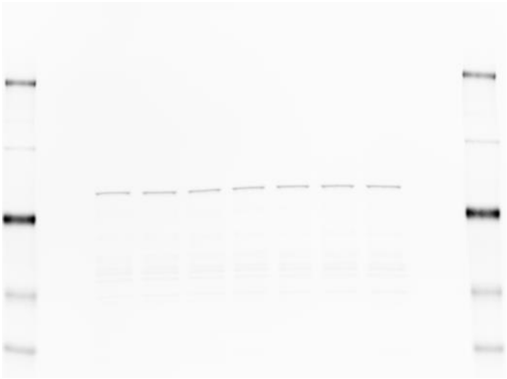

Fig S9b quant A1043C G781C rep 1 mock aStrepII

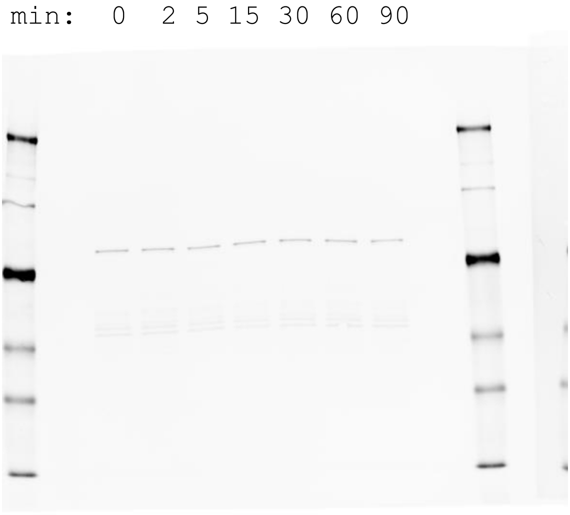

Fig S9b quant A1043C G781C rep 2 mock aStrepII

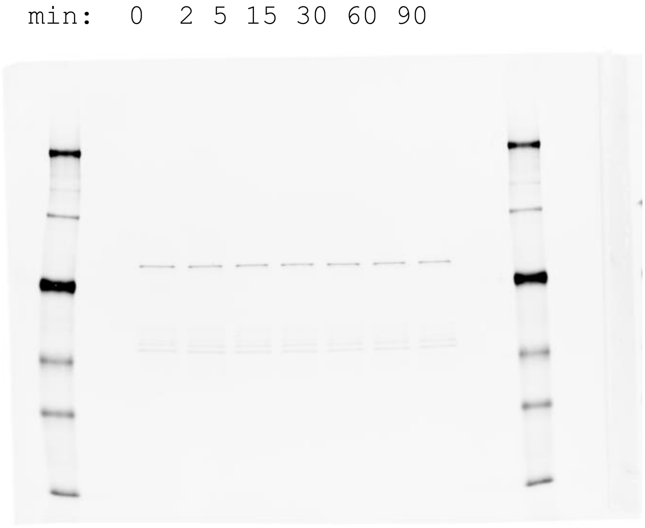

Fig S9b quant A1043C G781C rep 3 mock aStrepII

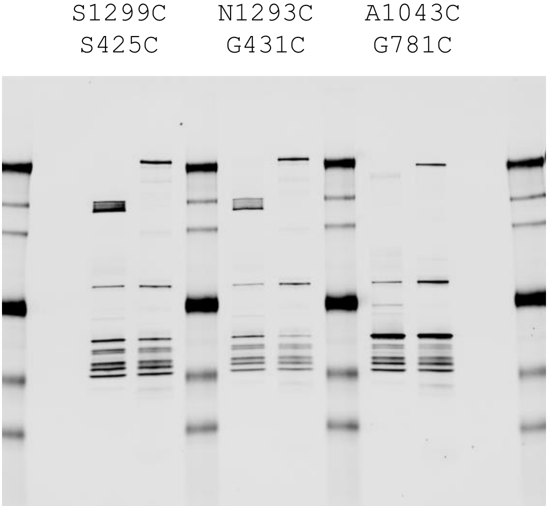

Fig S9c left aStrepII

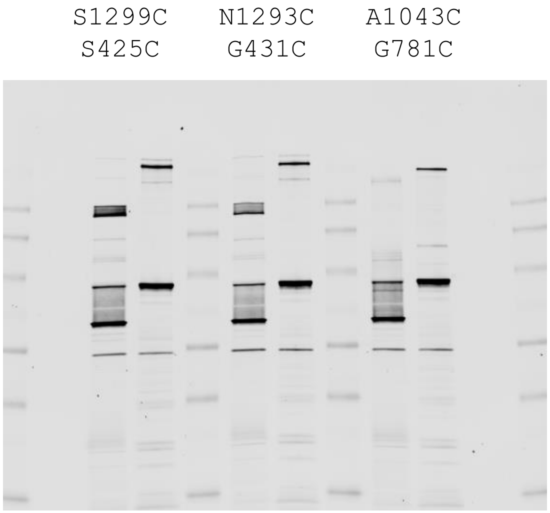

Fig S9c right $aBamA_C$

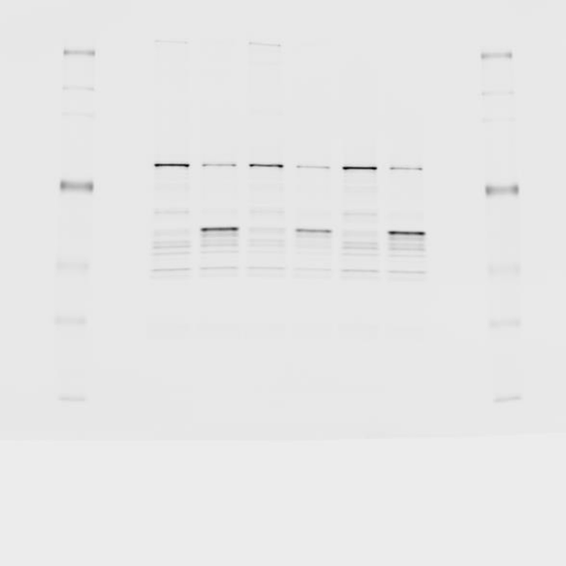

Fig S9d aStrepII
